# Ru3: a novel ruthenium-based compound overcomes cisplatin resistance in non-small cell lung cancer via the E2F2-IGFBP3-GPX4 pathway

**DOI:** 10.1007/s10495-026-02447-3

**Published:** 2026-10-03

**Authors:** Yiting Lu, Shiming Wu, Ying Jiang, Meishu Wang, Xinyu Liao, Jian Zhang, Junqi Xiang, Hua Yang, Min Yu, Jiang Min, Chen Fu, Yunlong Lei

**Affiliations:** 1 Department of General Surgery, Chongqing Academy of Medical Sciences, Chongqing General Hospital, School of Medicine, Chongqing University, Chongqing, 401120 China; 2 Department of Biochemistry and Molecular Biology, and Molecular Medicine and Cancer Research Center, College of Basic Medical Sciences, Chongqing Medical University, Chongqing, 400016 People’s Republic of China; 3 Chongqing Health Center for Women and Children, Chongqing, 400700 PR China; 4 College of Pharmaceutical Sciences, Southwest University, Chongqing, 400715 PR China; 5 Gastrointestinal Surgery Department, The First Affiliated Hospital of Chongqing Medical University, Chongqing, 400016 China; 6 Tianfu Jincheng Laboratory, Chengdu, 610212 China; 7 Chongqing Vocational College of Intelligent Engineering, Chongqing, 402100 China

**Keywords:** Ruthenium, Cisplatin resistance lung cancer, ROS, IGFBP3, E2F2

## Abstract

Cisplatin resistance remains a major hurdle in non-small cell lung cancer (NSCLC) treatment. Here, we designed and synthesized a novel ruthenium-based compound, which selectively kills cisplatin-resistant NSCLC cells while sparing parental cells. Mechanistically, Ru3 triggers mitochondrial damage and reactive oxygen species (ROS) accumulation. In addition, it upregulates the transcription factor E2F2, which directly represses IGFBP3 expression. IGFBP3 normally scavenges ROS via the GPX4 system; thus, E2F2-mediated IGFBP3 suppression disrupts this antioxidant defense, exacerbating ROS levels and cell death. Notably, cisplatin-resistant cells exhibit elevated GPX4 and IGFBP3 expression, making them particularly vulnerable to Ru3. This study elucidates the E2F2-IGFBP3-GPX4 axis as a novel therapeutic vulnerability and highlights Ru3 as a promising agent for refractory NSCLC.

**Graphical abstract:**

**Figure:**
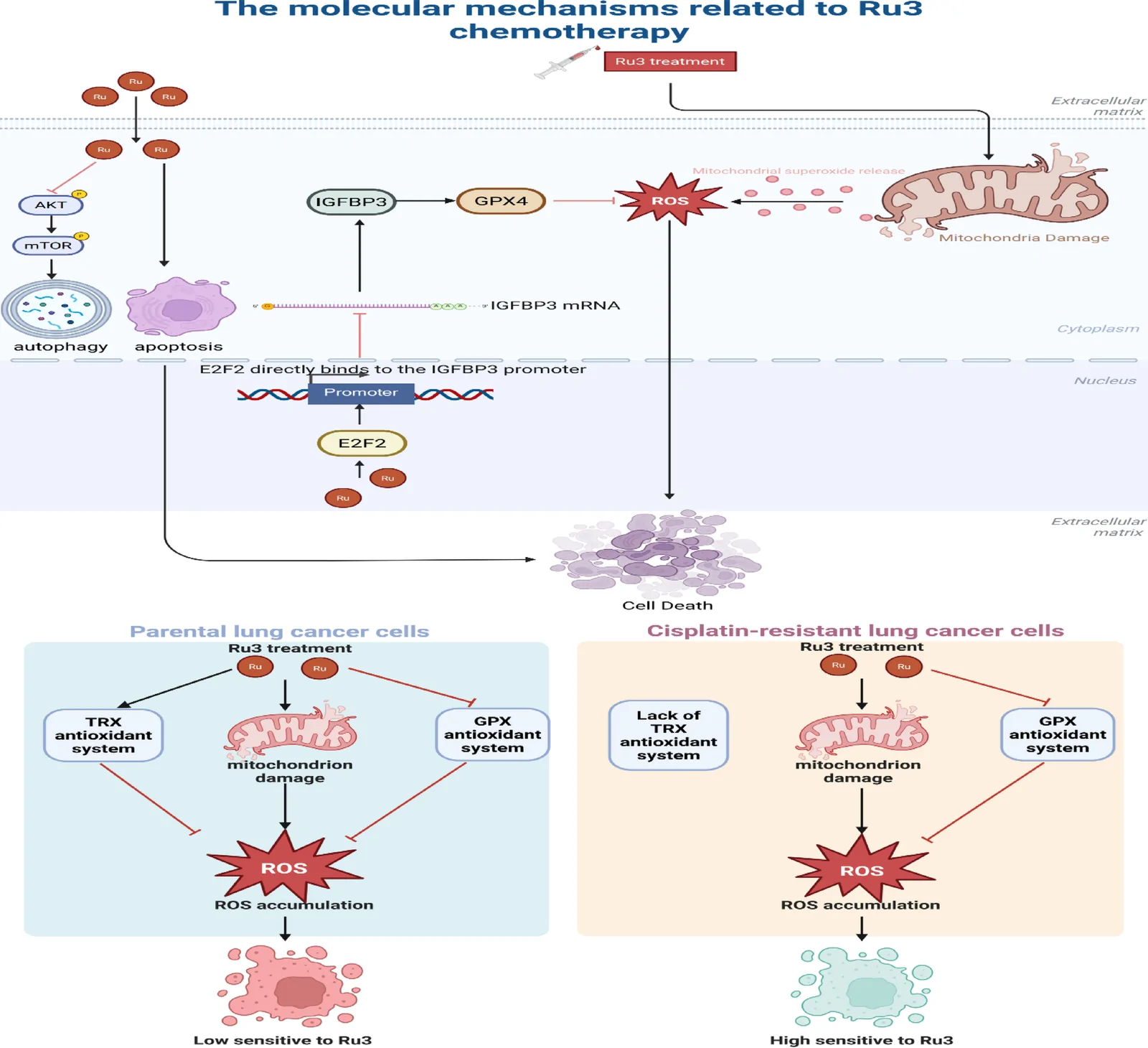

Ru3's Mechanism of Action on Cisplatin Resistant Lung Cancer Cells

1. Ru3 induces mitochondrial damage, which promotes the accumulation of ROS and ultimately triggers cell death.
2. Ru3 upregulates E2F2, enabling it to directly bind to the IGFBP3 promoter and suppress IGFBP3 expression, which inhibits GPX4 expression and further exacerbating lethal ROS accumulation .
3. Treatment with Ru3 resulted in significant cell death in cisplatin-resistant lung cancer cells, yet had a minimal impact on parental lung cancer cells, which attributed to the suppression of the TRX antioxidant system in cisplatin-resistant cells, coupled with an increase in the activity of the GSH antioxidant system.

**Supplementary Information:**

The online version contains supplementary material available at https://doi.org/10.1007/s10495-026-02447-3.

## Introduction

Lung cancer remains a formidable public health challenge, ranking prominently among the leading causes of cancer-related mortality worldwide [1–3]. Despite advancements in diagnostic techniques and therapeutic interventions, its incidence continues to escalate [4]. The lung cancer is broadly categorized into small cell lung cancer (SCLC) and non-small cell lung cancer (NSCLC), with NSCLC comprising the majority of cases [5]. Among NSCLC subtypes, adenocarcinoma and squamous cell carcinoma are the most prevalent, exhibiting distinct biological and clinical characteristics that necessitate tailored diagnostic and therapeutic approaches [6].

Chemotherapy, particularly regimens centered on cisplatin, has long been a cornerstone in the management of lung cancer [6]. Cisplatin exerts its antitumor effects by inducing DNA damage, leading to cell cycle arrest and apoptosis [7]. However, its clinical utility is often limited by toxicity and the development of drug resistance [8]. In recent years, other transition-metal complexes, especially ruthenium-based compounds, have emerged as promising antitumor agents with potentially lower toxicity [9, 10]. Unlike platinum-based drugs, ruthenium complexes may exert anticancer effects through diverse mechanisms, including direct DNA binding, disruption of cellular redox balance, and modulation of signaling pathways, thereby offering potential efficacy against cisplatin-resistant cancers [10, 11]. Encouraging clinical trial results have been reported for ruthenium-based chemotherapeutics, though further validation and optimization are required before widespread clinical application [12]. In our study, we designed and screened a novel ruthenium-based compound (Ru3) and investigated its anticancer effects and molecular mechanisms.

Reactive oxygen species (ROS) play a pivotal role in tumor resistance to therapy, acting as both mediators of cell death and inducers of adaptive mechanisms in cancer cells [13]. Elevated ROS levels can trigger apoptosis, necrosis, and other forms of cell death, making ROS-inducing strategies promising for cancer treatment [14–16]. However, cancer cells often develop resistance to ROS-mediated therapies through various mechanisms, including upregulation of antioxidant systems, alterations in ROS-generating enzymes, and modulation of cellular signaling pathways. These adaptive changes allow cancer cells to survive and proliferate despite high ROS levels [14–16]. Cisplatin targets thioredoxin reductase (TrxR), causing intracellular ROS accumulation that ultimately leads to growth arrest and cell death [7]. In response to higher ROS levels and to evade cisplatin-triggered cell death, cisplatin-resistant cancer cells diminish their utilization of TRX and activate other antioxidant pathways to make up for its absence [7]. Accumulating evidence shows that cisplatin-resistant cells exhibit elevated glutathione (GSH) levels, suggesting that GSH system targeting could be an effective therapeutic strategy [17]. Therefore, targeting the GSH system may be an effective strategy for treating cisplatin-resistant tumors.

Insulin-like growth factor binding protein-3 (IGFBP3) has recently garnered attention as a potential therapeutic target in cancer [18]. IGFBP3, a key component of the insulin-like growth factor (IGF) system, traditionally functions as a carrier and modulator of IGF bioactivity [19]. However, recent evidence suggests that IGFBP3 also possesses IGF-independent actions, including direct antitumor effects [20]. Low levels of IGFBP3 expression have been linked to poor prognosis in several cancer types, including hepatocellular carcinoma [21]. In addition, IGFBP3 has also been reported to differentially regulate mitochondrial ROS accumulation in cancer therapy [14, 22]. Despite its significant potential, the precise role of IGFBP3 in cancer pathogenesis remains largely unexplored.

E2F2, a critical regulator of cell cycle progression and differentiation, has been implicated in the modulation of various biological processes pertinent to cancer development [23, 24]. Its deregulation has been observed in numerous malignancies, where it exerts multifaceted effects on tumor cell proliferation, apoptosis resistance, and invasive capabilities [25]. E2F2 has also been reported to be involved in cancer therapeutics to induce ROS accumulation [26]. Our study reveals that ruthenium-based chemotherapeutic Ru3 exerts potent anti-proliferative activity against cisplatin-resistant lung cancer cells but exhibits weak inhibitory effects on parental cells. Mechanistically, Ru3 elevates E2F2 to downregulate IGFBP3 and repress the GSH antioxidant system. Additionally, Ru3 provokes severe mitochondrial injury and ROS leakage,eventually drives robust ROS accumulation and cancer cell death. These findings provide novel insights for developing therapeutic strategies against cisplatin-resistant lung cancer.

## Results

### Synthesis pathways of ruthenium element compounds

**Fig. 1:**
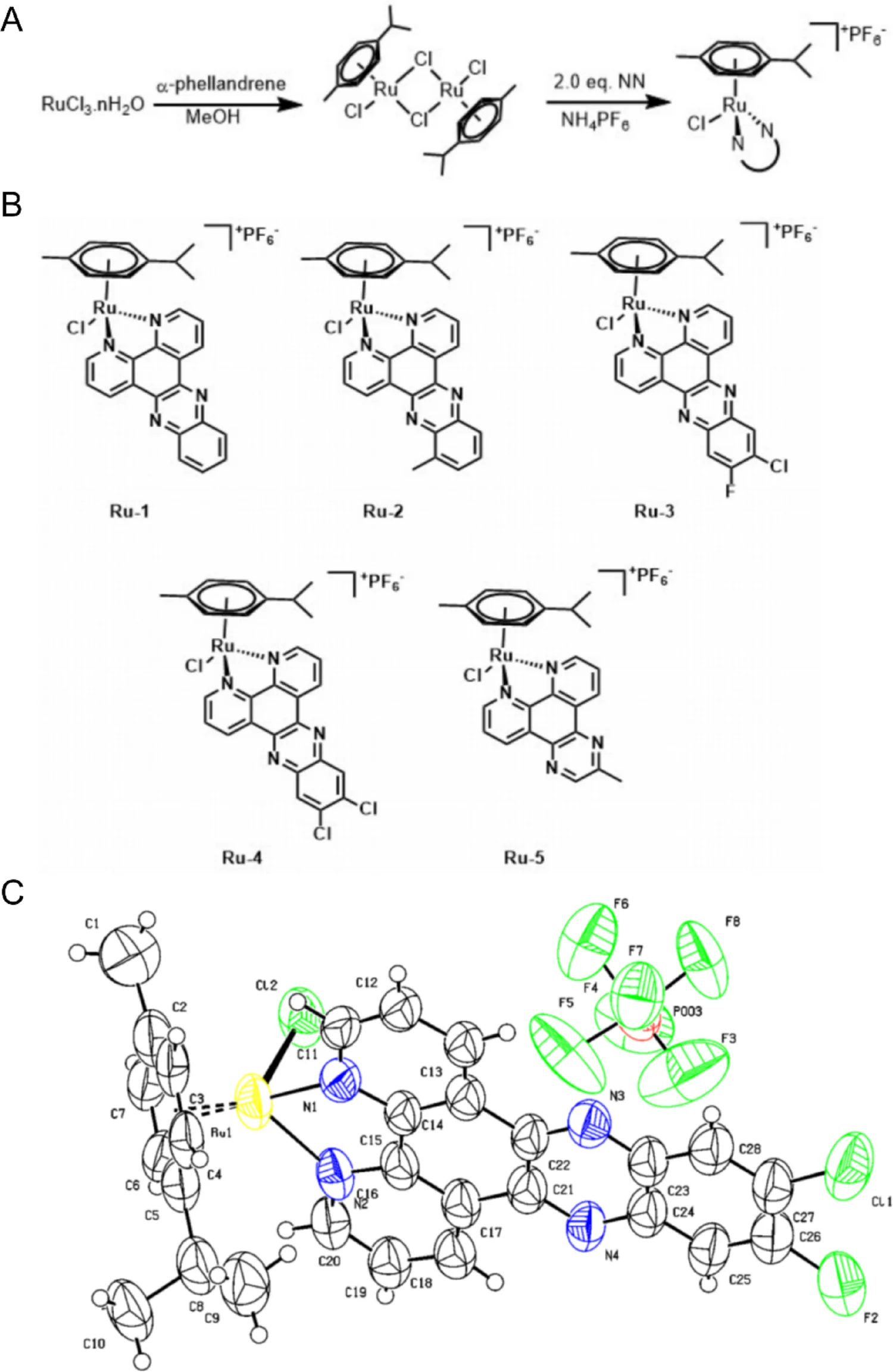
Chemical synthesis **A** Synthetic routes for ruthenium dimer precursor [(η6-p-cymene)Ru (μ-Cl)Cl]2 and half-sandwich ruthenium(Ⅱ) complexes Ru-1~5. **B** Chemical structures of ruthenium(II) complexes Ru-1~5. **C** Crystal structure of complex Ru3

The overall synthetic route is shown in Fig. 1A. The ruthenium (Ⅱ) dimer precursor [(*η*^*6*^*-p*-cymene)Ru(μ-Cl)Cl]_2_ was synthesized according to the reported procedures with modification [27]. Polypyridyl ligands (NN) with varying heterocyclic π-systems were obtained from the reaction of 1,10-phenanthroline−5,6-dione and phenyldiamine with different substituents. Ligand-exchange reactions were subsequently carried out to produce the half-sandwich ruthenium(II) complexes **Ru-1~5** (Fig. 1B). Typically, the ruthenium(Ⅱ) precursor [(*η*^*6*^*-p*-cymene)Ru(μ-Cl)Cl]_2_ reacted with 2.0 equiv of NN ligand in degassed chloroform: absolute methanol (1:1) solution at 40 ℃ in reaction flask for 4 h, the resulting suspension was precipitated by the addition of excess ammonium hexafluorophosphate (NH_4_PF_6_), after silica gel column chromatography purification and drying, relevant [Ru(*η*^*6*^*-p*-cymene)Cl(NN)]PF_6_ could be obtained as a solidate powder in proper yield.

In general, DMSO is used as the solvent to prepare the stock solutions for all the complexes. Hence, we monitored the stability of the complexes in both deuterated MeCN and DMSO before all the anti-cancer experiments. Importantly, this kind of half-sandwich aromatic ruthenium(II) complex exhibited remarkable stability whether in solution or solid-state monitored by ^1^H NMR over one month. Furthermore, the structure of compound RU-3 was confirmed unequivocally by X-ray crystallographic analysis (Fig. 1C).

### Ru3 effectively inhibits proliferation and migration in cisplatin-resistant lung cancer cells

**Fig. 2:**
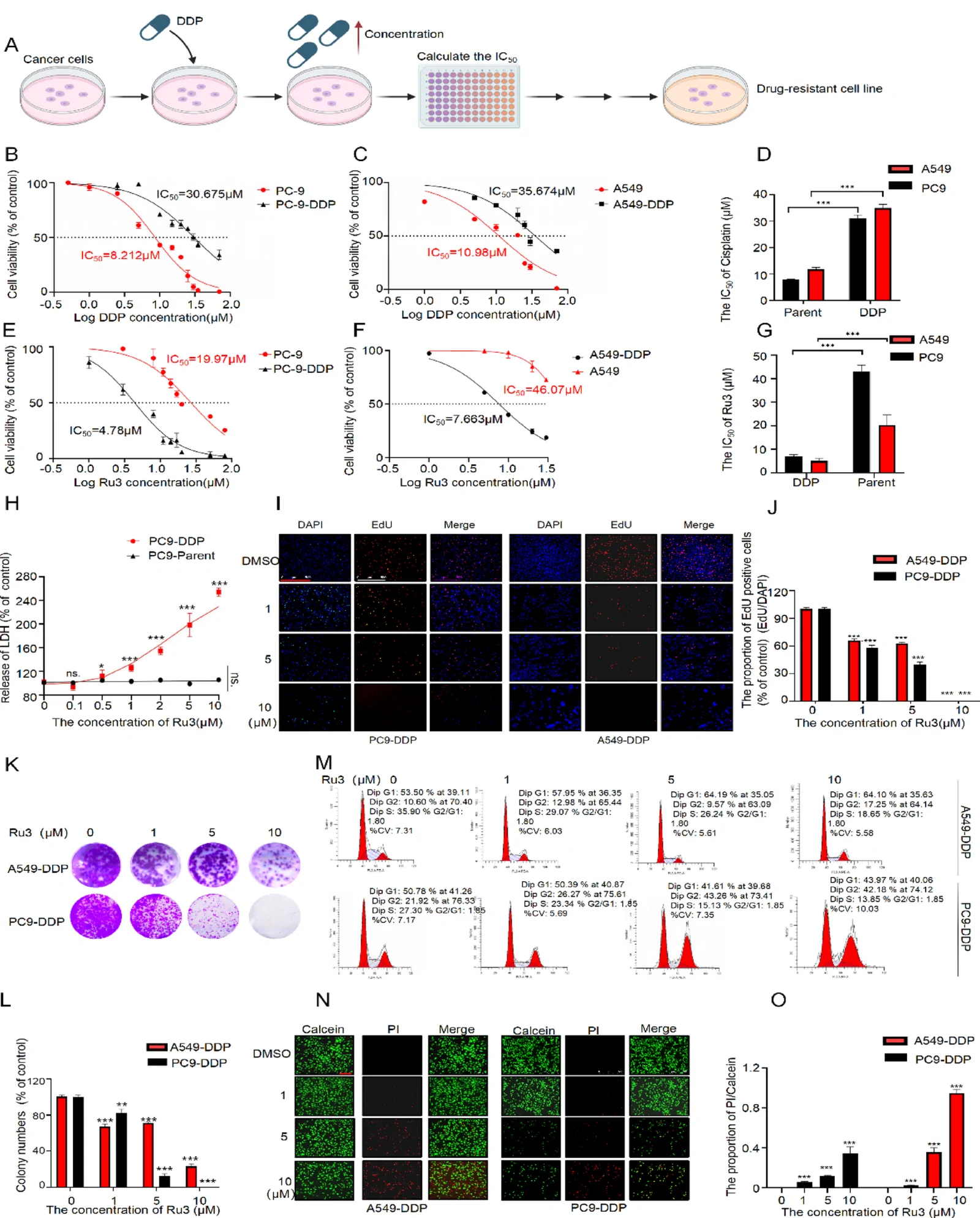
Ru3 prominently inhibits proliferation in cisplatin resistant lung cancer cells **A** Schematic diagram illustrating the establishment of cisplatin-resistant lung cancer cell lines. **B**–**D** PC9 parental and PC9-DDP cells, as well as A549 parental and A549-DDP cells, were treated with the indicated concentrations of cisplatin for 24 h, and cell viability was subsequently determined using the CCK-8 kit. **E**–**G** These cell lines were treated with the indicated concentrations of Ru3 for 24 h, and cell viability was again assessed using the CCK-8 kit. **H** Lactate dehydrogenase (LDH) release was analyzed in PC9 parental and PC9-DDP cells treated with the indicated concentrations of Ru3 for 24 h. Cell lysates served as the positive control. **I**–**J** EdU incorporation assays were performed on A549-DDP and PC9-DDP cells treated with the indicated concentrations of Ru3 for 24 h; EdU-positive cells were quantified. Scale bar: 250 μm. **K**–**L** Colony formation assays were performed to evaluate cell proliferation. A549-DDP and PC9-DDP cells were treated with the indicated concentrations of Ru3 for 24 h. After treatment, cells were seeded into 12-well plates and allowed to grow for two weeks, after which colonies were stained and counted. **M** A549-DDP and PC9-DDP cells were treated with the indicated concentrations of Ru3 for 24 h and then subjected to flow cytometry analysis to determine the cell cycle distribution. **N**–**O** PC9-DDP and A549-DDP cells were treated with the indicated concentrations of Ru3 for 24 h, followed by staining with Calcein-AM and propidium iodide (PI) to calculate the ratio of live to dead cells. Scale bar: 250 μm. Data are presented as means ± s.d. and are representative of three independent experiments. *, *P* < 0.05; **, *P* < 0.01; ***, *P* < 0.001. Statistical significance was compared with the respective control groups

We initiated our study by successfully establishing two cisplatin-resistant lung cancer cell lines, A549-DDP and PC9-DDP (Fig. 2A–D). Western blot analysis revealed that ERCC1—a core protein mediating DNA damage repair, whose overexpression drives chemoresistance across various malignancies via efficient repair of platinum-triggered DNA crosslinks [28]—was markedly upregulated in A549-DDP and PC9-DDP cells relative to their parental counterparts (Figure S1A), thus further validating the successful establishment of cisplatin-resistant phenotypes at the molecular level. Subsequently, we employed a CCK-8-based cell proliferation assay to evaluate the inhibitory efficacy of various structurally analogous ruthenium compounds against these cisplatin-resistant cell lines. Notably, Ru3 emerged as the most potent inhibitor, exhibiting the lowest IC_50_ value among all tested compounds (Figures S1B-D). Intriguingly, when Ru3 was administered to both parental and cisplatin-resistant lung cancer cells, we observed a marked difference in its inhibitory effect. Specifically, Ru3 demonstrated a significantly greater ability to inhibit the proliferation of cisplatin-resistant cells and elicited significant cytotoxicity compared to their parental counterparts (Fig. 2E–H). This unexpected finding underscored the unique efficacy of Ru3 against drug-resistant cancer cells. To further substantiate the antiproliferative activity of Ru3, we assessed colony formation and EdU incorporation in cisplatin-resistant lung cancer cells treated with Ru3. Both assays revealed a significant reduction in colony formation and EdU incorporation, indicative of impaired cell proliferation (Fig. 2I–L). Meanwhile, we found that Ru3 exerted markedly stronger tumor-killing effects at equivalent concentrations compared with irinotecan and docetaxel, two first-line alternative chemotherapeutic agents clinically administered for cisplatin-resistant lung cancer (Figure S1E). Furthermore, Ru3 exhibited relatively weak cytotoxicity toward the normal lung epithelial cell line NL20, as evidenced by its higher IC₅₀ value of 17.92 μM (Figure S1F). Consistently, a panel of assays including EdU proliferation assay, Calcein-AM/PI double staining and LDH release assay further confirmed that Ru3 elicited mild inhibitory effects on cell proliferation and weak cytotoxicity in NL20 normal lung epithelial cells (Figures S1G–K).

Subsequently, we further explored whether Ru3 could suppress the migratory and metastatic capacities of cisplatin-resistant lung cancer cells. Results from Transwell migration assays and wound healing assays robustly validated that Ru3 markedly impeded the migratory potential of both cell lines (Figures S1L–O).

Flow cytometry analysis revealed G_2_ phase cell cycle arrest in Ru3-treated tumor cells (Fig. 2M). This was corroborated by RT-PCR and Western blot analyses, demonstrating upregulation of P53-related genes and downregulation of CDK1 expression (Figures S1P-Q). To evaluate the cytotoxic potential of Ru3, Calcein-AM/PI double staining revealed dose-dependent accumulation of dead cells (Fig. 2N–O).

In summary, our findings highlight the remarkable antiproliferative activity of Ru3 against cisplatin-resistant lung cancer cells, accompanied by relatively mild cytotoxicity toward normal lung epithelial cells, suggesting that Ru3 may represent a promising therapeutic candidate for combating cisplatin-resistant lung cancer.

### Ru3 induces apoptosis in cisplatin-resistant lung cancer cells both in vitro and in vivo

**Fig. 3:**
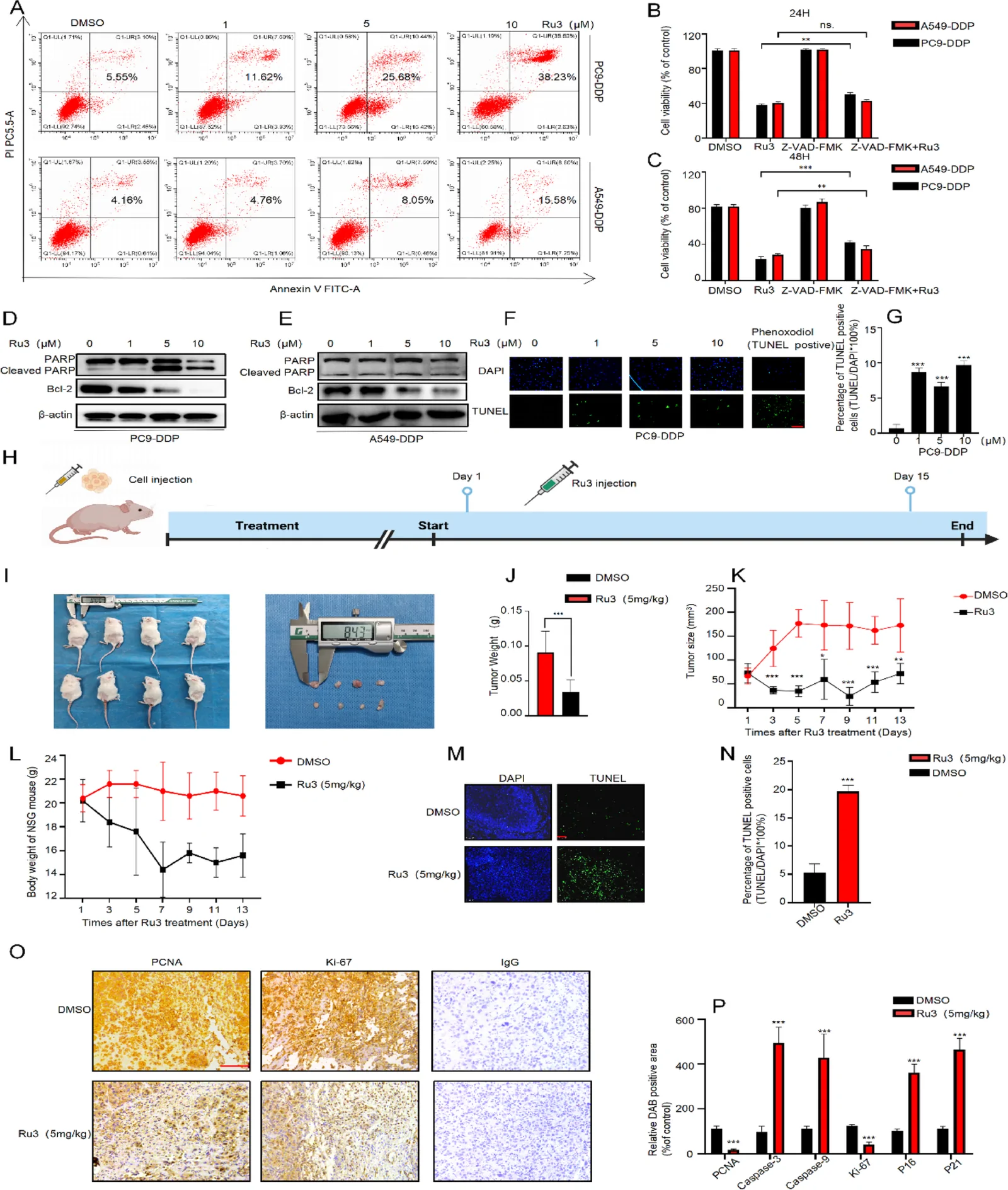
Ru3 induce apoptosis in cisplatin-resistant lung cancer cells both in vitro and in vivo **A** A549-DDP and PC9-DDP cells were treated with 5 μM Ru3 for 24 h, then fixed, stained with Annexin V/PI, and analyzed by flow cytometry to quantify the apoptosis rate. **B**–**C** The cell viability of A549-DDP and PC9-DDP cells treated with 5 μM Ru3 in the presence or absence of 10 μM Z-VAD-FMK for 24 or 48 h was detected by CCK-8 assays. **D**–**E** Immunoblot analysis was performed to detect PARP and Bcl-2 in A549-DDP and PC9-DDP cells treated with the indicated concentrations of Ru3 for 24 h. **F**–**G** TUNEL assay was conducted on PC9-DDP cells treated with 5 μM Ru3 for 24 h, with a scale bar of 250 μm. **H** Schematic diagram of the in vivo experimental procedure. **I**–**K** PC9-DDP cells were subcutaneously injected into NSG mice (n=4). Following tumor establishment, mice were administered either DMSO or Ru3 (5 mg/kg) via tail vein injection once daily. **J** The weight of the tumors was measured at the time of sacrifice. **K** Tumor volumes were monitored at the indicated time points. **L** The body weight of the mice was monitored at the indicated time points. **M**–**N** TUNEL apoptosis staining analysis was performed on mouse tumor tissue sections. **O**–**P** Representative images of immunohistochemical staining for PCNA and Ki-67 with quantification of the relative intensity of staining for these markers in xenografts. Scale bar: 100 μm. *, *P* < 0.05; **, *P* < 0.01; ***, *P* < 0.001. Statistical significance was compared with the respective control groups

Given the pivotal role of apoptosis induction in cancer chemotherapy, our initial research efforts were directed towards assessing the capacity of Ru3 to elicit apoptotic responses in cisplatin-resistant lung cancer cell lines. Employing Annexin V/PI staining coupled with flow cytometry analysis, we observed a marked elevation in the fraction of apoptotic cells following Ru3 treatment (Fig. 3A). To further validate this finding, we conducted a co-treatment experiment where Ru3 was administered in conjunction with Z-VAD-FMK, a potent caspase inhibitor known to block the apoptotic process. Subsequent CCK-8 assays revealed that co-treatment with Z-VAD-FMK partially rescued cell proliferation in a time-dependent manner, confirming the pro-apoptotic activity of Ru3 (Fig. 3B–C). To corroborate these observations at the molecular level, Western blot analysis was performed to examine the expression of PARP, a hallmark protein indicative of apoptosis. Our results demonstrated a concentration-dependent increase in the levels of Cleaved-PARP, accompanied by a notable decrease in the expression of the anti-apoptotic factor Bcl-2 (Fig. 3D–E). Additionally, TUNEL apoptosis staining was performed, and a marked increase in apoptosis-positive signals was observed in Ru3-treated PC9-DDP cells relative to the control group (Fig. 3F–G). Notably, Ru3 exhibited a weak pro-apoptotic effect in the normal human lung epithelial cell line NL20 (Figure S1R).

Furthermore, we systematically investigated the involvement of other cell death pathways in Ru3-mediated antitumor activity. Pharmacological inhibition of necrosis, pyroptosis, and endoplasmic reticulum (ER) stress with their respective specific inhibitors failed to reverse the cytotoxic effects of Ru3. These results indicated that necrosis, pyroptosis, and ER stress are not involved in the molecular mechanism underlying Ru3-induced tumor cell death (Figures S2A–B).

Encouraged by these in vitro findings, we extended our investigation to evaluate the anti-tumor efficacy of Ru3 in vivo. A mouse xenograft model was established by subcutaneously inoculating human PC9-DDP cells into NSG mice. Consistent with our in vitro data, Ru3 exhibited a substantial inhibitory effect on tumor proliferation, albeit accompanied by mild weight loss in the NSG mice (Fig. 3H–L). Tumor tissue sections subjected to TUNEL staining displayed a significant increase in TUNEL-positive signals in Ru3-treated mice compared to controls (Fig. 3M–N). Immunohistochemical (IHC) staining of tumor tissue sections further substantiated our in vitro findings (Fig. 3O–P and Figure S1C). To assess potential toxicity, organs were extracted from the mice and subjected to hematoxylin and eosin (HE) staining, which indicated mild pathological alterations in Ru3-treated mice compared to controls, suggesting an acceptable toxicity profile (Figure S1D). Collectively, these results provide compelling evidence that Ru3 exerts profound anti-tumor efficacy through the induction of apoptosis in cisplatin-resistant lung cancer cells, both in vitro and in vivo.

### IGFBP3 acts as a key downstream effector in the anti-cisplatin-resistant lung cancer effects of Ru3

**Fig. 4:**
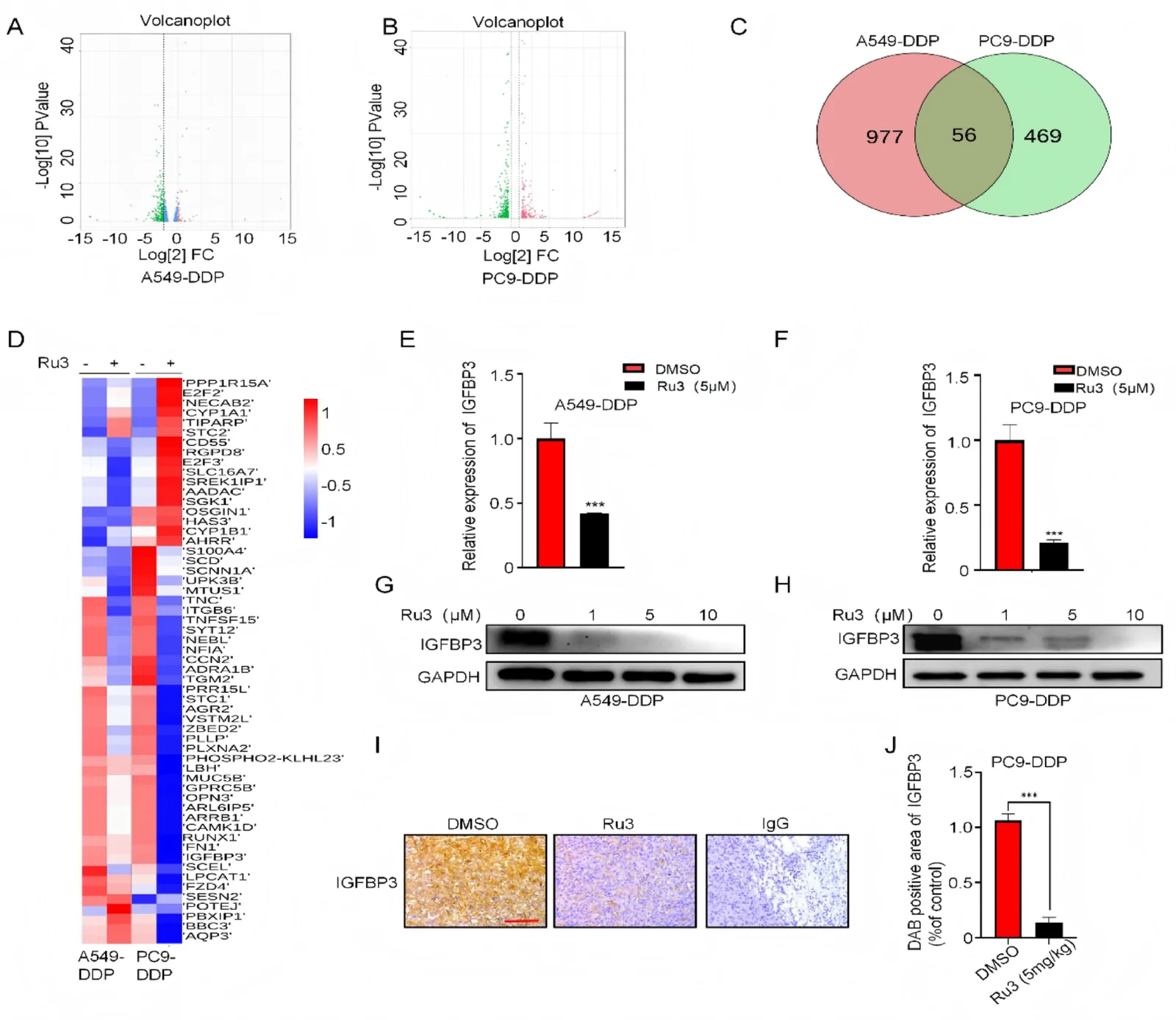
Ru3 inhibit IGFBP3 expression in the cisplatin-resistant lung cancer cells **A**–**B** Volcano plot analysis of differentially expressed genes (DEGs) in A549-DDP and PC9-DDP cells treated with or without 5 μM Ru3 for 24 h. The red areas indicate genes with significant overexpression, while the green areas indicate genes with significant downregulation. **C** A Venn diagram analysis was conducted to compare the gene expression profiles of PC9-DDP and A549-DDP cells after treatment with 5 μM Ru3. **D** A heatmap was generated to visualize the differentially expressed genes (DEGs) in PC9-DDP and A549-DDP cells following treatment with 5 μM Ru3. **E**–**F** RT-PCR analysis was performed to detect the expression of IGFBP3 in A549-DDP and PC9-DDP cells after 24 h of treatment with 5 μM Ru3. **G**–**H** Western blot analysis was conducted to assess the protein levels of IGFBP3 in A549-DDP and PC9-DDP cells after 24 h of treatment with 5 μM Ru3. **I**–**J** Representative images of immunohistochemical staining for IGFBP3 in xenografts, with quantification of the relative staining intensity. Scale bar: 100 μm. ***, *P* < 0.001. Statistical significance was determined by comparison with the respective control groups

To gain deeper insights into the molecular mechanisms underlying Ru3's robust antitumor effects, we conducted an RNA-seq analysis. Specifically, we observed 401 upregulated and 576 downregulated genes in A549-DDP cells, and 201 upregulated and 324 downregulated genes in PC9-DDP cells (Figs. 4A-B). Notably, 57 genes were found to be commonly dysregulated in both cell lines, suggesting a shared molecular signature underlying Ru3's activity (Fig. 4C). An enrichment analysis of these DEGs revealed that genes associated with the AKT-mTOR and P53 signaling pathways were most significantly enriched following Ru3 treatment (Figure S3A). This was further demonstrated by Western blot and transmission electron microscopy analyses, which revealed upregulation of P53-related genes, inactivation of the AKT/mTOR signaling cascade and activation of its downstream autophagy pathway (Figures S1P-Q and Figures S3B-E).

We next investigated whether ferroptosis contributes to Ru3-induced cytotoxicity. Using the Fe^2^⁺-specific probe RhoNox-6, we observed a partial increase in intracellular ferrous iron levels upon Ru3 treatment (Figures S3F–G), accompanied by a modest elevation in malondialdehyde (MDA) levels (Figure S3H) and increased lipid peroxidation, as detected by the LPO-specific probe BODIPY™ C11 (Figures S3I–J). In addition, glutathione (GSH) levels were significantly depleted following Ru3 exposure (Figure S3K). However, co-treatment with ferrostatin-1 (Fer-1), a specific ferroptosis inhibitor, failed to reverse the antitumor activity or alleviate the cytotoxicity of Ru3 (Figures S3L–M). Collectively, these results indicate that although Ru3 partially induces ferroptosis, this process does not serve as the primary pathway mediating its anticancer effects.

Among these shared DEGs, Insulin-like Growth Factor Binding Protein 3 (IGFBP3) emerged as a particularly intriguing candidate due to its marked suppression following Ru3 treatment (Fig. 4D) and pivotal role in regulating AKT-mTOR and P53 signaling pathways [18]. In addition, our previous research had already hinted at IGFBP3's influence on the proliferation of non-small cell lung cancer [29, 30]. To validate this observation, we conducted both RT-PCR and Western blot analyses, which consistently demonstrated that Ru3 significantly inhibits IGFBP3 expression in cisplatin-resistant lung cancer cells (Fig. 4E–H). Additionally, immunohistochemical staining of tumor tissues from a mouse xenograft model revealed a notable reduction in IGFBP3 levels in tumors treated with Ru3 compared to controls (Fig. 4I–J).

**Fig. 5:**
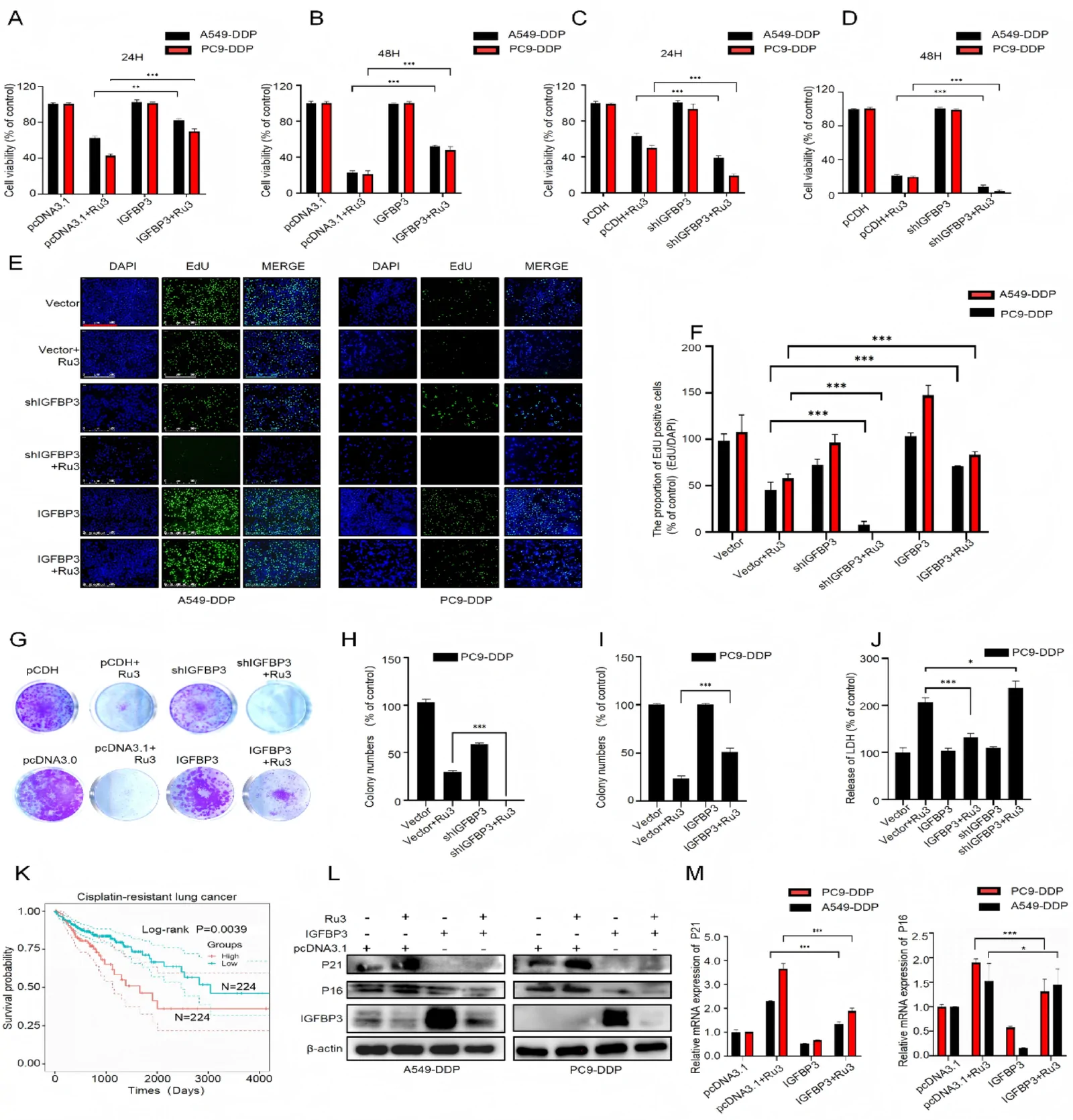
IGFBP3 acts as a key downstream effector in the anti- cisplatin-resistant lung cancer effects of Ru3 **A**–**B** CCK-8 assays were performed on A549-DDP and PC9-DDP cells overexpressing IGFBP3 or pcDNA3.1, treated with 5 μM Ru3 for 24 or 48 h. **C**–**D** CCK-8 assays were conducted on A549-DDP and PC9-DDP cells stably expressing shIGFBP3 or pCDH via lentiviral infection, treated with 5 μM Ru3 for 24 or 48 h. **E**–**F** EdU assays were performed on A549-DDP and PC9-DDP cells overexpressing IGFBP3 or with IGFBP3 knockdown, treated with 5 μM Ru3 for 24 h. Scale bar: 250 μm. **G**–**I** Clone formation assays were conducted on PC9-DDP cells overexpressing IGFBP3 or with IGFBP3 knockdown, treated with indicated concentrations of Ru3 for 24 h. **J** Lactate dehydrogenase release was analyzed in PC9-DDP cells overexpressing IGFBP3 or with IGFBP3-knockdown, treated with indicated with 5 μM Ru3 for 24 h; cell lysates served as the positive control. **K** A Kaplan–Meier curves of TCGA cisplatin-resistant lung cancer stratified by IGFBP3 expression (low vs high). **L** Immunoblot analysis detected P16, P21, and IGFBP3 in A549-DDP and PC9-DDP cells overexpressing IGFBP3 or pcDNA3.1, after treatment with 5 μM Ru3 for 24 h. **M** RT-PCR analysis was used to detect P16 and P21 in A549-DDP and PC9-DDP cells overexpressing IGFBP3 or pcDNA3.1, after treatment with 5 μM Ru3 for 24 h. Data are presented as means ± s.d. from three independent experiments. *, *P* < 0.05; **, *P* < 0.01; ***, *P* < 0.001, compared with the respective control groups

To delve deeper into the intricacies of IGFBP3's function and its interplay with Ru3, we leveraged IGFBP3 overexpression plasmids to establish an IGFBP3-overexpressing cisplatin-resistant lung cancer cell line, thereby paving the way for subsequent experimental explorations (Figures S3N-O). Our subsequent CCK-8 assays revealed that overexpression of IGFBP3 partially rescued the antiproliferative effects of Ru3 (Fig. 5A–B). Conversely, knockdown of IGFBP3 using a lentiviral vector enhanced the sensitivity of these cells to Ru3 (Fig. 5C–D and Figures S4A-C), further corroborating IGFBP3's role in modulating the cellular response to Ru3. These findings were further substantiated by EdU cell proliferation assays, plate clone formation experiments, and LDH release assays, which consistently demonstrated that IGFBP3 overexpression attenuated Ru3's antitumor effects (Fig. 5E–J). Simultaneously, our analysis in the TCGA database revealed that among lung cancer patients resistant to cisplatin, those with high expression levels of IGFBP3 have a poorer prognosis (Fig. 5K). Moreover, our investigation into the molecular mechanisms revealed that IGFBP3 overexpression also impacts cell cycle regulation. RT-PCR and Western blot analyses showed decreased expression of cell cycle inhibitory proteins P21 and P16 in IGFBP3-overexpressing cells compared to controls (Fig. 5L–M). Additionally, the expression levels of IL-6 and IL-8, cytokines indicative of cell cycle arrest and cellular senescence, were significantly potentiated by Ru3 but attenuated in IGFBP3-overexpressing cells (Figures S4D-E). Collectively, our data support the notion that IGFBP3 functions as a pivotal downstream effector of Ru3's activity, playing a crucial role in modulating the response of cisplatin-resistant lung cancer cells to this novel ruthenium-based chemotherapeutic agent.

### Ru3 attenuated the lethal ROS scavenging capacity of cisplatin-resistant lung cancer cells via inhibition of the IGFBP3-GPX4 axis

**Fig. 6:**
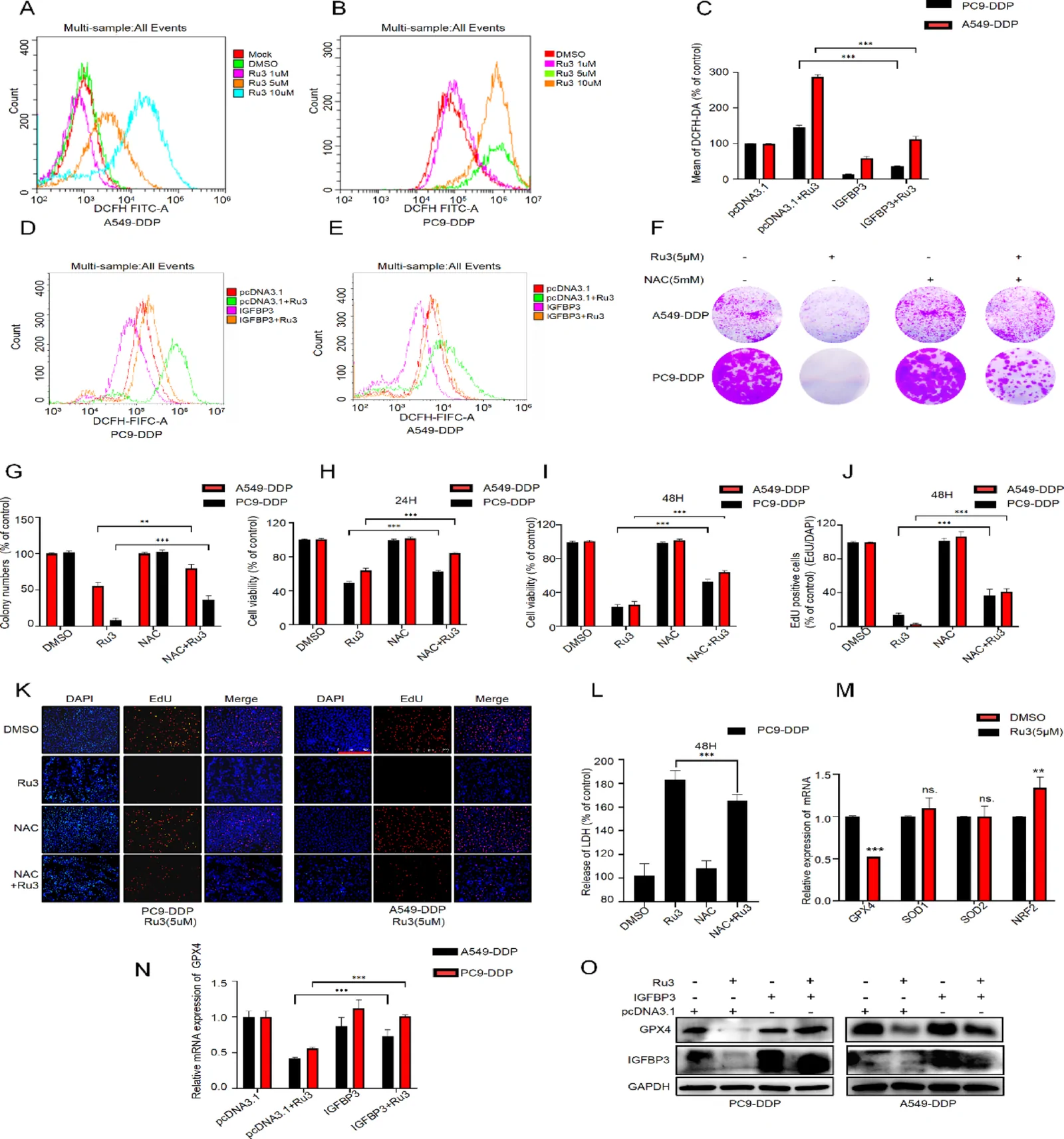
Ru3 attenuated the lethal ROS scavenging capacity of cisplatin-resistant lung cancer cells via inhibition of the IGFBP3-GPX4 axis **A**–**B** ROS levels were determined by flow cytometry in A549-DDP and PC9-DDP cells treated with indicated concentrations of Ru3 for 24 h. **C**–**E** ROS levels were determined by flow cytometry in A549-DDP and PC9-DDP cells overexpressing IGFBP3 or pcDNA3.1, after treatment with 5 μM Ru3 for 24 h. **F**–**G** Clone formation assays were conducted on A549-DDP and PC9-DDP cells treated with 5 μM Ru3 in the presence or absence of 5 mM NAC. **H**–**I** The cell viability of A549-DDP and PC9-DDP cells treated with 5 μM Ru3 in the presence or absence of 5 mM NAC for 24 h or 48 h was detected by CCK-8 assays. **J**–**K** EdU assays were conducted on A549-DDP and PC9-DDP cells treated with 5 μM Ru3 in the presence or absence of 5 mM NAC for 48 h. **L** Lactate dehydrogenase release was analyzed in PC9-DDP cells treated with 5 μM Ru3 in the presence or absence of 5 mM NAC. (M) qRT-PCR analysis was performed to detect SOD1, SOD2, GPX4, and NRF2 in PC9-DDP cells after treatment with 5 μM Ru3 for 24 h. **N** qRT-PCR analysis was performed to detect GPX4 in A549-DDP and PC9-DDP cells overexpressing IGFBP3 or pcDNA3.1, after treatment with 5 μM Ru3 for 24 h. **O** Western-Blot analysis was performed to detect GPX4 and IGFBP3 in A549-DDP and PC9-DDP cells overexpressing IGFBP3 or pcDNA3.1, after treatment with 5 μM Ru3 for 24 h. Data are representative of three independent experiments and are presented as means ± s.d. *, *P* < 0.05; **, *P* < 0.01; ***, *P* < 0.001. Statistical significance was determined by comparing with the respective control groups

Ruthenium complexes were found to induce lethal ROS accumulation in cancer cells [11], while IGFBP3 plays a pivotal role in regulating ROS dynamics within tumors [31]. These findings prompted us to hypothesize that the antitumor activity of Ru3 might also involve modulation of ROS levels. To test this hypothesis, we employed the DCFH-DA ROS probe for intracellular staining followed by flow cytometric analysis. A direct correlation was observed between ROS accumulation following Ru3 treatment and drug concentration (Fig. 6A–B). Intriguingly, when IGFBP3 was overexpressed in these cells, the Ru3-induced ROS accumulation was substantially mitigated (Fig. 6C–E). These data strongly suggest that Ru3 significantly induces ROS accumulation in cisplatin-resistant lung cancer cells, an effect that can be substantially mitigated by IGFBP3 overexpression. To explore the functional implications of ROS on tumor cell proliferation, we treated tumor cells with a combination of Ru3 and the ROS scavenger N-acetylcysteine (NAC). Subsequently, the cells were assessed using CCK-8, EdU incorporation, and clone formation assays. Our findings revealed that compared to cells treated solely with Ru3, those co-treated with Ru3 and NAC exhibited an augmentation of the proliferative capacity of the tumor cells (Fig. 6F–K). Notably, the co-treatment group showed a reduction in LDH release compared to the controls (Fig. 6L). Collectively, these findings imply that ROS accumulation induced by Ru3 serves as an inhibitory mechanism against the proliferation of cisplatin-resistant lung cancer cells.

To delve deeper into the mechanism underlying IGFBP3-mediated ROS regulation, we scrutinized our RNA-seq data and selected several antioxidant-related genes for further analysis via qRT-PCR. Among these genes, GPX4 was found to be the most profoundly inhibited following Ru3 treatment (Fig. 6M). Subsequent qRT-PCR and western blot analyses confirmed that overexpression of IGFBP3 partially restored GPX4 levels that had been suppressed by Ru3 (Fig. 6N–O). Meanwhile, we examined the expression of GPX4 and IGFBP3 in both parental and DDP-resistant lung cancer cells. As a result, both GPX4 and IGFBP3 were found to have higher expression levels in cisplatin-resistant lung cancer (Figures S4F-G).

In conclusion, our study has unveiled a novel mechanism by which Ru3 disrupts the ROS homeostasis of tumors by inhibiting the IGFBP3-GPX4 axis. This ROS accumulation constitutes a pivotal pathway through which Ru3 exerts its antitumor effects, ultimately leading to the elimination of tumor cells.

### Ru3 promoted ROS accumulation by inducing mitochondrial damage in cisplatin-resistant lung cancer cells

**Fig. 7:**
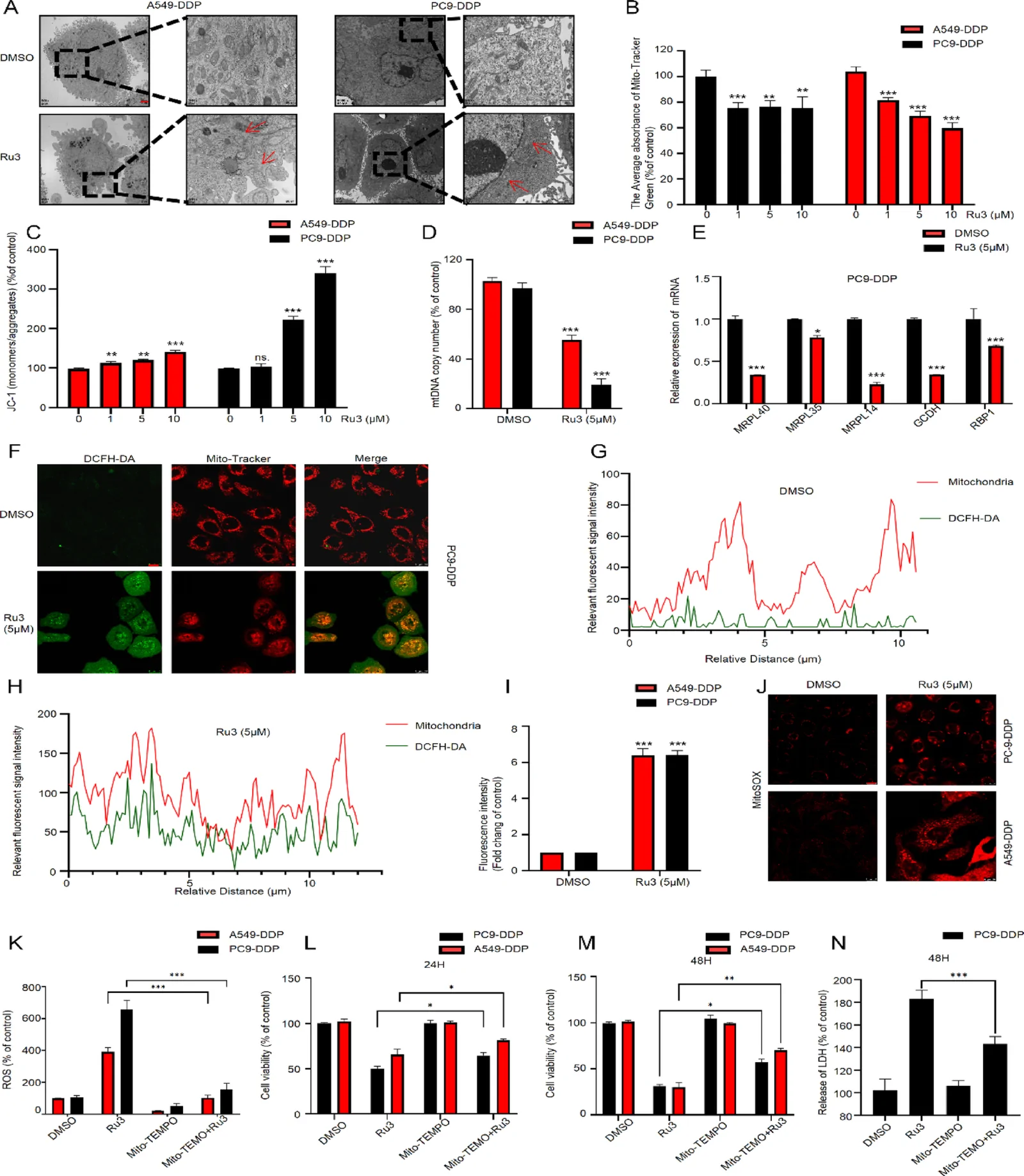
Ru3 promoted ROS accumulation by inducing mitochondrial damage in cisplatin-resistant lung cancer cells **A** Mitochondrial morphology was captured in cells with and without the addition of 5 μM Ru3 using transmission electron microscopy. Scale bar, 2 μm. **B** Mitotracker-Green was utilized to assess mitochondrial mass, independent of mitochondrial membrane potential (MMP) alterations, in A549-DDP and PC9-DDP cells treated with or without 5 μM Ru3 for 24 h. The analysis was conducted using a fluorescence microplate reader (Thermo Scientific). **C** JC-1 analysis was performed to assess mitochondrial membrane potential changes in A549-DDP and PC9-DDP cells in the presence or absence of 5 μM Ru3. **D** The number of mitochondrial DNA copies was examined by qRT-PCR in A549-DDP and PC9-DDP cells treated with or without 5 μM Ru3 for 24 h. **E** qRT-PCR analysis was performed to detect MRPL40, MRPL35, MRPL14, GCDH, and RBP1 in PC9-DDP cells after treatment with 5 μM Ru3 for 24 h. **F**–**H** The colocalization of Mitotracker Red and DCFH-DA was analyzed in PC9-DDP cells both with and without the treatment of 5 μM Ru3. Scale bar, 10 μm. **I**–**J** MitoSOX Red was used to evaluate oxide levels in mitochondria of A549-DDP and PC9-DDP cells upon treatment with 5 μM Ru3. Scale bar, 10 μm. **K** ROS levels were determined by flow cytometry in A549-DDP and PC9-DDP cells treated with 5 μM Ru3 in the presence or absence of 10 μM Mito-TEMPO for 24 h. **L**–**M** The cell viability of A549-DDP and PC9-DDP cells treated with 5 μM Ru3 in the presence or absence of 10 μM Mito-TEMPO for 24 h or 48 h was detected by CCK-8 assays. (N) Lactate dehydrogenase release was analyzed in PC9-DDP cells treated with 5 μM Ru3 in the presence or absence of 10 μM Mito-TEMPO for 48 h. Data are representative of three independent experiments and are presented as means ± s.d. *, *P* < 0.05; **, *P* < 0.01; ***, *P* < 0.001. Statistical significance was determined by comparing with the respective control groups

In transmission electron microscopy (TEM) assays for detecting autophagosomes, we also observed pronounced mitochondrial damage, characterized by swollen, enlarged, and rounded mitochondria with lighter or translucent matrices, absent matrix granules, and shortened cristae, in contrast to the control group (Fig. 7A). This mitochondrial damage was further supported by quantitative analysis of mitochondrial mass, which decreased upon Ru3 treatment (Fig. 7B). Additionally, Ru3 treatment led to a significant increase in the fluorescence intensity of JC-1 monomers and a concurrent decrease in JC-1 aggregates, indicating mitochondrial depolarization (Fig. 7C). Consistently, the mitochondrial DNA (mtDNA) copy number, a proxy for mitochondrial abundance, was also reduced upon Ru3 treatment (Fig. 7D). Furthermore, after cytosolic and mitochondrial fractionation, we detected the protein levels of TOMM20 (translocase of outer mitochondrial membrane 20), a marker for mitochondrial content. Both the total TOMM20 and mitochondrial TOMM20 were decreased following Ru3 treatment (Figure S5A), further confirming the occurrence of severe mitochondrial damage in Ru3-treated cells. In addition, the mRNA levels of several key oxidative phosphorylation-related proteins were suppressed by Ru3 (Fig. 7E). These observations collectively confirm that Ru3 directly damages mitochondria in cisplatin-resistant lung cancer cells.

Mitochondria are a major site for the production of ROS. To determine if mitochondrial damage was the cause of Ru3-induced ROS accumulation, we performed co-localization staining using ROS and mitochondrial probes. Our results showed that Ru3-treated tumor cells exhibited markedly abnormal mitochondrial morphology and a pronounced increase in ROS signals, which were distinctly co-localized with mitochondria (Fig. 7F–H and Figures S5B-C). Furthermore, using MitoSOX, a specific fluorescent indicator for mitochondrial superoxide, we confirmed that Ru3 treatment significantly elevated mitochondrial superoxide levels (Fig. 7I–J). To assess the functional consequences of mitochondrial ROS generation, we treated tumor cells with a combination of Ru3 and the mitochondrial superoxide scavenger Mito-TEMPO. Our results revealed that Mito-TEMPO completely reversed Ru3-induced ROS accumulation and significantly attenuated Ru3's inhibitory effects on proliferation and cytotoxicity (Figs. 7K–N and Figures S5D-F). Interestingly, we found that the level of ROS did not affect the expression of IGFBP3 (Figures S5G-H).

In conclusion, our findings clearly demonstrate that ROS accumulation observed during Ru3 chemotherapy primarily arises from mitochondrial damage. Collectively, the attenuation of mitochondrial superoxide significantly diminished Ru3's inhibitory effects on tumor cell proliferation and cytotoxicity, underscoring the critical role of mitochondrial damage in mediating Ru3's antitumor activity.

### Ru3 inhibited IGFBP3 transcription by enhancing E2F2 expression

**Fig. 8:**
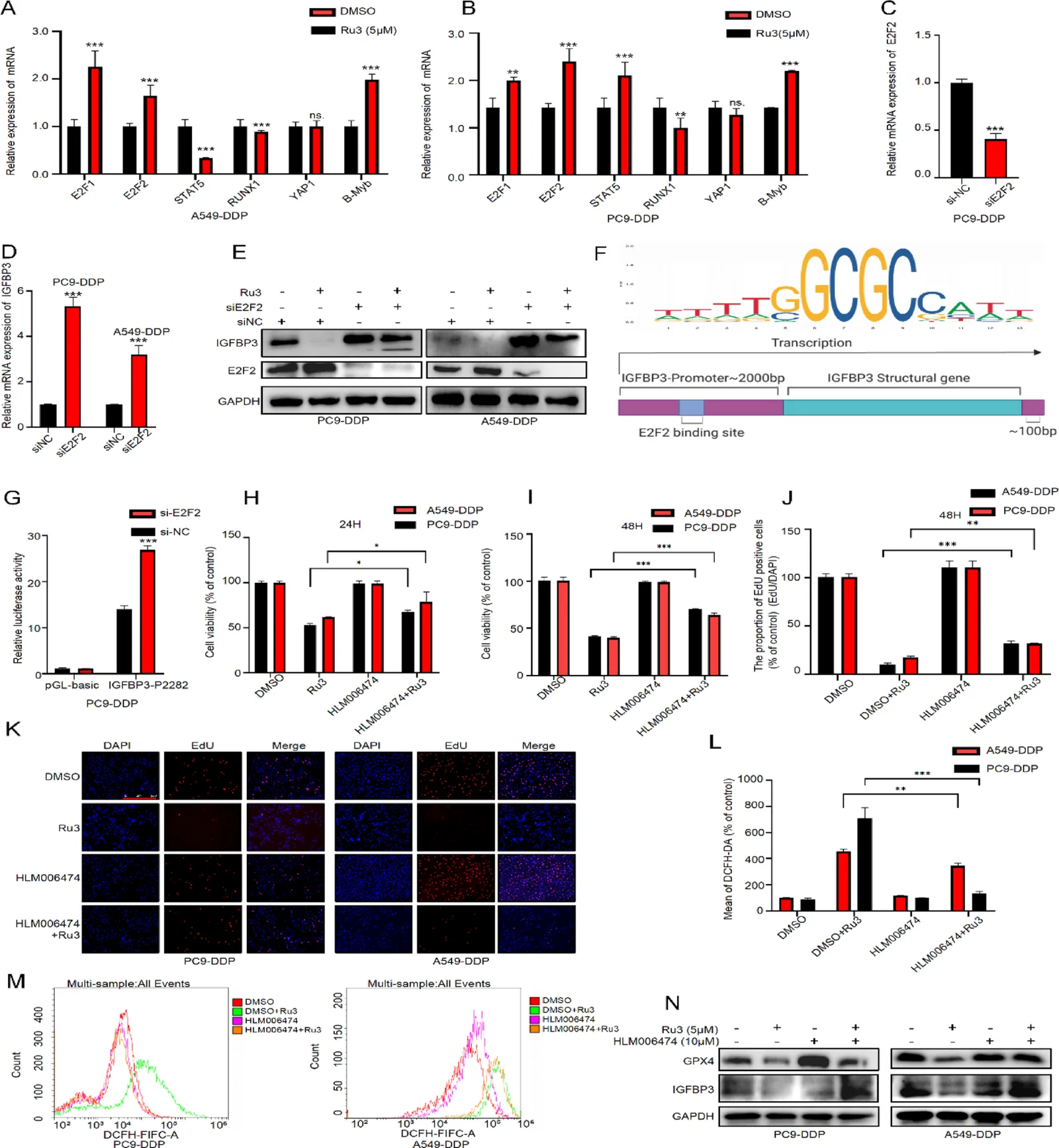
Ru3 inhibited IGFBP3 transcription by enhancing E2F2 expression **A**–**B** qRT-PCR analysis was performed to detect the expression of E2F1, E2F2, STAT5, RUNX1, and YAP1 in PC9-DDP and A549-DDP cells after treatment with 5 μM Ru3 for 24 h. **C** qRT-PCR was conducted to measure E2F2 expression in PC9-DDP cells following treatment with siE2F2. **D** The expression of IGFBP3 was assessed by qRT-PCR in A549-DDP and PC9-DDP cells after siE2F2 treatment. **E** Immunoblot analysis was used to detect E2F2 and IGFBP3 protein levels in A549-DDP and PC9-DDP cells treated with 5 μM Ru3, either in the presence or absence of siE2F2, for 24 h. **F** Schematic diagram of the transcription start site (TSS) region of the IGFBP3 gene locus. Illustration of the E2F2-binding motif and its position within the TSS. Data sourced from the JASPAR database. **G** Dual-luciferase reporter assays were conducted in PC9-DDP cells transfected with siE2F2 or siNC. **H**–**I** The cell viability of A549-DDP and PC9-DDP cells, treated with 5 μM Ru3 in combination with or without 10 μM HLM006474 for 24 or 48 h, was assessed using CCK-8 assays. **J**–**K** EdU assays were performed on A549-DDP and PC9-DDP cells after 48 h of treatment with 5 μM Ru3, either alone or in combination with 10 μM HLM006474. Scale bar: 250 μm. **L**–**M** ROS levels were measured by flow cytometry in A549-DDP and PC9-DDP cells following 24 h of treatment with 5 μM Ru3, in combination with or without 10 μM HLM006474. **N** Immunoblot analysis was conducted to detect GPX4 and IGFBP3 protein levels in A549-DDP and PC9-DDP cells after 24 h of treatment with 5 μM Ru3, in the presence or absence of 10 μM HLM006474. Data are presented as means ± s.d. and are representative of three independent experiments. *, *P* < 0.05; **, *P* < 0.01; ***, *P* < 0.001. Statistical significance was compared with the respective control groups

To gain a deeper understanding of the mechanisms responsible for Ru3's potent inhibition of IGFBP3, we focused our attention on the potential transcriptional pathways involved. Guided by our sequencing data, we initiated a systematic search for transcription factors that exhibited alterations in their expression following Ru3 treatment. These findings were subsequently validated through qRT-PCR analysis (Fig. 8A–B). Among the transcription factors identified, B-Myb, E2F1 and E2F2, have previously been reported to regulate IGFBP3 expression by our and other’s prior research [23, 29, 32]. To validate this relationship, we firstly employed Western blot analysis to assess changes in B-Myb expression upon Ru3 treatment. Consistent with our sequencing and PCR results, Ru3 was found to induce a concentration-dependent increase in B-Myb protein levels (Figures S6A-B). Furthermore, knock down of B-Myb would attenuate the anticancer capacity of Ru3 and inhibit the expression of IGFBP3, suggesting Ru3-induced IGFBP3 transcription regulation is independent of B-Myb (Figures S6 C-H). E2F1 exerted only a marginal influence on IGFBP3 expression (Figures S6I-J), and only knockdown of E2F2 partially restored IGFBP3 expression levels (Figs. 8C–E). To ascertain whether E2F2 regulates the transcription of IGFBP3, we initially identified the transcription factor binding sites (TFBS) of E2F2 in IGFBP3 promoter in the JASPAR database (Fig. 8F) [33]. Following this, we searched the NCBI database for the IGFBP3 gene's promoter sequence, we discovered several potential E2F2 binding sites scattered within the region of the IGFBP3 promoter (Figure S7A). This finding hinted that E2F2 could potentially bind directly to the IGFBP3 promoter to modulate transcription. To test this hypothesis, we cloned a 2282-bp fragment of the IGFBP3 promoter into the pGL-Basic plasmid and conducted a dual-luciferase reporter gene assay (Figure S7B). The results were consistent with our hypothesis, we observed a notable increase in luciferase activity driven by the IGFBP3 promoter when siE2F2 was present, suggesting that E2F2 directly binds to the IGFBP3 promoter to regulate its transcription (Fig. 8G).

To further substantiate the role of the E2F2/IGFBP3/GPX4 axis in mediating the antitumor effects of Ru3, we performed a series of functional assays following inhibition of E2F2 using the pan‑E2F inhibitor HLM006474. HLM006474 significantly reversed the transcription repression of Ru3 on IGFBP3 (Figure S7C). CCK-8 and EdU experiments revealed that inhibiting E2F2 partially restored the proliferative capacity of tumor cells treated with Ru3 (Fig. 8H–K and Figures S7D-E). Concurrently, flow cytometry analysis showed that inhibiting E2F2 significantly reduced ROS levels in Ru3-treated cells (Fig. 8L–M). Furthermore, Western blot analysis demonstrated that inhibition of E2F2 partially restored the expression of GPX4 (Fig. 8N). These results collectively suggest that Ru3's upregulation of E2F2 leads to the suppression of IGFBP3, which weakens tumor antioxidant capacity, facilitates ROS accumulation, and finally eliminates tumor cells.. Furthermore, we employed siRNA to specifically knock down GPX4 (Figure S7F) and through a series of proliferation related assays, found that GPX4 depletion significantly enhanced the antitumor activity of Ru3 (Figures S7G–K). Moreover, co treatment with the GPX4-targeting inhibitor RLS3 and Ru3 yielded similar results (Figures S7L–O). Collectively, our experimental data demonstrate that the E2F2/IGFBP3/GPX4 axis constitutes the core pathway mediating the antitumor effects of Ru3.

## Discussion

Cisplatin, a platinum-based chemotherapeutic agent, has long been a cornerstone in the treatment of lung cancer, particularly in its ability to induce DNA cross-links, thereby impeding cellular replication and transcription [34]. However, the emergence of cisplatin resistance among tumor cells poses a significant challenge, as it compromises the efficacy of this drug [35, 36]. This resistance can arise through various mechanisms, including reduced drug uptake, enhanced drug efflux, and heightened DNA repair capacity [37]. Consequently, there is an urgent need for alternative therapeutic agents capable of overcoming this resistance. Metal ruthenium compounds have garnered considerable attention in recent years due to their potential as novel antitumor agents [38]. Unlike cisplatin, which primarily targets DNA, ruthenium-based drugs exhibit a more diverse array of mechanisms, including interference with intracellular signaling pathways, modulation of protein function, and induction of oxidative stress [38, 11]. These distinct mechanisms not only broaden the therapeutic window but also reduce the likelihood of resistance development [11]. Additionally, ruthenium compounds often exhibit favorable pharmacokinetic profiles, leading to higher accumulation and prolonged retention within tumor tissues, thereby enhancing their antitumor efficacy [39]. Finally, their distinct mechanisms of action may result in lower toxicity and fewer side effects compared to conventional agents [40]. Given these benefits, ruthenium-based drugs represent a valuable addition to the anticancer arsenal, particularly for patients who have developed resistance to conventional therapies. In this context, our research aimed to identify and characterize a novel ruthenium-based compound, designated Ru3, with potential therapeutic efficacy against cisplatin-resistant lung cancer. In this study, we uncover a novel mechanism where Ru3 exerts its antitumor effect by upregulating E2F2, which directly binds to the IGFBP3 promoter to regulate transcription. This triggers a cascade of events that ultimately lead to the accumulation of ROS and tumor cell death.

ROS have increasingly garnered attention in the realm of cancer therapy over recent years [15]. When the levels of ROS exceed the cellular antioxidant defenses, oxidative stress ensues, resulting in lipid peroxidation of cell membranes, protein damage, and DNA mutations [15]. This sequence of events ultimately culminates in the induction of apoptosis or necrosis in the targeted cells [15]. Consequently, the strategic utilization of drugs or alternative therapeutic modalities to specifically elevate ROS levels in cancer cells has emerged as a highly promising therapeutic approach [16]. For example, in our prior research endeavors, we demonstrated that CPX (ciclopirox olamine) effectively promotes the accumulation of ROS across various cancer types, thereby significantly contributing to tumor eradication [41, 42]. More recently, ruthenium compounds have been identified as potent inducers of ROS accumulation in cancer cells [11]. In the present study, Ru3 was found to target mitochondria and markedly induce mitochondrial damage in cisplatin-resistant lung cancer cells. This mitochondrial dysfunction subsequently triggered a substantial accumulation of ROS within the cellular milieu, ultimately precipitating oxidative stress and ensuing cell death. However, this cannot explain why our new ruthenium-based chemotherapy agent shows powerful tumor-inhibiting effects on cisplatin-resistant lung cancer, yet has a relatively mild impact on the parent (non-resistant) lung cancer cells.

As an emerging challenge in cancer therapy, the development of cisplatin resistance in tumors is frequently linked to perturbations within the antioxidant system [17]. Under normal physiological conditions, tumors maintain a delicate equilibrium between the generation of ROS and their removal by enzymatic and non-enzymatic antioxidants [43, 44]. Catalase, alongside glutathione (GSH) and thioredoxin (TRX) systems, play pivotal roles in modulating ROS levels to prevent oxidative damage [43, 44]. Several studies have demonstrated that cisplatin targets thioredoxin reductase (TrxR), leading to an escalation of intracellular ROS, which ultimately culminates in growth arrest and cell death [17, 45, 46]. For instance, Witte et al. reported that anticancer alkylating agents and platinum-containing compounds, such as cisplatin and oxaliplatin, exhibit significant and irreversible inhibitory effects on TrxR [45]. To adapt and survive under heightened ROS levels and evade cisplatin-induced cell death, tumor cells often reduce their reliance on TRX and compensate by enhancing the activity of other antioxidant systems, notably the GSH system [17, 45, 46]. This shift is exemplified by the study of Rocha et al., where the glutathione inhibitor buthionine sulfoximine (BSO) significantly sensitized cisplatin-resistant malignant gliomas to cisplatin [47]. Similarly, Wangpaichitr et al. found that elesclomol selectively eliminated cisplatin-resistant cells with low TRX expression, while exerting minimal effects on normal and parental cells [48]. In our study, treatment with Ru3 resulted in significant cell death in cisplatin-resistant lung cancer cells, yet had a minimal impact on parental lung cancer cells. We hypothesize that this selectivity may be attributed to the suppression of TRX in the antioxidant system of cisplatin-resistant cells, coupled with an increase in the activity of the GSH antioxidant system. Importantly, Ru3 specifically inhibits GPX4, which is highly expressed in these resistant cells. Furthermore, our findings indicate that the level of GPX4 is regulated by IGFBP3, and that IGFBP3 is markedly suppressed by Ru3.

IGFBP3, a multifaceted protein within the insulin-like growth factor system, exhibits complex regulatory effects on ROS, playing a pivotal role in cancer therapy. Prior studies have demonstrated that IGFBP3 can enhance ionizing radiation (IR)-induced cell death in vitro by promoting ROS production, an effect that can be mitigated by the NF-κB inhibitor BMS-345541 [22]. Conversely, in esophageal cancer, IGFBP3 is upregulated in hypoxic tumor microenvironments and functions to suppress oxidative stress by inhibiting ROS in an IGF-independent manner, thereby promoting tumor growth [49]. In the context of cisplatin-resistant lung cancer cells, our research reveals a novel mechanism whereby IGFBP3 possesses a robust ability to scavenge ROS by upregulating GPX4 expression. In contrast, Ru3, a novel ruthenium-based chemotherapeutic agent, exerts its antitumor effects by specifically inhibiting IGFBP3 transcription. This inhibition disrupts the ROS scavenging capacity of cisplatin-resistant cells, leading to ROS accumulation and subsequent cell death. IGFBP3's role in cancer therapy is further underscored by its association with tumor chemoresistance [49]. Various studies have demonstrated that changes in IGFBP3 expression levels can significantly affect the cytotoxic effects of chemotherapeutic drugs on tumor cells [50]. Clinical investigations comparing cisplatin-sensitive and -insensitive tissues from oral squamous cell carcinoma (OSCC) patients have shown a positive correlation between IGFBP3 expression and cisplatin sensitivity [51]. Additionally, the phosphorylation of IGFBP3 by Casein Kinase 2 (CK2) has been implicated in increasing cisplatin resistance in NSCLC, suggesting blocking this phosphorylation event may represent a promising strategy to enhance NSCLC sensitivity to cisplatin [52]. Additionally, Leslie's team found that high IGFBP3 expression enhances DNA repair capacity in prostate cancer by activating the epidermal growth factor receptor (EGFR) and DNA-dependent protein kinase catalytic subunit (DNA-PKcs), thereby promoting resistance to the PARP inhibitor Olaparib [50]. These findings align with our research, which demonstrates that cisplatin-resistant lung cancer cells express higher levels of IGFBP3 compared to their parental counterparts (Figures S4F-G). Our study uncovers a unique mechanism by which Ru3 promotes antitumor activity in cisplatin-resistant lung cancer cells through the upregulation of E2F2, which directly binds to the IGFBP3 promoter to inhibit its transcription. This discovery not only provides insights into the molecular mechanisms underlying cisplatin resistance but also highlights the potential of ruthenium-based compounds as novel therapeutic agents for the treatment of drug-resistant lung cancer.

As a member of the E2F transcription factor family, E2F2 is crucial in regulating gene expression, particularly in the context of cell cycle control and DNA repair [25]. Despite its pivotal role, the intricate direct interplay between E2F2 and IGFBP3 has remained understudied. Previous research from our laboratory found that E2F2 interacts with B-Myb to inhibit FOXM1 transcription, ultimately promoting LUAD progression [53]. Similarly, in colorectal cancer, E2F2 interacts with B-Myb, leading to the inhibition of the ERK and AKT signaling pathways by directly binding to the IGFBP3 promoter to regulate its expression and thus suppressing tumor progression [54]. In this study, we unveil a novel finding that E2F2 can directly bind to the IGFBP3 promoter and inhibit its transcription. Typically, E2F2 is associated with a negative role in cancer treatment, as high E2F2 expression often correlates with shorter overall survival and chemotherapy resistance in various cancers [55, 56]. For instance, Kim et al. found that activation of E2F through Rb degradation and c-Myc drives resistance to CDK4/6 inhibitors in breast cancer [57]. Thus, inhibiting E2F family activity has been proposed as a promising therapeutic target for cancer. However, our current study demonstrates a tumor-suppressive effect of E2F2 by modulating the antioxidant system. Previous studies have reported E2F2's role in regulating the antioxidant system [26]. For example, in hepatocellular carcinoma, E2F2 binds to the BRCA1 promoter, regulating its transcription and subsequently modulating the NRF2 cascade to enhance the antioxidant response and reduce ROS levels, thereby protecting HCC cells from oxidative damage in hepatocellular carcinoma [26]. Notably, we first report on the regulation of GPX4 by E2F2. We found that inhibiting E2F2 promotes the expression of IGFBP3-GPX4, effectively scavenging ROS and leading to cisplatin resistance.

In conclusion, our research introduces a novel ruthenium-based chemotherapeutic agent, Ru3, which exhibits potent antitumor activity against cisplatin-resistant lung cancer cells. The mechanism underlying this efficacy involves the induction of mitochondrial damage, resulting in ROS accumulation and subsequent oxidative stress-induced cell death. The inhibition of IGFBP3 by Ru3, mediated through the upregulation of E2F2 by directly binding to the IGFBP3 promoter and regulates its transcription, further disrupts the GPX4 antioxidant defense system of these resistant cells, exacerbating ROS levels and promoting tumor cell elimination. While the exact molecular mechanisms underlying this selectivity remain to be fully elucidated, our findings provide valuable insights into the potential of ruthenium-based compounds as a novel therapeutic approach for cisplatin-resistant lung cancer. Future studies should aim to explore the broader applicability of Ru3 in different cancer types and to identify biomarkers that can predict patient responsiveness to this therapy, ultimately facilitating the development of personalized treatment strategies.

In future studies, we also aim to explore the antitumor potential of Ru3 in patient derived organoids from cisplatin resistant lung cancer. Furthermore, additional investigations are warranted to ensure the safety and feasibility of Ru3 as a novel anticancer agent for clinical application.

## Materials and methods

### Chemical synthesis

RuCl_3_·3H_2_O, ligands, and other reagents from Acros, Aldrich, and Strem were all performed in a nitrogen environment. Column chromatography was formed with silica gel (300 to 400 mesh). ^1^H and ^13^C NMR spectra were recorded at ambient temperature using a Bruker Advance (400 MHz). NMR standards were as follows: (^1^H NMR) Acetonitrile-d_3_=1.94 ppm, DMSO-d_6_=2.50 ppm, (^13^C NMR) Acetonitrile-d_3_=1.32 ppm, DMSO-d_6_=39.52 ppm. ^1^H NMR data are reported as follows: chemical shifts of tetramethylsilane in the ppm front field (δ-scale), multiplicity (s=single peak, d=double peak, t=triple peak, q=quartet peak, m=multiple peak, br=broad peak), coupling constants (Hz) and integration. ^13^C NMR chemical shifts are reported in ppm downfield from tetramethylsilane (δ-scale). All compounds were analyzed and purified using RP-HPLC (Shimadzu LC-20AD equipped with an SPD-20A UV detector [258 and 287 nm]) and an Ultimate AQ-C18 (5 µm, 4.6 mm×250 mm) column at 0.5 mL/min at 35 ℃. The mobile phase consisted of an aqueous fraction (0.05% TFA in H_2_O) and an organic fraction (MeCN). Purity of all the twelve compounds is >95%. High resolution mass spectra were recorded on a Micromass AutoSpec instrument using ESI technique.

### Syntheses and characterization

The synthesis process of **Ru-1~5**: the ruthenium (II) dimer complex [(*η*^*6*^*-p*-cymene)Ru(μ-Cl)Cl]_2_ (0.04 mmol) and ligand NN (0.08 mmol) were dissolved in 15 mL chloroform/absolute methanol (1:1, v/v) solution and purged with N_2_ for 15 min, then heated to 40 ℃ and stirred for 4 h. The resulting yellow mixture was cooled to room temperature and steamed to a solid, it was then transferred to a centrifuge tube containing 15 mL of water and mixed thoroughly. The crude product was then precipitated by addition of excess saturated ammonium hexafluorophosphate (NH_4_PF_6_), purified by silica gel column chromatography, and eluted with MeCN/H_2_O/KNO_3_ solution. The elute of the combined product was dried and dissolved in 10 mL of water and 1 mL of absolute ethanol. The product was precipitated by addition of an excess saturated NH_4_PF_6_ aqueous solution. The collected solids were washed twice with water and dried under vacuum to obtain the desired ruthenium (II) complex [(*η*^*6*^*-p*-cymene)Ru(NN)Cl]PF_6_.

[(*η*^*6*^*-p*-cymene)Ru(dppz)Cl]PF_6_ (**Ru-1**) [27] have been reported, NMR data of these complexes were identical to the related references.

[(*η*^*6*^*-p*-cymene)Ru(*o-*Me-dppz)Cl]PF_6_ **(Ru-2)**: ^1^H NMR(400 MHz, DMSO-d_6_) δ 9.99 (dt, *J* = 5.5, 1.5 Hz, 2H), 9.73 (ddd, *J* = 23.6, 8.2, 1.3 Hz, 2H), 8.38 – 8.17 (m, 3H), 7.98 (dt, *J* = 9.9, 6.9 Hz, 2H), 6.38 (t, *J* = 5.7 Hz, 2H), 6.15 (t, *J* = 5.6 Hz, 2H), 2.97 (s, 3H), 2.68 (p, *J* = 7.0 Hz, 1H), 2.21 (s, 3H), 0.98 (dd, *J* = 6.9, 1.5 Hz, 6H).^13^C NMR (101 MHz, DMSO-d_6_) δ 157.68, 157.61, 148.32, 148.23, 142.40, 141.33, 138.97, 138.12, 137.92, 135.74, 135.68, 132.71, 132.16, 129.99, 129.76, 128.06, 127.46, 105.23, 103.28, 86.44, 86.40, 84.65, 84.63, 30.93, 22.19, 18.72, 17.20. HRMS(ESI): calcd for C_29_H_26_ClN_4_Ru (M-PF_6_)^+^ 567.0889, found 567.0883.

[(*η*^*6*^*-p*-cymene)Ru(F-Cl-dppz)Cl]PF_6_ **(Ru-3)**: ^1^H NMR(400 MHz, CD_3_CN) δ 9.72 (dddd, *J* = 14.3, 6.2, 3.1, 1.6 Hz, 4H), 8.63 (dd, *J* = 7.7, 1.4 Hz, 1H), 8.27–8.16 (m, 3H), 6.11–6.05 (m, 2H), 5.89 (d, *J* = 6.3 Hz, 2H), 2.74 (p, *J* = 6.9 Hz, 1H), 2.22 (s, 3H), 1.06 (dd, *J* = 6.9, 1.3 Hz, 6H).^13^C NMR (101 MHz, CD_3_CN) δ 161.09, 158.54, 157.82, 157.72, 149.07, 148.93, 142.75, 142.63, 140.61, 140.43, 140.09, 136.26, 136.10, 131.38, 130.39, 130.27, 129.09, 128.86, 128.37, 114.39, 114.17, 106.62, 103.57, 86.52, 85.15, 31.50, 21.81, 18.53. HRMS (ESI): calcd for C_28_H_22_Cl_2_FN_4_Ru(M-PF_6_)^+^ 605.0249, found 605.0244.

[(*η*^*6*^*-p*-cymene)Ru(Cl_2_-dppz)Cl]PF_6_ **(Ru-4)**: ^1^H NMR(400 MHz, CD_3_CN) δ 9.76 (dd, *J* = 5.4, 1.3 Hz, 2H), 9.70 (dd, *J* = 8.2, 1.3 Hz, 2H), 8.65 (s, 2H), 8.19 (dd, *J* = 8.2, 5.4 Hz, 2H), 6.09 (d, *J* = 6.2 Hz, 2H), 5.90 (d, *J* = 6.2 Hz, 2H), 2.74 (p, *J* = 7.0 Hz, 1H), 2.22 (s, 3H), 1.06 (d, *J* = 6.8 Hz, 6H).^13^C NMR (101 MHz, CD_3_CN) δ 157.88, 149.17, 141.78, 140.81, 137.17, 136.28, 130.63, 130.36, 128.42, 106.74, 103.62, 86.54, 85.19, 31.53, 21.85, 18.55.HRMS(ESI): calcd for C_28_H_22_Cl_3_N_4_Ru (M-PF_6_)^+^ 620.9954, found 620.9955.

[(*η*^*6*^*-p*-cymene)Ru(Medpq)Cl]PF_6_ **(Ru-5)**: ^1^H NMR(400 MHz, CD_3_CN) δ 9.73 (ddd, *J* = 6.7, 5.4, 1.3 Hz, 2H), 9.63 (ddd, *J* = 15.0, 8.3, 1.3 Hz, 2H), 9.08 (s, 1H), 8.22 – 8.11 (m, 3H), 7.36 (dtd, *J* = 21.4, 7.0, 1.7 Hz, 1H), 6.12 – 6.02 (m, 2H), 5.92 – 5.81 (m, 2H), 2.89 (s, 3H), 2.71 (hept, *J* = 6.9 Hz, 1H), 2.20 (s, 3H), 1.03 (d, *J* = 6.9 Hz, 6H).^13^C NMR (101 MHz, CD_3_CN) δ 157.53, 157.08, 156.80, 147.97, 147.72, 147.23, 138.73, 137.17, 135.87, 135.66, 130.32, 130.21, 127.82, 127.78, 106.36, 103.42, 86.46, 85.03, 31.46, 22.33, 21.78, 18.48.HRMS (ESI): calcd for C_25_H_24_ClN_4_Ru (M-PF_6_)^+^ 517.0733, found 517.0740.

### X-ray diffraction

Crystal X-ray measurements diffraction data for complex Ru3 (C28H22Cl2F7N4PRu) were collected on a Bruker D8 Venture METALJET diffractometer equipped with a Ga microfocus source (Ga Kα, λ = 1.34139 Å) at a temperature of 193.00 K. The structure was solved using SHELXT and refined with SHELXL via the Olex2 interface. Crystallographic data have been deposited with the Cambridge Crystallographic Data Centre under CCDC 2499955.

Crystals of Ru3 suitable for X-ray diffraction were obtained from a acetone solution. The structural analysis reveals the expected dimer, with the aromatic planar ring of the indane occupying three positions of the distorted octahedron of coordination. This specific molecular geometry is consistent with the general structure presented for the **R** series. Crystal Data for C_28_H_22_Cl_2_F_7_N_4_PRu (M=750.43 g/mol): monoclinic, space group C2/m a =24.0591(12) Å, b=11.8552(6) Å, *c* = 33.6595(6) Å, β=113.045(2)°, *V* =3585.1(3) Å^3^, Z=4, *T*=193 K, μ(CuKα)=5.865 mm^−1^, *Dcalc*= 1.390 g/cm^3^, 16,377 reflections which were used in all calculations, The final R_1_ was 0.0713 (I > 2σ(I)) and wR_2_ was 0.1997 (all data).

### Cell culture

Human lung cancer cells lines A549 and PC9 were obtained from the American Type Culture Collection (ATCC). Cells were routinely maintained in RPMI-1640 (Boster Biological Technology, China) and DMEM (Hyclone, Utah, USA) medium supplemented with 10% of fetal bovine serum (Hyclone), penicillin (10^7^ U/L) and streptomycin (10 mg/L) in a humidified incubator containing 5% CO_2_ at 37 °C. Cell lines were tested by short tandem repeat analysis, and the last time of authentication for these cells was September 2024. All cell lines were regularly confirmed to be free of mycoplasma contamination.

### Establishment of cisplatin-resistant cell lines

To generate cisplatin-resistant cell lines, PC-9 and A549 cells were exposed to 1μM cisplatin for a duration of 7 days. Following this, the cisplatin-containing medium was aspirated and replaced with fresh, cisplatin-free medium to allow cellular recovery and growth. Once the cells had stabilized, they were passaged. This treatment regimen was repeated, with gradual increases in cisplatin concentration each time after the cells had achieved stable growth. Through iterative treatments and concentration escalations, we successfully selected cisplatin-resistant cell lines capable of sustained growth and passage in an environment enriched with high concentrations of cisplatin.

### Assessment of cell viability

The viability of the selected cisplatin-resistant cell lines was evaluated using the CCK-8 assay. Specifically, cells were plated into 96-well plates at a density of 6000 cells per well. Post-treatment, CCK-8 reagents (sourced from Bimake, product code B34302) were added, and the plates were incubated for an hour at the appropriate conditions. Subsequently, the absorbance was quantified at a wavelength of 450 nm to ascertain cell viability.

To investigate the long-term impact of Ru3 on the proliferation of cisplatin-resistant cell lines, colony formation assays were conducted. After treatment, cells were plated into twelve-well plates at a density of 2000–3000 cells per well. The culture medium containing Ru3 was replaced every three days to maintain consistent exposure. After a period of two weeks, the colonies were fixed with 4% paraformaldehyde (Sigma) for 30 min and stained with Crystal Violet for another 30 min. The colonies were then rinsed thoroughly three times and photographed for documentation.

Further insights into cell proliferation were gained using the EdU assay. Cells grown on coverslips were treated with Ru3 for 24 or 48 h and subsequently stained with EdU (procured from RiBoBio, Guangzhou, China) for two hours. Excess dye was meticulously washed off according to the manufacturer's instructions. Nuclei were stained with DAPI for 10 min and observed under a fluorescence microscope (Leica, Frankfurt, Germany) for the analysis of cellular proliferation.

### Reagents and antibodies

Antibodies were shown in Table S1.

NAC (HY-B0215), Mito-TEMPO (HY-112879), HLM006474 (HY-16667), Z-VAD-FAK (HY-16658B), irinotecan (HY-13001), docetaxel (HY-13065), Necrostatin-1 (HY-15760), NLRP3-IN-78 (HY-144623), 4-PBA (HY-100457), Fer-1 (HY-100579), RSL3 (HY-100218) were purchased from MCE.

### LDH cytotoxicity assays

The cytotoxicity was determined by the lactate dehydrogenase (LDH) detection kits (C0016, Beyotime). The cell culture supernatants were collected to detect the released LDH according to the instructions. Briefly, 60 µL of the supernatant samples were transferred to a 96-well plate and mixed with 30 µL LDH working solution and incubated at room temperature for 30 min. The percentage of the LDH released into the culture medium was measured.

### Live and dead cells staining

To evaluate cell viability post-Ru3 treatment, 6,000 cells per well were plated in a 96-well plate and incubated for 24 h. Subsequently, the cells were exposed to Ru3. After treatment, the cells were washed with PBS to eliminate potential interferences. Utilizing a staining kit from Beyotime Biotechnology (Beyotime C2013L), the cells were stained according to the manufacturer's protocol. During staining, live cells converted the dye into a green fluorescent product, while dead cells, due to their compromised membranes, stained red. After a 15-min incubation at 37 °C, the cells were washed and analyzed under a fluorescence microscope (Leica, Frankfurt, Germany).

#### Immunoblotting

Cells were lysed with RIPA buffer (Beyotime) supplemented with the protease inhibitor cocktail (Sigma) for 30 min on ice. The protein lysate was sonicated on ice and centrifuged at 14,000 rpm at 4°C for 15 min. Protein concentrations were determined using the Quick StartTM Bradford 1× dye reagent (500–0205, Bio Rad) before boiling with the loading buffer. Proteins were separated on the SDS-PAGE gel (Bio-Rad) and transferred to the PVDF membranes (Millipore, United States), where they were blocked with 5% skimmed milk. The membranes were incubated with the corresponding primary antibodies at 4 °C overnight and HRP-conjugated secondary antibodies for 1 h at room temperature. Specific protein bands were visualized with a ChemiDoc image detector (Bio-Rad).

#### Quantitative RT-PCR (qRT-PCR)

Total RNA was extracted using Trizol reagent (Invitrogen) and the reverse transcription was carried out using the Reverse Transcription PrimeScript 1st Stand cDNA Synthesis kit (TaKaRa, Otsu, Japan). q-PCR was performed using the quantitative PCR reagents SYBR PremixEx TaqTM (TaKaRa) following the manufacturer’s instructions. Levels of GAPDH were quantified as internal controls and the fold-changes were analyzed using the 2^−ΔΔCt^ method. The primer sequences were listed in Table S2.

#### Transfection

All siRNAs were designed using BLOCK-iT™ RNAi Designer (Invitrogen) and synthesized by GenePharma (Shanghai, China). The sequences of the siRNAs used are listed in Table S2. Cells were collected and subjected to subsequent analysis after transfection 24h-72h.

The IGFBP3 overexpression plasmid was a gift of Dr. Hiroshi Nakagawa (PA, USA) [29] and its control vector plasmid was preserved in our laboratories. Short hair IGFBP3 shRNA synthesized by Sangon Biotech (Shanghai, China) was inserted into the psi-LVRH1GP via enzyme digestion, and the IGFBP3 shRNA plasmid was constructed through transformation, selection, and plasmid extraction. The target sequence for IGFBP3 shRNA is as follows: 5′-GCACAGATACCCAGAACTT-3′.

The pCDH-shIGFBP3 plasmid was packaged with lentivirus, and the 293T cell line was used to package the lentivirus skeleton and plasmid [29]. The virus solution was harvested and then infected with cells. Two days after infection, puromycin was used for screening for another 7 days.

#### Reactive oxygen species (ROS) measurement

The measurement of Reactive Oxygen Species (ROS) was conducted in accordance with the manufacturer’s instructions. Specifically, the cells were stained with 2',7'-dichlorofluorescein diacetate (DCFH-DA) (GENMED, catalog number GMS10016.2, Shanghai, China) to detect the intracellular ROS level. Following staining, the DCFH-DA signal was analyzed using a FACS Calibur flow cytometer (BD Biosciences), allowing for the quantification of ROS levels within the cell population.

#### Cell cycle and apoptosis analysis

Cell cycle distribution was assessed using flow cytometry following staining with propidium iodide. Briefly, both floating and adherent cells were harvested, washed with ice-cold phosphate-buffered saline (PBS), and subsequently fixed with 75% ethanol. The fixed cells were then incubated with 50 μg/ml of RNaseA and 50 μg/ml of propidium iodide for 30 min at room temperature. The stained cells were analyzed using a FACS can flow cytometer (BD Biosciences) to determine the cell cycle distribution.

For apoptosis analysis, cells were stained with Annexin V-FITC and propidium iodide utilizing the Annexin V-FITC Apoptosis Detection Kit (Sigma, Saint Louis, USA). Following staining, the cells were subjected to FACS analysis to quantify the level of apoptosis.

#### Transmission electron microscopy

Autophagic vesicles and mitochondria were observed using transmission electron microscopy. Briefly, cells were treated with Ru3 for 24 h. Following treatment, the cells were fixed using glutaraldehyde (Sigma). Ultrathin sections were then prepared with the aid of a Sorvall MT5000 microtome. These sections were stained with either lead citrate or 1% uranyl acetate, or a combination of both, and subsequently visualized under a Philips EM420 electron microscope to observe the autophagic vesicles.

#### TUNEL assay procedure

Cells were treated with Ru3 for 24 h, followed by two washes with phosphate-buffered saline (PBS, pH 7.4). The cells were then fixed with 4% paraformaldehyde for 25 min. After fixing, the samples were washed again twice with PBS and permeabilized with 0.2% Triton X-100 for 5 min. Next, the cells were equilibrated with equilibration buffer for 7 min. Nucleotide mix and rTdT were prepared for both test and control samples. DNA strand breaks were labeled with fluorescein 12-dUTP and incubated for 60 min in a humidified chamber at 37 °C. Following incubation, the reactions were stopped using SSC solution. The samples were then washed three times with PBS to remove any unincorporated fluorescein (12-dUTP) and immediately analyzed using a fluorescence microscope (Leica, Frankfurt, Germany) to detect the labeled DNA strand breaks.

#### Animal experiments

All animal experiments in this study were carried out in compliance with approved animal protocols and ethical guidelines set by the Animal Ethics Committee of Chongqing Medical University (Certification Approval number:IACUC-CQMU-2024-0026).

To investigate the in vivo effects of Ru3, 4–6 week old NSG mice were used in this study. A tumor xenograft model was established by subcutaneous injection of PC9-DDP cells (1×10⁷). When tumors reached a volume of 100 mm^3^, the mice were randomly divided into control and treatment groups, with four mice in each group. The treatment group received daily tail vein injections of Ru3 (5 mg/kg), while the control group received DMSO.

Tumor growth was monitored weekly using vernier calipers, and volumes were calculated using the formula: volume = length×width^2^ × 0.5. After 15 days, mice were euthanized, and tumors were collected for further analysis. Hematoxylin–eosin staining and immunohistochemistry were performed on tumor tissues to evaluate histological changes and protein expression, respectively.

#### Immunohistochemistry

The tissue samples were initially fixed in 4% paraformaldehyde (Biosharp, BL539A) and then embedded in paraffin. Sections of 3 µm thickness were obtained and subsequently dewaxed using xylene. To facilitate antigen retrieval, the sections were heated in a citrate solution for 15 min. This was followed by a 10-min incubation in a 3% hydrogen peroxide solution to block endogenous peroxidase activity. The sections were then blocked with normal goat serum for 20 min.

Next, the tissue sections were incubated overnight at 4 °C with primary antibodies. After this incubation, the sections were treated with a horseradish peroxidase-conjugated anti-rabbit IgG antibody (ZLI-9017/9018/9019 DAB Kit). The sections were then stained with diaminobenzidine and counterstained with hematoxylin (Biosharp, BS019). Finally, the sections were mounted using neutral gum.

Observations were made in five visual fields per section under a high-power microscope. Based on the cell staining intensity and the proportion of positive cells, mean values were calculated and used for statistical analysis.

#### RNA sequencing analysis

Cells were treated with Ru3 or Dimethyl Sulfoxide (DMSO) for 48 h, following which total RNA was extracted from the cells using TRIzol reagent (provided by Invitrogen). The sequencing work was carried out by Qinke Biotech Co., Ltd. The data from the RNA sequencing (RNA-seq) analysis are presented in Table S3.

#### Wound healing assay

Cell migration capacity was evaluated via wound healing assay. Briefly, cisplatin-resistant lung cancer cells were seeded into 6-well culture plates and cultured until full confluence. A sterile 200 μL pipette tip was used to scratch a straight wound across the monolayer, followed by gentle washing with serum-free medium twice to remove detached floating cells. The medium was then replaced with serum-free culture medium containing the indicated concentrations of Ru3 to eliminate cell proliferation interference. Images of the wound area were captured under an inverted microscope at 0 h and 24 h after scratching at consistent positions. The wound closure rate was quantified using ImageJ software to assess the migratory ability of tumor cells.

#### Transwell invasion assay

Cells were resuspended in serum-free medium, and 200 μL cell suspension was seeded into the upper chamber. The lower chamber was filled with 600 μL complete medium containing 10% FBS as chemotactic attractant. Different concentrations of Ru3 were added to upper compartments. After incubation for 24 h at 37 °C, non-migrated cells on the upper membrane surface were wiped off with a cotton swab. Cells that migrated to the lower side of the membrane were fixed with 4% paraformaldehyde and stained with crystal violet.

#### Intracellular Fe^2^⁺ measurement by flow cytometry

Cellular ferrous ion (Fe^2^⁺) levels were quantified using the RhoNox-6 fluorescent probe kit (Beyotime Biotechnology, Cat. No. S1070) according to the manufacturer’s instructions. Briefly, treated lung cancer cells were digested with trypsin, harvested and washed twice with pre-cooled PBS. Cells were resuspended in serum-free medium at a density of 1 × 10⁶ cells/mL, followed by incubation with 5 μM RhoNox-6 working solution at 37 °C for 30 min in the dark. After staining, cells were pelleted by centrifugation at 400 ×*g* for 4 min, washed twice with PBS, and resuspended in 300 μL PBS for subsequent detection. The red fluorescence intensity of RhoNox-6 was immediately determined by flow cytometry, with excitation and emission wavelengths set at 540 nm and 575 nm, respectively.

#### Measurement of MDA content

The intracellular malondialdehyde (MDA) level, a marker of lipid peroxidation, was measured using the MDA detection kit (Beyotime Biotechnology) following the manufacturer’s protocols. Briefly, treated cells were collected and lysed with cell lysis buffer on ice, and the total protein concentration of each lysate was quantified by BCA assay for normalization. Subsequently, the supernatant was mixed with MDA working solution and heated in a water bath at 100 °C for 15 min. After cooling to room temperature, the mixture was centrifuged, and the absorbance of the supernatant was measured at 532 nm using a microplate reader. The MDA content was calculated and normalized to total protein content to reflect the relative lipid peroxidation level of cells.

#### Determination of intracellular GSH levels

Intracellular glutathione (GSH) content was quantified using a GSH detection kit (Beyotime Biotechnology, Cat. No. S0053) following the manufacturer’s instructions. Briefly, treated lung cancer cells were harvested and lysed with ice-cold lysis buffer. The lysate was centrifuged at 12,000 ×*g* for 10 min at 4 °C to collect the supernatant. The total protein concentration of each supernatant was determined by BCA assay for subsequent normalization. The supernatant was mixed with the kit reaction working solution, and the mixture was incubated at room temperature away from light. The absorbance value at 412 nm was measured using a microplate reader. The relative GSH content was calculated according to the standard curve and normalized to the total protein mass to eliminate differences in cell number among groups.

#### Mitochondrial and cytosolic fractionation

Subcellular fractionation to separate cytosolic and mitochondrial fractions was performed using the Mitochondria Isolation Kit for Mammalian Cells (Beyotime Biotechnology, Cat. No. C3601) in accordance with the manufacturer’s guidelines. Briefly, Ru3-treated lung cancer cells were digested, harvested and washed twice with pre-cooled PBS. Cell pellets were resuspended in ice-cold mitochondria isolation reagent supplemented with protease inhibitor cocktail, followed by gentle homogenization on ice. The homogenate was centrifuged at 600 ×*g* for 10 min at 4 °C to remove nuclei and unbroken cells. The resulting supernatant was further centrifuged at 11,000 ×*g* for 10 min at 4 °C. The supernatant collected after this step was retained as the cytosolic fraction, while the sediment was mitochondrial pellet. The mitochondrial precipitate was resuspended in mitochondrial lysis buffer to extract mitochondrial protein. Both cytosolic and mitochondrial protein samples were quantified via BCA assay and preserved at − 80 °C for subsequent Western blot analysis.

#### Data analysis and statistics overview

The data were meticulously presented as means ± standard deviation (s.d.) to offer a clear understanding of the variability within the datasets. To ensure the reliability and reproducibility of the results, all experiments were rigorously conducted a minimum of three times. For statistical analyses, the advanced GraphPad Prism 8.0 software was employed. The significance of differences between groups was evaluated using a two-tailed Student's t-test, with the levels of significance clearly denoted as follows: *, *P* < 0.05; **, *P* < 0.01; and ***, *P* < 0.001.

## Supplementary Information

Below is the link to the electronic supplementary material.

Supplementary Material 1

Supplementary Material 2

## Data Availability

No datasets were generated or analysed during the current study.

## Abbreviations

PARP: Poly ADP-ribose polymerase
PCNA: Proliferating cell nuclear antigen
TUNEL: TdT-mediated dUTP nick end labeling
Ki-67: Nuclear proliferation antigen Ki-67
LDH: Lactate dehydrogenase
MAP1LC3B/LC3B: Microtubule associated protein 1 light chain 3 beta
mTOR: Mechanistic target of rapamycin kinase
AKT: Protein Kinase B
P53: Tumor protein P53
P21: Cyclin-dependent kinase inhibitor 1A
P27: Cyclin-dependent kinase inhibitor 1B
P16: Cyclin-dependent kinase inhibitor p16INK4a
NAC: N-acetyl-L-cysteine
ROS: Reactive oxygen species
SQSTM1/p62: Sequestosome 1
IGFBP3: Insulin-like growth factor binding protein 3
B-Myb: V-myb myeloblastosis viral oncogene homolog (avian)-like 2
E2F2: E2F Transcription Factor 2
