# Supplementary material for: Ru3: a novel ruthenium-based compound overcomes cisplatin resistance in non-small cell lung cancer via the E2F2-IGFBP3-GPX4 pathway
Source: Apoptosis. 2026 Oct 3;31(10):243. doi: 10.1007/s10495-026-02447-3 (PMC13633971; doi:10.1007/s10495-026-02447-3)
Supplement: Supplementary file 1 [file 10495_2026_2447_MOESM1_ESM.docx]

Supplementary Figure legends

Figure S1

(A) Western blot analysis was performed to detect ERCC1 expression in A549, A549-DDP, PC-9, and PC9-DDP cells. (B-D) A549-DDP and PC9-DDP cells were treated with the indicated concentrations of Ru1, Ru2, Ru3, Ru4, and Ru5 for 24 h, and cell viability was subsequently determined using the CCK-8 kit. (E) A549-DDP and PC9-DDP cells were treated with 5 μM Irinotecan, Docetaxel, or Ru3 for 24 h, and cell viability was subsequently determined using the CCK-8 kit. (F) NL20 cells were treated with the indicated concentrations of Ru3 for 24 h, and cell viability was determined using the CCK-8 kit. (G-H) EdU assays were conducted on NL20 cells after 24 h of treatment with Ru3 at 0, 1, 5 and 10 μM. Scale bar: 250 μm. (I-J) NL20 cells were treated with Ru3 at 0, 1, 5, and 10 μM for 24 h, followed by staining with Calcein-AM and propidium iodide (PI) to calculate the ratio of live to dead cells. Scale bar: 250 μm. (K) LDH release was measured in NL20 cells after treatment with Ru3 at the indicated concentrations. (L-M) Transwell migration assays were performed on A549-DDP and PC9-DDP cells after treatment with Ru3 at the indicated concentrations for 24 h; migrated cells were stained and counted. Scale bar: 100 μm. (N-O) Wound healing (scratch) assays were performed on A549-DDP and PC9-DDP cells after treatment with Ru3 at the indicated concentrations for 24 h; representative images were captured at 0 and 24 h. Scale bar: 200 μm. (P) qRT-PCR analysis was performed to detect the mRNA expression levels of CDK1, P21, P16, and P27 in A549-DDP and PC9-DDP cells after treatment with 5 μM Ru3 for 24 h. (Q) Western blot analysis was performed to detect the protein expression levels of CDK1, P53, P21, P16, and P27 in A549-DDP and PC9-DDP cells after treatment with 5 μM Ru3 for 24 h. (R) Apoptosis was assessed by flow cytometry using Annexin V-FITC/PI double staining in NL20 cells after treatment with Ru3 at the indicated concentrations for 24 h; Doxorubicin was used as a positive control. Data are presented as means ± s.d. and are representative of three independent experiments. *, P < 0.05; **, P < 0.01; ***, P < 0.001. Statistical significance was compared with the respective control groups.

Figure S2

(A-B)The CCK8 assay was performed on A549-DDP and PC9-DDP cells treated with 5 μM Ru3 in the presence or absence of 10 μM Necrostatin-1, 10 μM Docetaxel, or 1mM 4-PBA for 24 hours. (C) Representative images of immunohistochemical staining for Caspase-3, Caspase-9, P16 and P21 in xenografts treated with DMSO or Ru3(5mg/kg). Scale bar: 100 μm. (D) HE staining analysis of tissue sections from organs (heart, liver, spleen, lung, and kidney) of the NSG mice treated with DMSO or Ru3(5mg/kg). Data are presented as means ± s.d. and are representative of three independent experiments. Statistical significance was compared with the respective control groups.

Figure S3. (A) Gene Ontology (GO) enrichment analysis of differentially expressed genes (DEGs) after Ru3 treatment. (B) Western blot analysis was performed to detect the protein expression levels of p-AKT and p-mTOR in A549-DDP and PC9-DDP cells after treatment with 5 μM Ru3 for 24 h. (C) Western blot analysis was performed to detect the protein expression levels of P62 and LC3 in A549-DDP and PC9-DDP cells after treatment with 5 μM Ru3 for 24 h. (D-E) Autophagic vesicles were detected by transmission electron microscopy (TEM) in A549-DDP cells treated with or without 5 μM Ru3 for 24 h. Scale bar: 2 μm. (F-G) Intracellular Fe²⁺ levels were measured by flow cytometry in A549-DDP and PC9-DDP cells after treatment with Ru3 at the indicated concentrations for 24 h. (H) Malondialdehyde (MDA) levels were measured in A549-DDP and PC9-DDP cells after treatment with Ru3 at the indicated concentrations for 24 h. (I-J) The ratio of oxidized to reduced BODIPY™ C11 probe was assessed in A549-DDP cells after treatment with 5 μM Ru3 for 24 h. Scale bar: 250 μm. (K) Glutathione (GSH) levels were measured in A549-DDP and PC9-DDP cells after treatment with Ru3 at the indicated concentrations for 24 h. (L) Cell viability was determined by CCK-8 assay in A549-DDP and PC9-DDP cells treated with 5 μM Ru3 in the presence or absence of 10 μM Fer-1 for 24 h. (M) LDH release was measured in A549-DDP and PC9-DDP cells treated with 5 μM Ru3 in the presence or absence of 10 μM Fer-1 for 24 h. (N) Western blot analysis was performed to detect IGFBP3 protein expression in A549-DDP and PC9-DDP cells after transfection with IGFBP3 overexpression plasmid for 24 h. (O) qRT-PCR analysis was performed to detect IGFBP3 mRNA expression in A549-DDP and PC9-DDP cells after transfection with IGFBP3 overexpression plasmid for 24 h. Data are presented as means ± s.d. and are representative of three independent experiments. *, P < 0.05; **, P < 0.01; ***, P < 0.001. Statistical significance was compared with the respective control groups. n.s., not significant.

Figure S4

(A-B) qRT-PCR analysis was performed to detect IGFBP3 mRNA in A549-DDP and PC9-DDP cells after they were treated with the shIGFBP3 plasmid or pCDH. (C) Western Blot analysis was performed to detect IGFBP3 in IGFBP3-knockdown PC9-DDP cells treated with or without Ru3 (5μM) for 24 hours. (D-E) qRT-PCR analysis was used to detect the mRNA of IL6 and IL8 in IGFBP3-overexpressed PC9-DDP and A549-DDP cells treated with or without Ru3 (5μM) for 24 hours. (F) qRT-PCR analysis was conducted to measure the mRNA levels of IGFBP3 and GPX4 in PC9-Parent and PC9-DDP cells after treatment with 5μM Ru3 for 24 hours. (G) Western Blot analysis was performed to detect the protein levels of IGFBP3 in PC9-Parent/PC9-DDP and A549-Parent/A549-DDP cells following treatment with 5μM Ru3 for 24 hours. *, P < 0.05; **, P < 0.01; ***, P < 0.001. Statistical significance compared with respective control groups.

Figure S5

(A) Mitochondrial and cytosolic fractions of A549-DDP and PC9-DDP cells were isolated, and Western blot analysis was performed to detect TOMM20 protein levels in the mitochondrial and cytosolic fractions. VDAC was used as a mitochondrial marker, and GAPDH was used as a loading control for total cell lysates. (B-C) Colocalization of MitoTracker Red and DCFH-DA was analyzed in A549-DDP cells treated with or without 5 μM Ru3 for 24 h. Scale bar: 10 μm. (D-E) PC9-DDP and A549-DDP cells were treated with 5 μM Ru3 in the presence or absence of 100 μM Mito-TEMPO for 24 h, followed by staining with Calcein-AM and propidium iodide (PI) to calculate the ratio of live to dead cells. Scale bar: 250 μm. (F) EdU incorporation assays were performed on A549-DDP and PC9-DDP cells treated with 5 μM Ru3 in the presence or absence of 100 μM Mito-TEMPO for 24 h; EdU-positive cells were quantified. Scale bar: 250 μm. (G) Immunoblot analysis was performed to detect IGFBP3 protein levels in PC9-DDP cells treated with 5 μM Ru3 alone or in combination with 5 mM N-acetylcysteine (NAC) for 24 h. (H) Immunoblot analysis was performed to detect IGFBP3 protein levels in PC9-DDP cells treated with 5 μM Ru3 alone or in combination with 100 μM Mito-TEMPO for 24 h. Data are presented as means ± s.d. and are representative of three independent experiments. ***, P < 0.001. Statistical significance was compared with the respective control groups.

Figure S6

(A-B) Immunoblot analysis was performed to detect B-Myb in A549-DDP and PC9-DDP cells after 24 hours of treatment with the indicated concentrations of Ru3. (C) qRT-PCR analysis was conducted to measure B-Myb mRNA levels in PC9-DDP cells following 24 hours of transfection with siB-Myb or siNC. (D) The mRNA levels of IGFBP3 were assessed by qRT-PCR in PC9-DDP and A549-DDP cells that had been transfected with siB-Myb or siNC, following treated with or without 5μM Ru3 for 24 hours. (E) Western-Blot analysis was employed to detect IGFBP3, B-Myb, and p-B-Myb protein levels in PC9-DDP cells after transfection with siB-Myb or siNC, following treated with or without 5μM Ru3 for 24 hours. (F) The cell viability of A549-DDP and PC9-DDP cells, which had been transfected with siB-Myb and treated with 5μM Ru3 for 48 hours, was evaluated using CCK-8 assays. (G-H) EdU assays were conducted on PC9-DDP cells after 48 hours of treatment with 5μM Ru3, combined with transfection of siB-Myb or siNC. Scale bar 250 μm. (I) qRT-PCR analysis was performed to detect E2F1 mRNA in PC9-DDP cells transfection with siE2F1 or siNC. (J) The mRNA levels of IGFBP3 were measured by qRT-PCR in PC9-DDP and A549-DDP cells after transfection with siE2F1 or siNC, following treated with or without 5μM Ru3 for 24 hours. **, P < 0.01; ***, P < 0.001. Statistical significance compared with respective control groups.

Figure S4.

 (A) The binding site of E2F2 in the IGFBP3 promoter. Data sourced from the JASPAR database. (B) Schematic diagram for construction of IGFBP3 luciferase promoter. (C) qRT-PCR was performed to detect IGFBP3 mRNA levels in PC9-DDP cells after 24 h of treatment with 5 μM Ru3 in the presence or absence of 10 μM HLM006474. (D-E) CCK-8 assays were performed on PC9-DDP cells that had been transfected with siE2F2 or siNC and subsequently treated with 5 μM Ru3 for 24 or 48 h. (F) Immunoblot analysis was performed to detect GPX4 protein expression in A549-DDP cells after siRNA-mediated knockdown of GPX4. (G) CCK-8 assays were performed on PC9-DDP cells that had been transfected with siGPX4 or siNC and subsequently treated with 5 μM Ru3 for 24 h. (H-I) Live/dead cell staining was performed on PC9-DDP cells after treatment with 5 μM Ru3 for 24 h following transfection with siGPX4 or siNC. Scale bar: 250 μm. (J-K) EdU proliferation assays were performed on PC9-DDP cells after treatment with 5 μM Ru3 for 24 h following transfection with siGPX4 or siNC. Scale bar: 250 μm. (L) CCK-8 assays were performed on A549-DDP cells treated with 5 μM Ru3 in the presence or absence of 10 μM RSL3 for 24 h. (M-N) Live/dead cell staining was performed on A549-DDP cells after treatment with 5 μM Ru3 in the presence or absence of 10 μM RSL3 for 24 h. Scale bar: 250 μm. (O) LDH release was measured in A549-DDP and PC9-DDP cells after treatment with 5 μM Ru3 in the presence or absence of 10 μM RSL3 for 24 h. Data are means ± s.d. and are representative of 3 independent experiments. *, P < 0.05; **, P < 0.01; ***, P < 0.001. Statistical significance was compared with the respective control groups. n.s., not significant.

**Supplementary Table 1:** Antibodies used in the present study

| Protein name | Manufacturer (cat.number) | Applications  (working dilution) |
| --- | --- | --- |
| β-actin | ZENBIO (R23613) | WB (1:1000) |
| MAP1LC3B | Cell Signaling Technology(4108S) | WB (1:1000) |
| SQSTM1/p62 | MBL International corporation (PM045) | WB (1:1000) |
| Phospho-Akt | Cell Signaling Technology(4060T) | WB (1:1000) |
| GAPDH | Biosciences (AF7021) | WB (1:1000) |
| PARP1 | Cell Signaling Technology(9542S) | WB (1:1000) |
| mTOR | Cell Signaling Technology(2983S) | WB (1:1000) |
| PCNA | Cell Signaling Technology(D3H8P) | IHC (1:100) |
| Ki-67 | Cell Signaling Technology(9129T) | WB (1:1000) |
| Caspase-3 | Cell Signaling Technology(9962s) | WB (1:1000) |
| IGFBP3 | Santa (SC-135947) | WB (1:200) |
| E2F2 | Santa (SC-515402) | WB (1:200) |
| GPX4 | Santa (SC-166570) | WB (1:200) |
| P53 | Cell Signaling Technology(2527T) | WB (1:1000) |
| P21 | Cell Signaling Technology(12D1) | WB (1:1000) |
| P16 | Cell Signaling Technology(80772T) | WB (1:1000) |
| Caspase-9 | Cell Signaling Technology(9052T) | WB (1:1000) |
| P27 | Cell Signaling Technology(3686T) | WB (1:1000) |
| CDK1 | Cell Signaling Technology(18048T) | WB (1:1000) |
| Bcl-2 | Cell Signaling Technology(3498T) | WB (1:1000) |
| ERCC1 | Cell Signaling Technology(D61F5) | WB (1:1000) |
| TOMM20 | Proteintech(80501-1-RR) | WB (1:1000) |
| VDAC | Cell Signaling Technology(4866S) | WB (1:1000) |
| Anti-Rabbit IgG | Proteintech (SA00001-2) | WB (1:5000) |
| Anti-Mouse IgG | Proteintech (SA00001-1) | WB (1:5000) |

**Supplementary Table 2:** Oligonucleotides used in the present study

Primers used for RT-PCR analysis

| Gene name | Primer sequence |
| --- | --- |
| GAPDH | F: 5'-ACCTGACCTGCCGTCTAGAA-3'  R:5'-TCCACCACCCTGTTGCTGTA-3' |
| P21 | F: 5ʹ-GTGGATGGGTTGGTCAGTCCCT-3ʹ  R:5ʹ- AGGGCATCCAAGTCGAACTCCT-3ʹ |
| P16 | F: 5ʹ-GCACTTCGGCAGAGTTGAATG-3ʹ  R:5ʹ-AGCTTCAGCCGAGTTACAGG-3ʹ |
| P53 | F: 5ʹ-AAGTACGGCAACCTCTCCAA-3ʹ  R:5ʹ-CCTCAGCTTCTTGGTCTGGA-3ʹ |
| IGFBP3 | F: 5ʹ-TCTGATCCCAAGTTCCACCC-3ʹ  R:5ʹ- TCCATTTCTCTACGGCAGGG-3ʹ |
| B-Myb | F: 5ʹ-AAGATGTTGCCAGGGAGGA-3ʹ  R:5ʹ-TGGTCAGAAGACTTCCCTGG-3ʹ |
| E2F1 | F: 5ʹ-TGGACTTCACCTTGGGAGAC-3ʹ  R:5ʹ-ATCAGTCACATCAGGGCTTGT-3ʹ |
| E2F2 | F: 5ʹ-AGCAGAGTTTTGGGATGGGA-3ʹ  R:5ʹ-CAGACAGGACACGAAATGCC-3ʹ |
| RUNX1 | F: 5'-TCTTCACAAACCCACCGCAA-3'  R: 5'-CTGCCGATGTCTTCGAGGTTC -3' |
| YAP1 | F: 5'-TCCAAGTGCCGAAAAAGGAAG-3'  R: 5'-CTGCCGATGTCTTCGAGGTTC -3' |
| STAT5 | F: 5'-TCCAAGTGCCGAAAAAGGAAG-3'  R: 5'-CTGCCGATGTCTTCGAGGTTC -3' |
| E2F3 | F: 5'-GGAAGTGAAAACATTGGCCG-3'  R: 5'-ATGTTCTTGTACGCCCTCCA-3' |
| MRPL35 | F: 5'-TCCAAGTGCCGAAAAAGGAAG-3'  R: 5'-CTGCCGATGTCTTCGAGGTTC -3' |
| MRPL40 | F: 5'-TCCAAGTGCCGAAAAAGGAAG-3'  R: 5'-CTGCCGATGTCTTCGAGGTTC -3' |
| MRPS14 | F: 5'-GAATGTGGCGCGATGTGAAG -3'  R: 5'-GGGACGTCATAACACACCGA -3' |
| MRPS28 | F: 5'-ACAGATGGGACCTGCAAAGG-3'  R: 5'-TCTAATAGCCGCAACCGGAC-3' |
| GPX4 | F: 5'-ATACGTGGATTGAGGACCACT-3'  R: 5'-TCCAATGTCAAGTAGCGGTTG -3' |

List of Small Interference RNA Sequence

| Gene name | sequences (5'-3') |
| --- | --- |
| E2F1 | UGGAAUUACUCACUGAUAA |
| E2F2 | GCCACCUUGCUUGUACCAA |
| B-Myb | GCAACAAGCCCGUGUCCUA |
| GPX4 | CAGGGAGUAACGAAGAGAUUU |
