# Supplementary material for: Ru3: a novel ruthenium-based compound overcomes cisplatin resistance in non-small cell lung cancer via the E2F2-IGFBP3-GPX4 pathway
Source: Apoptosis. 2026 Oct 3;31(10):243. doi: 10.1007/s10495-026-02447-3 (PMC13633971; doi:10.1007/s10495-026-02447-3)
Supplement: Supplementary file 2 [file 10495_2026_2447_MOESM2_ESM.docx]

| Gene ID | Gene Symbol | Type | log2 (w16 / con) | Qvalue (w16 / con) |  |
| --- | --- | --- | --- | --- | --- |
| 100133941 | 'CD24' | mRNA | -0.589310997 | 1.3239732692887101E-10 |  |
| 100302652 | 'GPR75-ASB3' | mRNA | 2.08845695318725 | 0.0026156289468072 |  |
| 1004 | 'CDH6' | mRNA | -0.825333542 | 4.98021385370763E-5 |  |
| 100462977 | 'MTRNR2L1' | mRNA | -1.159958992 | 0.0094499881806108 |  |
| 100462981 | 'MTRNR2L2' | mRNA | -1.033704759 | 0.0063819482779443 |  |
| 100462983 | 'MTRNR2L3' | mRNA | -1.281077655 | 4.405288790300609E-11 |  |
| 100463285 | 'MTRNR2L4' | mRNA | -1.188763811 | 0.0104108997902297 |  |
| 100463482 | 'MTRNR2L6' | mRNA | -1.215542243 | 0.022736591209885 |  |
| 100463486 | 'MTRNR2L8' | mRNA | -1.128344938 | 0.0080266647733971 |  |
| 100463487 | 'MTRNR2L9' | mRNA | -1.148336098 | 0.0010051723280912 |  |
| 100463488 | 'MTRNR2L10' | mRNA | -1.146816179 | 0.0191882166994526 |  |
| 100505876 | 'CEBPZOS' | mRNA | -0.593273511 | 1.749562715593E-4 |  |
| 10051 | 'SMC4' | mRNA | -0.533058464 | 0.0130301104357879 |  |
| 100526832 | 'PHOSPHO2-KLHL23' | mRNA | -1.355637458 | 0.0125746876486468 |  |
| 10053 | 'AP1M2' | mRNA | -0.53850029 | 0.0021519638908948 |  |
| 10055 | 'SAE1' | mRNA | 0.294374915315543 | 0.0112206603216073 |  |
| 10060 | 'ABCC9' | mRNA | -2.046508703 | 0.0143795712401377 |  |
| 10067 | 'SCAMP3' | mRNA | 0.360191535279657 | 0.0140304145842682 |  |
| 10075 | 'HUWE1' | mRNA | 0.341226596114405 | 8.519950423093E-4 |  |
| 10082 | 'GPC6' | mRNA | -0.495876179 | 0.030041372332842 |  |
| 100996758 | 'NPY4R2' | mRNA | -0.796262461 | 0.0141183198999333 |  |
| 101060351 | 'TBC1D3K' | mRNA | -6.062520907 | 0.0227766595395001 |  |
| 10111 | 'RAD50' | mRNA | -0.705385849 | 7.12597483940031E-7 |  |
| 10123 | 'ARL4C' | mRNA | -0.32989292 | 0.0133084851111497 |  |
| 10128 | 'LRPPRC' | mRNA | -0.389795884 | 2.237731830927E-4 |  |
| 10142 | 'AKAP9' | mRNA | -1.087234178 | 1.17997169358206E-5 |  |
| 1015 | 'CDH17' | mRNA | -0.675189615 | 2.51975884646608E-9 |  |
| 10153 | 'CEBPZ' | mRNA | -0.432872196 | 0.0305480118121378 |  |
| 10155 | 'TRIM28' | mRNA | 0.360990754513859 | 0.0125746876486468 |  |
| 10198 | 'MPHOSPH9' | mRNA | -0.579186817 | 0.0403301455891643 |  |
| 10207 | 'PATJ' | mRNA | -0.436917993 | 0.0227766595395001 |  |
| 10257 | 'ABCC4' | mRNA | -0.372573094 | 0.0023573357736258 |  |
| 102723502 | 'LOC102723502' | mRNA | -2.818309403 | 0.0187336683999952 |  |
| 102723996 | 'LOC102723996' | mRNA | 1.61111354043853 | 0.0437669068160489 |  |
| 102724788 | 'LOC102724788' | mRNA | -0.649315676 | 0.0139466840890753 |  |
| 10276 | 'NET1' | mRNA | -0.380330997 | 0.0079513880554253 |  |
| 10291 | 'SF3A1' | mRNA | 0.321087678666656 | 0.0143337118352532 |  |
| 10317 | 'B3GALT5' | mRNA | -1.090722699 | 2.36529251296699E-7 |  |
| 10333 | 'TLR6' | mRNA | -0.749050584 | 0.0061226010060261 |  |
| 10371 | 'SEMA3A' | mRNA | -0.667865958 | 0.0098112625567237 |  |
| 10381 | 'TUBB3' | mRNA | 0.565444750450525 | 2.86403438351638E-7 |  |
| 10437 | 'IFI30' | mRNA | 0.384887614073422 | 0.0275781041561937 |  |
| 10444 | 'ZER1' | mRNA | 0.558167058747714 | 5.760028895075E-4 |  |
| 10459 | 'MAD2L2' | mRNA | 0.534307421084084 | 0.0142367840318916 |  |
| 1048 | 'CEACAM5' | mRNA | -0.451388133 | 1.27314754895533E-6 |  |
| 10493 | 'VAT1' | mRNA | 0.400591729856307 | 8.432995081775E-4 |  |
| 10505 | 'SEMA4F' | mRNA | 0.606655761258344 | 4.47528206487128E-5 |  |
| 10529 | 'NEBL' | mRNA | -0.769381482 | 8.719073149961E-4 |  |
| 105375355 | 'UPK3B' | mRNA | -0.746087916 | 5.93562435985689E-6 |  |
| 10550 | 'ARL6IP5' | mRNA | -0.356414146 | 0.0111230356705167 |  |
| 10551 | 'AGR2' | mRNA | -1.075332697 | 7.66763980987812E-5 |  |
| 10592 | 'SMC2' | mRNA | -0.763145647 | 0.0280539708806987 |  |
| 10594 | 'PRPF8' | mRNA | 0.292089200681886 | 0.0077929532286082 |  |
| 10600 | 'USP16' | mRNA | -0.623054947 | 0.030306365503813 |  |
| 10602 | 'CDC42EP3' | mRNA | -0.337561905 | 0.0148195932603836 |  |
| 10606 | 'PAICS' | mRNA | -0.48861599 | 3.7326584462816E-5 |  |
| 10608 | 'MXD4' | mRNA | 0.353314770072542 | 0.0213055398646055 |  |
| 10611 | 'PDLIM5' | mRNA | -0.636616783 | 7.01913321723606E-13 |  |
| 10614 | 'HEXIM1' | mRNA | 0.31938043091436 | 0.0061226010060261 |  |
| 1062 | 'CENPE' | mRNA | -1.78120691 | 6.039584506504E-4 |  |
| 10625 | 'IVNS1ABP' | mRNA | -0.318784801 | 0.0437669068160489 |  |
| 1063 | 'CENPF' | mRNA | -0.668995535 | 0.0055936900506835 |  |
| 10672 | 'GNA13' | mRNA | -0.40056181 | 0.0226335283363899 |  |
| 10723 | 'SLC12A7' | mRNA | 0.312168060333609 | 0.0254900346655763 |  |
| 10724 | 'OGA' | mRNA | 0.313070267605117 | 0.0227308433433354 |  |
| 10725 | 'NFAT5' | mRNA | -0.668877827 | 7.12379061169552E-6 |  |
| 10735 | 'STAG2' | mRNA | -0.562082738 | 3.977002996646E-4 |  |
| 10752 | 'CHL1' | mRNA | -1.16324613 | 0.0381694381285628 |  |
| 10762 | 'NUP50' | mRNA | -0.38739666 | 0.04651166595209 |  |
| 10776 | 'ARPP19' | mRNA | -0.355890154 | 0.0010464639247482 |  |
| 10788 | 'IQGAP2' | mRNA | -0.872119022 | 0.030041372332842 |  |
| 10818 | 'FRS2' | mRNA | -0.648001115 | 0.0225909783876883 |  |
| 10847 | 'SRCAP' | mRNA | 0.430575954464338 | 4.712534138604E-4 |  |
| 10890 | 'RAB10' | mRNA | -0.324264509 | 0.0025748298828415 |  |
| 10891 | 'PPARGC1A' | mRNA | -1.013583287 | 0.0024320056916647 |  |
| 10892 | 'MALT1' | mRNA | -0.447502568 | 0.0413050577978857 |  |
| 10901 | 'DHRS4' | mRNA | 0.358448651752428 | 0.0283795441960642 |  |
| 10914 | 'PAPOLA' | mRNA | -0.380215277 | 0.0047971238302106 |  |
| 10938 | 'EHD1' | mRNA | 0.364063585656325 | 0.0104108997902297 |  |
| 10956 | 'OS9' | mRNA | 0.259669372126741 | 0.0433910217029745 |  |
| 10962 | 'MLLT11' | mRNA | -0.486813104 | 4.264755402151E-4 |  |
| 10973 | 'ASCC3' | mRNA | -0.458564826 | 0.002225298787856 |  |
| 10985 | 'GCN1' | mRNA | 0.348516984812726 | 0.0086762740233805 |  |
| 11007 | 'CCDC85B' | mRNA | 0.429375559290326 | 0.0132898953653413 |  |
| 11010 | 'GLIPR1' | mRNA | -0.917160956 | 2.71269899029182E-6 |  |
| 11033 | 'ADAP1' | mRNA | 0.669490574007798 | 0.026261655872226 |  |
| 11041 | 'B4GAT1' | mRNA | 0.42186399843798 | 0.0125758062545051 |  |
| 1105 | 'CHD1' | mRNA | -0.564539352 | 0.0148519113536893 |  |
| 1107 | 'CHD3' | mRNA | -0.277366631 | 0.0490428184055384 |  |
| 11073 | 'TOPBP1' | mRNA | -0.63917901 | 0.0028829508651168 |  |
| 11074 | 'TRIM31' | mRNA | -1.224331756 | 5.64975625266012E-9 |  |
| 11101 | 'ATE1' | mRNA | -0.479056613 | 0.0133084851111497 |  |
| 11165 | 'NUDT3' | mRNA | -0.372483398 | 0.0140945810867352 |  |
| 11177 | 'BAZ1A' | mRNA | -0.665045699 | 3.271383580556E-4 |  |
| 11180 | 'WDR6' | mRNA | 0.397453627307996 | 0.0033006356807896 |  |
| 11199 | 'ANXA10' | mRNA | -1.501006457 | 0.0175636460743385 |  |
| 11215 | 'AKAP11' | mRNA | -0.394715809 | 0.0482526677045067 |  |
| 11230 | 'PRAF2' | mRNA | 0.397334016847566 | 0.0372206014505225 |  |
| 11231 | 'SEC63' | mRNA | -0.4449776 | 0.0030569670621474 |  |
| 11244 | 'ZHX1' | mRNA | -0.563047766 | 0.049101908190043 |  |
| 11261 | 'CHP1' | mRNA | -0.360269936 | 0.0043241263451214 |  |
| 112694756 | 'LOC112694756' | mRNA | -0.550630493 | 5.879159952189E-4 |  |
| 11285 | 'B4GALT7' | mRNA | 0.369134381539022 | 0.0320273322414166 |  |
| 112936 | 'VPS26B' | mRNA | 0.365232622098421 | 0.0121596729896778 |  |
| 11342 | 'RNF13' | mRNA | -0.388913379 | 0.028334250907653 |  |
| 11343 | 'MGLL' | mRNA | -0.325212606 | 0.0036978058210198 |  |
| 114787 | 'GPRIN1' | mRNA | 0.41971916589888 | 0.0115329990314469 |  |
| 115 | 'ADCY9' | mRNA | 0.411530082880682 | 0.0049497703893118 |  |
| 116441 | 'TM4SF18' | mRNA | -1.102960889 | 4.32217717278308E-12 |  |
| 1186 | 'CLCN7' | mRNA | 0.385971319331022 | 0.004060226224017 |  |
| 118987 | 'PDZD8' | mRNA | -0.531113455 | 6.92280698345528E-7 |  |
| 120892 | 'LRRK2' | mRNA | -0.913797581 | 1.4115608189971E-5 |  |
| 1209 | 'CLPTM1' | mRNA | 0.292996132599256 | 0.0281792285450662 |  |
| 123 | 'PLIN2' | mRNA | -0.485779101 | 0.0045928405973729 |  |
| 123036 | 'TC2N' | mRNA | -0.444343168 | 0.0155719451754604 |  |
| 124152 | 'IQCK' | mRNA | -1.169363301 | 0.0062110160338243 |  |
| 124540 | 'MSI2' | mRNA | -0.356040799 | 0.0115752000381369 |  |
| 124997 | 'WDR81' | mRNA | 0.472163749498576 | 0.0021519638908948 |  |
| 125058 | 'TBC1D16' | mRNA | 0.385771315285354 | 0.0019715743261679 |  |
| 125476 | 'INO80C' | mRNA | -0.452634219 | 0.047371967376383 |  |
| 126 | 'ADH1C' | mRNA | -1.392532113 | 0.036669073293786 |  |
| 126868 | 'MAB21L3' | mRNA | -1.21226345 | 0.0022836321305463 |  |
| 126917 | 'IFFO2' | mRNA | 0.361738552237173 | 0.0247533147853167 |  |
| 1272 | 'CNTN1' | mRNA | -0.495772142 | 2.65972931750581E-8 |  |
| 127262 | 'TPRG1L' | mRNA | 0.331734022738562 | 0.0280728546494293 |  |
| 1282 | 'COL4A1' | mRNA | -0.359275044 | 0.042520087734146 |  |
| 128434 | 'VSTM2L' | mRNA | -0.390737486 | 0.0226357094259698 |  |
| 1288 | 'COL4A6' | mRNA | -0.732555013 | 1.660206286258E-4 |  |
| 1290 | 'COL5A2' | mRNA | -0.31638123 | 0.0131141693836887 |  |
| 1294 | 'COL7A1' | mRNA | -0.650640709 | 4.99687792594401E-5 |  |
| 13 | 'AADAC' | mRNA | -0.900830739 | 0.0368620864244849 |  |
| 1308 | 'COL17A1' | mRNA | -0.745671792 | 5.64975625266012E-9 |  |
| 131601 | 'TPRA1' | mRNA | 0.629762558960493 | 5.155756998716E-6 |  |
| 132720 | 'FAM241A' | mRNA | -0.69860345 | 0.0011462144041949 |  |
| 134429 | 'STARD4' | mRNA | -0.508326715 | 0.0250214530934595 |  |
| 135228 | 'CD109' | mRNA | -0.272250976 | 0.0263842501660245 |  |
| 1356 | 'CP' | mRNA | -1.138480589 | 6.17086037831968E-17 |  |
| 1368 | 'CPM' | mRNA | -0.747300999 | 0.0044964270901663 |  |
| 1373 | 'CPS1' | mRNA | -0.362122832 | 3.706194859513E-4 |  |
| 1387 | 'CREBBP' | mRNA | 0.32446894534454 | 0.0251353281251274 |  |
| 140710 | 'SOGA1' | mRNA | 0.395762539084353 | 0.001179529195586 |  |
| 140735 | 'DYNLL2' | mRNA | 0.305593018051506 | 0.015363764908844 |  |
| 140809 | 'SRXN1' | mRNA | 0.336402244515321 | 0.001965420716011 |  |
| 140901 | 'STK35' | mRNA | 0.333354673515771 | 0.0426965954983262 |  |
| 1429 | 'CRYZ' | mRNA | -0.353180374 | 0.0281665003517183 |  |
| 1434 | 'CSE1L' | mRNA | -0.489711239 | 3.22385855441801E-5 |  |
| 1453 | 'CSNK1D' | mRNA | 0.291965389101205 | 0.028623856175927 |  |
| 146059 | 'CDAN1' | mRNA | 0.634637445056744 | 0.0025116910989847 |  |
| 1462 | 'VCAN' | mRNA | -0.514882335 | 1.08752664758418E-8 |  |
| 146713 | 'RBFOX3' | mRNA | -0.541037822 | 0.0093543661046839 |  |
| 147 | 'ADRA1B' | mRNA | -0.59831526 | 0.031399643531289 |  |
| 147657 | 'ZNF480' | mRNA | -0.497783816 | 0.031399643531289 |  |
| 1488 | 'CTBP2' | mRNA | -0.295909171 | 0.0487385832649764 |  |
| 1490 | 'CCN2' | mRNA | -0.632124609 | 0.0196794835038587 |  |
| 149603 | 'RNF187' | mRNA | 0.361939525136184 | 0.0047218275828465 |  |
| 151246 | 'SGO2' | mRNA | -1.62630537 | 0.009070219453083 |  |
| 151887 | 'CCDC80' | mRNA | -0.894042612 | 4.3409999115844E-12 |  |
| 153241 | 'CEP120' | mRNA | -0.564977633 | 0.0345592637472811 |  |
| 153830 | 'RNF145' | mRNA | -0.586613016 | 8.96486913489577E-8 |  |
| 154214 | 'RNF217' | mRNA | -0.538916263 | 0.0198837157155149 |  |
| 1543 | 'CYP1A1' | mRNA | 3.03094033200864 | 4.17421589721915E-23 |  |
| 1545 | 'CYP1B1' | mRNA | 1.16568763351716 | 1.2722637982414499E-42 |  |
| 155061 | 'ZNF746' | mRNA | 0.374044796304867 | 0.0268593415754938 |  |
| 155066 | 'ATP6V0E2' | mRNA | 0.385889963008636 | 0.0078552718883246 |  |
| 155465 | 'AGR3' | mRNA | -2.233843455 | 0.0444574435442069 |  |
| 157285 | 'PRAG1' | mRNA | 0.392623148736141 | 8.621646270839E-4 |  |
| 1577 | 'CYP3A5' | mRNA | -0.75846523 | 0.0280539708806987 |  |
| 158358 | 'KIAA2026' | mRNA | -1.011789628 | 3.665775774213E-4 |  |
| 1595 | 'CYP51A1' | mRNA | -0.379687495 | 0.0015245065095642 |  |
| 1604 | 'CD55' | mRNA | -0.365882862 | 0.0250551418085243 |  |
| 160418 | 'TMTC3' | mRNA | -0.531512112 | 0.0065983978956795 |  |
| 160518 | 'DENND5B' | mRNA | -0.455793959 | 0.008835823636077 |  |
| 1609 | 'DGKQ' | mRNA | 0.522416001954163 | 0.0050437457669036 |  |
| 161424 | 'NOP9' | mRNA | 0.330392576248128 | 0.0487385832649764 |  |
| 162427 | 'RETREG3' | mRNA | 0.32868321543516 | 0.030041372332842 |  |
| 1639 | 'DCTN1' | mRNA | 0.375140623614326 | 0.0068038738117322 |  |
| 1642 | 'DDB1' | mRNA | 0.310233246104213 | 0.0307077192350065 |  |
| 1645 | 'AKR1C1' | mRNA | -0.382032122 | 5.76965474637726E-5 |  |
| 1646 | 'AKR1C2' | mRNA | -0.30968739 | 0.0015245065095642 |  |
| 166336 | 'PRICKLE2' | mRNA | -0.382687779 | 0.026214551305587 |  |
| 167153 | 'TENT2' | mRNA | -0.412790812 | 0.0452085544769146 |  |
| 168374 | 'ZNF92' | mRNA | -1.391618943 | 0.0089311962044736 |  |
| 171024 | 'SYNPO2' | mRNA | -0.49777844 | 0.0067520336422507 |  |
| 1728 | 'NQO1' | mRNA | 0.466750417002336 | 1.77927685621174E-7 |  |
| 1730 | 'DIAPH2' | mRNA | -0.606343183 | 0.0275781041561937 |  |
| 1778 | 'DYNC1H1' | mRNA | 0.490917711498455 | 1.81904181120769E-7 |  |
| 1856 | 'DVL2' | mRNA | 0.34461547888203 | 0.0395748023199041 |  |
| 1891 | 'ECH1' | mRNA | -0.362963362 | 0.001341735092995 |  |
| 1894 | 'ECT2' | mRNA | -0.62201586 | 1.50510391729757E-5 |  |
| 1952 | 'CELSR2' | mRNA | 0.313546674837391 | 0.0452322293908754 |  |
| 1955 | 'MEGF9' | mRNA | -0.526985251 | 8.78768672431882E-5 |  |
| 196 | 'AHR' | mRNA | -0.387493445 | 0.0086726127478864 |  |
| 196441 | 'ZFC3H1' | mRNA | -0.575286065 | 0.0157515362950067 |  |
| 196463 | 'PLBD2' | mRNA | 0.357198286347337 | 0.0034895819354088 |  |
| 197259 | 'MLKL' | mRNA | -0.478565226 | 0.0213055398646055 |  |
| 197407 | 'ZNF48' | mRNA | 0.399944672511358 | 0.0393899458614718 |  |
| 2004 | 'ELK3' | mRNA | -0.341811543 | 0.0444116190000999 |  |
| 2012 | 'EMP1' | mRNA | -0.617112776 | 0.0073653725874123 |  |
| 2013 | 'EMP2' | mRNA | -0.594431845 | 0.0091009367920693 |  |
| 2027 | 'ENO3' | mRNA | -0.751662258 | 0.0275781041561937 |  |
| 2033 | 'EP300' | mRNA | 0.37459980297243 | 0.0063133353686767 |  |
| 2040 | 'STOM' | mRNA | -0.441636106 | 4.72905563278158E-5 |  |
| 205717 | 'USF3' | mRNA | -0.728492661 | 0.009070219453083 |  |
| 2059 | 'EPS8' | mRNA | -0.39164221 | 0.0133563087757205 |  |
| 2064 | 'ERBB2' | mRNA | 0.472765724829645 | 4.020263462791E-4 |  |
| 21 | 'ABCA3' | mRNA | 0.431382157268323 | 3.701929145706E-4 |  |
| 2121 | 'EVC' | mRNA | 0.435388500388415 | 0.0114996745719416 |  |
| 2125 | 'EVPL' | mRNA | 0.435383581092978 | 0.0114268203945122 |  |
| 2132 | 'EXT2' | mRNA | 0.314159873654075 | 0.0081382838393492 |  |
| 2137 | 'EXTL3' | mRNA | 0.304494821560698 | 0.041418041019736 |  |
| 214 | 'ALCAM' | mRNA | -0.52729676 | 4.95987177277552E-7 |  |
| 2153 | 'F5' | mRNA | -0.454171614 | 0.0373663985886342 |  |
| 216 | 'ALDH1A1' | mRNA | -0.240756449 | 0.0340710379705528 |  |
| 218 | 'ALDH3A1' | mRNA | 0.463558348999873 | 1.010315567015E-4 |  |
| 219 | 'ALDH1B1' | mRNA | -0.279999079 | 0.0437669068160489 |  |
| 219743 | 'TYSND1' | mRNA | 0.348872165866803 | 0.0406307734751782 |  |
| 220 | 'ALDH1A3' | mRNA | 0.947622962215474 | 2.31323657688064E-21 |  |
| 220002 | 'CYB561A3' | mRNA | 0.38321998950205 | 0.0196243839006006 |  |
| 2201 | 'FBN2' | mRNA | -0.427062161 | 0.0011627575288284 |  |
| 221 | 'ALDH3B1' | mRNA | 0.289332339197889 | 0.0372206014505225 |  |
| 221002 | 'RASGEF1A' | mRNA | -0.658242618 | 0.0157515362950067 |  |
| 221037 | 'JMJD1C' | mRNA | -0.808251896 | 4.93660456483E-4 |  |
| 221981 | 'THSD7A' | mRNA | -0.424101056 | 0.0061240855402526 |  |
| 222229 | 'LRWD1' | mRNA | 0.435149784687887 | 0.0228513195141441 |  |
| 222643 | 'UNC5CL' | mRNA | -0.930603293 | 0.0420924620416666 |  |
| 2241 | 'FER' | mRNA | -0.776250365 | 0.0101612075426193 |  |
| 2267 | 'FGL1' | mRNA | -0.541486716 | 0.0065497181714235 |  |
| 2274 | 'FHL2' | mRNA | -0.564204128 | 5.238712021195E-4 |  |
| 22809 | 'ATF5' | mRNA | 0.393644555727431 | 0.0080475744353161 |  |
| 22837 | 'COBLL1' | mRNA | -0.967369775 | 0.0038032508626476 |  |
| 22846 | 'VASH1' | mRNA | 0.733432620563337 | 1.241424618081E-4 |  |
| 22894 | 'DIS3' | mRNA | -0.526682472 | 0.0074839717770876 |  |
| 22936 | 'ELL2' | mRNA | -0.469614041 | 0.0089009891150603 |  |
| 22943 | 'DKK1' | mRNA | -0.882000957 | 1.60096355593916E-13 |  |
| 22984 | 'PDCD11' | mRNA | 0.312249294840502 | 0.0223907690693749 |  |
| 22994 | 'CEP131' | mRNA | 0.56199828911549 | 0.0294232131395639 |  |
| 23 | 'ABCF1' | mRNA | 0.287506743119233 | 0.0471811879486664 |  |
| 23023 | 'TMCC1' | mRNA | -0.362637812 | 0.0482526677045067 |  |
| 23036 | 'ZNF292' | mRNA | -1.073637434 | 0.0114996745719416 |  |
| 23038 | 'WDTC1' | mRNA | 0.440680249469536 | 0.038395147264727 |  |
| 23053 | 'ZSWIM8' | mRNA | 0.639928043538122 | 2.79388081549454E-6 |  |
| 23057 | 'NMNAT2' | mRNA | -0.631141006 | 0.0067613167761427 |  |
| 23061 | 'TBC1D9B' | mRNA | 0.251822246471969 | 0.0323936094589812 |  |
| 23064 | 'SETX' | mRNA | -0.668637101 | 3.82198700876771E-5 |  |
| 23067 | 'SETD1B' | mRNA | 0.417399757431681 | 0.0162140646551072 |  |
| 23070 | 'CMTR1' | mRNA | 0.39190253363408 | 0.0133084851111497 |  |
| 23072 | 'HECW1' | mRNA | 0.600141462239184 | 0.006006173886165 |  |
| 23074 | 'UHRF1BP1L' | mRNA | -0.704300465 | 0.0063819482779443 |  |
| 23089 | 'PEG10' | mRNA | 0.339025092643589 | 0.0153107696758292 |  |
| 23092 | 'ARHGAP26' | mRNA | -0.299853508 | 0.0382485007331334 |  |
| 23094 | 'SIPA1L3' | mRNA | 0.305371368469652 | 0.0113113224325904 |  |
| 23096 | 'IQSEC2' | mRNA | 0.401817467393962 | 0.0079542656335805 |  |
| 23098 | 'SARM1' | mRNA | 0.537939457399936 | 0.0372120516267916 |  |
| 23118 | 'TAB2' | mRNA | -0.455269663 | 0.0113270292571697 |  |
| 23130 | 'ATG2A' | mRNA | 0.434545470102698 | 0.0227308433433354 |  |
| 23140 | 'ZZEF1' | mRNA | 0.302890415117585 | 0.0359017260427533 |  |
| 23144 | 'ZC3H3' | mRNA | 0.371044206062737 | 0.0294232131395639 |  |
| 23177 | 'CEP68' | mRNA | 0.447688231428644 | 0.0469750448802372 |  |
| 23179 | 'RGL1' | mRNA | -0.504071497 | 0.0438278985881577 |  |
| 23198 | 'PSME4' | mRNA | -0.365783265 | 0.0060133533686861 |  |
| 23207 | 'PLEKHM2' | mRNA | 0.441597826571635 | 0.0038474771038137 |  |
| 23224 | 'SYNE2' | mRNA | -0.857151198 | 3.63017517088456E-7 |  |
| 23230 | 'VPS13A' | mRNA | -0.887854713 | 1.087965944978E-4 |  |
| 23244 | 'PDS5A' | mRNA | -0.61948969 | 3.2013122330008E-6 |  |
| 23262 | 'PPIP5K2' | mRNA | -0.439471638 | 0.0281127661322183 |  |
| 23265 | 'EXOC7' | mRNA | 0.392625176723469 | 7.408195873857E-4 |  |
| 23272 | 'TASOR' | mRNA | -0.524296573 | 0.004716143823848 |  |
| 23293 | 'SMG6' | mRNA | 0.387883235365102 | 0.0079664193376672 |  |
| 23301 | 'EHBP1' | mRNA | -0.468997473 | 1.436344167725E-4 |  |
| 23309 | 'SIN3B' | mRNA | 0.413139254702561 | 0.0461020594018806 |  |
| 23317 | 'DNAJC13' | mRNA | -0.517604947 | 0.0033006356807896 |  |
| 23326 | 'USP22' | mRNA | 0.255111776001826 | 0.0227766595395001 |  |
| 23344 | 'ESYT1' | mRNA | 0.36360297525335 | 0.00515729293216 |  |
| 2335 | 'FN1' | mRNA | -0.32518641 | 0.0016053928356211 |  |
| 23350 | 'U2SURP' | mRNA | -0.520333442 | 3.672870842594E-4 |  |
| 23351 | 'KHNYN' | mRNA | 0.336839786832148 | 0.009093150518822 |  |
| 23352 | 'UBR4' | mRNA | 0.48791551186472 | 1.27455117793074E-6 |  |
| 23357 | 'ANGEL1' | mRNA | 0.524466453423835 | 9.293781588893E-4 |  |
| 23400 | 'ATP13A2' | mRNA | 0.408039625483999 | 0.0059833280044072 |  |
| 23405 | 'DICER1' | mRNA | -0.473635201 | 0.0069961468439293 |  |
| 23464 | 'GCAT' | mRNA | 0.805149982560923 | 0.0266192773616635 |  |
| 23467 | 'NPTXR' | mRNA | 0.434109460707953 | 0.0275781041561937 |  |
| 23468 | 'CBX5' | mRNA | -0.383491146 | 0.0014664182909942 |  |
| 23469 | 'PHF3' | mRNA | -0.565580813 | 0.0057715172162535 |  |
| 23471 | 'TRAM1' | mRNA | -0.341926628 | 5.597202859245E-4 |  |
| 23503 | 'ZFYVE26' | mRNA | 0.479608560032741 | 0.0109827946654693 |  |
| 23512 | 'SUZ12' | mRNA | -0.56097465 | 0.0019981636512049 |  |
| 23523 | 'CABIN1' | mRNA | 0.376585385287747 | 0.0486609977623663 |  |
| 23524 | 'SRRM2' | mRNA | 0.372226862031186 | 0.002308404843124 |  |
| 23589 | 'CARHSP1' | mRNA | 0.30139467040822 | 0.0444116190000999 |  |
| 23595 | 'ORC3' | mRNA | -0.855486662 | 0.0133084851111497 |  |
| 23596 | 'OPN3' | mRNA | -0.328572857 | 0.0275781041561937 |  |
| 23607 | 'CD2AP' | mRNA | -0.427199972 | 0.0119024493723628 |  |
| 23617 | 'TSSK2' | mRNA | 6.05419871441419 | 0.0196243839006006 |  |
| 23625 | 'FAM89B' | mRNA | 0.384954581261389 | 0.0170308243518636 |  |
| 23644 | 'EDC4' | mRNA | 0.343271273360963 | 0.0463024232183881 |  |
| 23645 | 'PPP1R15A' | mRNA | 0.346201839789162 | 0.0265193266693923 |  |
| 23767 | 'FLRT3' | mRNA | -0.791842966 | 2.831597298211E-4 |  |
| 24144 | 'TFIP11' | mRNA | 0.38172235207152 | 0.0320100096272963 |  |
| 2475 | 'MTOR' | mRNA | 0.328567342308668 | 0.0221749443852529 |  |
| 2512 | 'FTL' | mRNA | 0.30397807698714 | 0.0015796083140042 |  |
| 2521 | 'FUS' | mRNA | 0.326688463762531 | 0.020584852455856 |  |
| 2533 | 'FYB1' | mRNA | -1.096514504 | 0.0266974560982377 |  |
| 253461 | 'ZBTB38' | mRNA | -0.672862367 | 9.09266171429988E-7 |  |
| 2539 | 'G6PD' | mRNA | 0.275407078452633 | 0.0342528885842414 |  |
| 254065 | 'BRWD3' | mRNA | -0.539633704 | 0.004716143823848 |  |
| 254439 | 'C11orf86' | mRNA | -1.423458806 | 3.197288834564E-4 |  |
| 255743 | 'NPNT' | mRNA | -0.77903288 | 0.0067613167761427 |  |
| 257397 | 'TAB3' | mRNA | -0.469528123 | 0.038978438070652 |  |
| 257407 | 'C2orf72' | mRNA | -0.560116741 | 0.0282739770146606 |  |
| 25814 | 'ATXN10' | mRNA | -0.313046699 | 0.0171220876662438 |  |
| 25847 | 'ANAPC13' | mRNA | -0.364532201 | 0.0498616608093957 |  |
| 25885 | 'POLR1A' | mRNA | 0.306034480982283 | 0.0228293858049928 |  |
| 2589 | 'GALNT1' | mRNA | -0.260127321 | 0.0480557289077568 |  |
| 25897 | 'RNF19A' | mRNA | -0.442022384 | 0.0175636460743385 |  |
| 25909 | 'AHCTF1' | mRNA | -0.420800831 | 0.0134239329754414 |  |
| 259217 | 'HSPA12A' | mRNA | -0.337432181 | 0.0393899458614718 |  |
| 259266 | 'ASPM' | mRNA | -0.963150143 | 7.75760141692E-4 |  |
| 259282 | 'BOD1L1' | mRNA | -0.590985159 | 4.00064389374E-4 |  |
| 25937 | 'WWTR1' | mRNA | -0.261784649 | 0.0387840648593641 |  |
| 25945 | 'NECTIN3' | mRNA | -0.594532389 | 6.78977865998353E-5 |  |
| 25976 | 'TIPARP' | mRNA | 1.21477509570595 | 1.72517827595715E-31 |  |
| 25978 | 'CHMP2B' | mRNA | -0.312093606 | 0.0305480118121378 |  |
| 25980 | 'AAR2' | mRNA | 0.358576614916202 | 0.0218495821029098 |  |
| 25987 | 'TSKU' | mRNA | 0.351422866584565 | 0.0019420324885931 |  |
| 25998 | 'IBTK' | mRNA | -0.457775694 | 0.0010462925649606 |  |
| 26010 | 'SPATS2L' | mRNA | -0.538785457 | 2.77564153169614E-5 |  |
| 26019 | 'UPF2' | mRNA | -0.442942479 | 0.0326023513766073 |  |
| 26046 | 'LTN1' | mRNA | -0.833583162 | 4.93660456483E-4 |  |
| 26054 | 'SENP6' | mRNA | -0.5421555 | 0.0043241263451214 |  |
| 26064 | 'RAI14' | mRNA | -0.666854664 | 5.20237648863249E-6 |  |
| 26100 | 'WIPI2' | mRNA | 0.346489351502704 | 0.0075232977996108 |  |
| 26154 | 'ABCA12' | mRNA | -0.57190154 | 8.78768672431882E-5 |  |
| 26156 | 'RSL1D1' | mRNA | -0.40383763 | 0.0031170893651131 |  |
| 261729 | 'STEAP2' | mRNA | -0.559796949 | 5.760028895075E-4 |  |
| 26173 | 'INTS1' | mRNA | 0.369854355569036 | 0.0122381900309707 |  |
| 262 | 'AMD1' | mRNA | -0.818897031 | 4.10810186820346E-12 |  |
| 26227 | 'PHGDH' | mRNA | 0.599491289188245 | 0.0111998665132736 |  |
| 26246 | 'OR2L2' | mRNA | -1.756973489 | 0.0017244761653342 |  |
| 26267 | 'FBXO10' | mRNA | 0.638083592840616 | 0.0047784926979756 |  |
| 26278 | 'SACS' | mRNA | -0.572695122 | 0.0050457303677131 |  |
| 2629 | 'GBA' | mRNA | 0.314911780133788 | 0.0308173812630189 |  |
| 26298 | 'EHF' | mRNA | -0.867641429 | 2.02436704516121E-9 |  |
| 2635 | 'GBP3' | mRNA | -0.496001975 | 0.0428174707314473 |  |
| 26470 | 'SEZ6L2' | mRNA | 0.381523571127152 | 0.0213051761538143 |  |
| 26586 | 'CKAP2' | mRNA | -0.545126131 | 0.0161835695182806 |  |
| 266740 | 'MAGEA2B' | mRNA | -6.647210426 | 0.0038474771038137 |  |
| 26762 | 'HAVCR1' | mRNA | -0.540394415 | 3.57205509122545E-5 |  |
| 2683 | 'B4GALT1' | mRNA | -0.26391414 | 0.0228293858049928 |  |
| 27000 | 'DNAJC2' | mRNA | -0.804513123 | 0.001511963553624 |  |
| 27111 | 'SDCBP2' | mRNA | -0.626824225 | 0.0439141361448132 |  |
| 27113 | 'BBC3' | mRNA | 0.352297924662624 | 0.0268330842471809 |  |
| 27115 | 'PDE7B' | mRNA | -0.673252161 | 0.0021092415741258 |  |
| 27154 | 'BRPF3' | mRNA | 0.413842565848601 | 0.0356892417726682 |  |
| 2720 | 'GLB1' | mRNA | 0.318091771505384 | 0.0356427013826088 |  |
| 27230 | 'SERP1' | mRNA | -0.320348823 | 0.0145680529522045 |  |
| 27245 | 'AHDC1' | mRNA | 0.451614005318994 | 0.0213055398646055 |  |
| 27284 | 'SULT1B1' | mRNA | -1.333635897 | 0.0013005917368206 |  |
| 27332 | 'ZNF638' | mRNA | -0.51204128 | 0.002248709213677 |  |
| 27346 | 'TMEM97' | mRNA | -0.624495315 | 5.27682609366533E-8 |  |
| 27348 | 'TOR1B' | mRNA | 0.418725445873668 | 0.0368620864244849 |  |
| 2745 | 'GLRX' | mRNA | -0.464013205 | 0.0148797945020208 |  |
| 2771 | 'GNAI2' | mRNA | 0.349263531754274 | 0.0116463662488314 |  |
| 2776 | 'GNAQ' | mRNA | -0.377638321 | 0.016291121976807 |  |
| 2803 | 'GOLGA4' | mRNA | -1.06274699 | 6.091760678679651E-10 |  |
| 2804 | 'GOLGB1' | mRNA | -0.688353625 | 6.66493833804414E-7 |  |
| 282809 | 'POC1B' | mRNA | -0.537093412 | 0.0171658236512854 |  |
| 283 | 'ANG' | mRNA | -0.766767195 | 0.0294232131395639 |  |
| 283149 | 'BCL9L' | mRNA | 0.371145318228756 | 0.0027447592258514 |  |
| 283450 | 'HECTD4' | mRNA | 0.386012971626104 | 0.0087130683094335 |  |
| 283638 | 'CEP170B' | mRNA | 0.298439678492943 | 0.0281792285450662 |  |
| 284021 | 'MILR1' | mRNA | -1.344654996 | 0.0265360990098264 |  |
| 285513 | 'GPRIN3' | mRNA | -0.88518852 | 3.5402952203342803E-9 |  |
| 285672 | 'SREK1IP1' | mRNA | -0.668535829 | 0.0319254586342733 |  |
| 286144 | 'TRIQK' | mRNA | -0.657986536 | 0.0302824983612611 |  |
| 2872 | 'MKNK2' | mRNA | 0.309626273847842 | 0.0337848949119242 |  |
| 2877 | 'GPX2' | mRNA | -0.304986381 | 0.0097771579468838 |  |
| 288 | 'ANK3' | mRNA | -0.513462378 | 0.0424674696382484 |  |
| 2889 | 'RAPGEF1' | mRNA | 0.396149794725833 | 0.0041159487906105 |  |
| 2896 | 'GRN' | mRNA | 0.312025571148682 | 0.0299533376723855 |  |
| 28981 | 'IFT81' | mRNA | -0.497508091 | 0.0249443698486436 |  |
| 28984 | 'RGCC' | mRNA | -1.024160653 | 0.0152729023361431 |  |
| 29028 | 'ATAD2' | mRNA | -0.627629098 | 0.0055936900506835 |  |
| 2907 | 'GRINA' | mRNA | 0.343401735129126 | 0.0093543661046839 |  |
| 29103 | 'DNAJC15' | mRNA | -1.527701609 | 0.0088465108433248 |  |
| 2931 | 'GSK3A' | mRNA | 0.337357911079473 | 0.0140304145842682 |  |
| 2972 | 'BRF1' | mRNA | 0.38087475557186 | 0.0258059197726114 |  |
| 2975 | 'GTF3C1' | mRNA | 0.387648861289368 | 0.0015004472514661 |  |
| 29761 | 'USP25' | mRNA | -0.788636264 | 7.07410048124023E-5 |  |
| 29766 | 'TMOD3' | mRNA | -0.265406231 | 0.0372206014505225 |  |
| 29785 | 'CYP2S1' | mRNA | 0.715931196561719 | 3.5402952203342803E-9 |  |
| 29882 | 'ANAPC2' | mRNA | 0.443874789812884 | 0.032275527805354 |  |
| 29890 | 'RBM15B' | mRNA | 0.339888126739125 | 0.0143337118352532 |  |
| 29923 | 'HILPDA' | mRNA | -0.469116994 | 0.018812898589796 |  |
| 29948 | 'OSGIN1' | mRNA | 0.578867543852804 | 1.068807978588E-4 |  |
| 29950 | 'SERTAD1' | mRNA | 0.58177578427264 | 5.653688697745E-4 |  |
| 29957 | 'SLC25A24' | mRNA | 0.363915186955517 | 0.0464727803406595 |  |
| 29959 | 'NRBP1' | mRNA | 0.347295661267604 | 0.0051989208325824 |  |
| 29994 | 'BAZ2B' | mRNA | -0.692737922 | 0.019009377410143 |  |
| 301 | 'ANXA1' | mRNA | -0.635245465 | 9.46323731728467E-7 |  |
| 3026 | 'HABP2' | mRNA | -1.263586505 | 1.749562715593E-4 |  |
| 3038 | 'HAS3' | mRNA | -0.430965187 | 0.0033741768690167 |  |
| 3054 | 'HCFC1' | mRNA | 0.379220599320474 | 0.003859567671292 |  |
| 306 | 'ANXA3' | mRNA | -0.36543043 | 0.0110581233211182 |  |
| 3064 | 'HTT' | mRNA | 0.340653772561635 | 0.0108278185284957 |  |
| 3075 | 'CFH' | mRNA | -0.528200991 | 2.09461611019849E-5 |  |
| 30827 | 'CXXC1' | mRNA | 0.430246167240692 | 0.0411731616933636 |  |
| 30836 | 'DNTTIP2' | mRNA | -0.689937858 | 1.232654646457E-4 |  |
| 3084 | 'NRG1' | mRNA | -0.406560886 | 0.0021006890319041 |  |
| 30844 | 'EHD4' | mRNA | 0.447268044134828 | 2.096134206834E-4 |  |
| 30846 | 'EHD2' | mRNA | 0.348193954767932 | 0.0051649183358674 |  |
| 3091 | 'HIF1A' | mRNA | -0.799738094 | 3.62509378519813E-19 |  |
| 3098 | 'HK1' | mRNA | 0.368267003093322 | 0.0113301414288699 |  |
| 3105 | 'HLA-A' | mRNA | 0.353606938984935 | 0.0128220679821831 |  |
| 3109 | 'HLA-DMB' | mRNA | -0.979293082 | 2.03657379640637E-9 |  |
| 312 | 'ANXA13' | mRNA | -0.872876142 | 4.32217717278308E-12 |  |
| 3133 | 'HLA-E' | mRNA | 0.313731641979926 | 0.0143854412505232 |  |
| 3151 | 'HMGN2' | mRNA | 0.37553177975331 | 0.0013007168501735 |  |
| 3156 | 'HMGCR' | mRNA | -0.450499125 | 3.033771953966E-4 |  |
| 3157 | 'HMGCS1' | mRNA | -0.635661411 | 4.405288790300609E-11 |  |
| 316 | 'AOX1' | mRNA | -1.252936881 | 1.068807978588E-4 |  |
| 324 | 'APC' | mRNA | -0.752010842 | 0.00123372417459 |  |
| 3248 | 'HPGD' | mRNA | -0.460293787 | 0.003202944148958 |  |
| 3303 | 'HSPA1A' | mRNA | 0.340434922722581 | 0.0283795441960642 |  |
| 3304 | 'HSPA1B' | mRNA | 0.416380379896937 | 6.156283713164E-4 |  |
| 3320 | 'HSP90AA1' | mRNA | -0.309584944 | 0.0102573577786716 |  |
| 3371 | 'TNC' | mRNA | -1.21897887 | 0.0086437251518123 |  |
| 3373 | 'HYAL1' | mRNA | -0.778966177 | 0.0044226310664223 |  |
| 339324 | 'ZNF260' | mRNA | -0.639606159 | 0.034483277371679 |  |
| 3397 | 'ID1' | mRNA | 0.423749508394398 | 8.03209713916402E-5 |  |
| 3398 | 'ID2' | mRNA | 0.557985369085053 | 3.43824486296583E-5 |  |
| 3400 | 'ID4' | mRNA | 0.485084849865237 | 0.0444116190000999 |  |
| 341 | 'APOC1' | mRNA | -0.628440835 | 0.0320273322414166 |  |
| 3454 | 'IFNAR1' | mRNA | -0.470002307 | 0.0054883881839277 |  |
| 345557 | 'PLCXD3' | mRNA | -1.516647884 | 8.96486913489577E-8 |  |
| 347902 | 'AMIGO2' | mRNA | -0.460820727 | 2.82960869836856E-5 |  |
| 3482 | 'IGF2R' | mRNA | 0.27129029237739 | 0.0200741380650661 |  |
| 348262 | 'MCRIP1' | mRNA | 0.44687884302008 | 0.0223907690693749 |  |
| 3486 | 'IGFBP3' | mRNA | -0.630981137 | 2.0992783629738398E-12 |  |
| 348654 | 'GEN1' | mRNA | -0.88403495 | 0.0256785419546671 |  |
| 3572 | 'IL6ST' | mRNA | -0.897616868 | 1.51886703610254E-12 |  |
| 360 | 'AQP3' | mRNA | 0.462744971704469 | 1.239604332774E-4 |  |
| 3625 | 'INHBB' | mRNA | -0.309443481 | 0.0397825808050592 |  |
| 3638 | 'INSIG1' | mRNA | -0.436170299 | 0.0098294451003235 |  |
| 3646 | 'EIF3E' | mRNA | -0.352813065 | 0.018812898589796 |  |
| 3655 | 'ITGA6' | mRNA | -0.373738278 | 0.0016394256941013 |  |
| 3659 | 'IRF1' | mRNA | 0.373546343427673 | 0.0372829879121968 |  |
| 3688 | 'ITGB1' | mRNA | -0.271412038 | 0.0093513092326114 |  |
| 3694 | 'ITGB6' | mRNA | -1.87306018 | 0.002308404843124 |  |
| 3696 | 'ITGB8' | mRNA | -0.870865076 | 0.0252446949012392 |  |
| 3698 | 'ITIH2' | mRNA | -1.01287855 | 5.64975625266012E-9 |  |
| 3727 | 'JUND' | mRNA | 0.331281308916627 | 0.0125746876486468 |  |
| 374393 | 'FAM111B' | mRNA | -1.373702787 | 0.0082868753219497 |  |
| 375035 | 'SFT2D2' | mRNA | -0.401210381 | 0.0061240855402526 |  |
| 375346 | 'STIMATE' | mRNA | 0.457155100799176 | 0.0265094301622729 |  |
| 375743 | 'PTAR1' | mRNA | -0.482081288 | 0.0211962067744015 |  |
| 3773 | 'KCNJ16' | mRNA | -1.053785549 | 2.38284621400923E-6 |  |
| 3796 | 'KIF2A' | mRNA | -0.439864455 | 0.0087845413965365 |  |
| 3799 | 'KIF5B' | mRNA | -0.350630707 | 0.0110580446806872 |  |
| 3841 | 'KPNA5' | mRNA | -0.816911903 | 0.0095657058164638 |  |
| 3842 | 'TNPO1' | mRNA | -0.321559702 | 0.0111717458819998 |  |
| 3851 | 'KRT4' | mRNA | -1.212157899 | 1.3462493542046301E-20 |  |
| 3853 | 'KRT6A' | mRNA | -3.290334399 | 1.17997169358206E-5 |  |
| 3866 | 'KRT15' | mRNA | -0.526291403 | 0.0139907611445156 |  |
| 387882 | 'C12orf75' | mRNA | -0.349355197 | 0.0277793000571612 |  |
| 389136 | 'VGLL3' | mRNA | -0.893210495 | 0.0031965719045706 |  |
| 389177 | 'TMEM212' | mRNA | -1.176993316 | 0.0091079418538916 |  |
| 3895 | 'KTN1' | mRNA | -0.798276655 | 2.2410534136559E-7 |  |
| 3897 | 'L1CAM' | mRNA | 0.860604527531878 | 0.0103425961542807 |  |
| 39 | 'ACAT2' | mRNA | -0.438260196 | 0.0087195531762666 |  |
| 3911 | 'LAMA5' | mRNA | 0.276104273214447 | 0.0286897336069113 |  |
| 3913 | 'LAMB2' | mRNA | 0.453707123157637 | 4.132965593865E-4 |  |
| 3916 | 'LAMP1' | mRNA | 0.305637199670079 | 0.0155719451754604 |  |
| 3918 | 'LAMC2' | mRNA | -0.419391231 | 6.785443888392E-4 |  |
| 3939 | 'LDHA' | mRNA | -0.259498481 | 0.0338063453582558 |  |
| 394 | 'ARHGAP5' | mRNA | -0.626547481 | 5.04922139619158E-5 |  |
| 3959 | 'LGALS3BP' | mRNA | 0.330135321205376 | 0.0090987632864372 |  |
| 3964 | 'LGALS8' | mRNA | -0.450380145 | 0.0114268203945122 |  |
| 3983 | 'ABLIM1' | mRNA | -0.578612124 | 2.4417405869880503E-9 |  |
| 3992 | 'FADS1' | mRNA | -0.437926535 | 3.38889634370094E-5 |  |
| 3996 | 'LLGL1' | mRNA | 0.382595306369007 | 0.0309674062240697 |  |
| 400745 | 'SH2D5' | mRNA | -0.767671819 | 0.003161853254262 |  |
| 4026 | 'LPP' | mRNA | -0.635191324 | 4.37623678689424E-6 |  |
| 4037 | 'LRP3' | mRNA | 0.385361675261906 | 0.0110580446806872 |  |
| 4041 | 'LRP5' | mRNA | 0.272131038778935 | 0.0490435307252901 |  |
| 4071 | 'TM4SF1' | mRNA | -0.708733263 | 9.511011829819191E-9 |  |
| 408 | 'ARRB1' | mRNA | -0.338657434 | 0.0207261846103461 |  |
| 408050 | 'NOMO3' | mRNA | 0.383398111482324 | 0.0019966169352981 |  |
| 4097 | 'MAFG' | mRNA | 0.344465447165065 | 0.0100990409274723 |  |
| 4128 | 'MAOA' | mRNA | -0.961784557 | 0.0130153559879275 |  |
| 4130 | 'MAP1A' | mRNA | 0.880712516409442 | 0.0360703108819807 |  |
| 4131 | 'MAP1B' | mRNA | -0.454625522 | 1.4115608189971E-5 |  |
| 414 | 'ARSD' | mRNA | 0.379741075230146 | 0.0046175340181869 |  |
| 4193 | 'MDM2' | mRNA | -0.340089648 | 0.0135625471450858 |  |
| 4245 | 'MGAT1' | mRNA | 0.299979879128733 | 0.0212283294967425 |  |
| 4254 | 'KITLG' | mRNA | -0.603277374 | 2.390102225997E-4 |  |
| 4286 | 'MITF' | mRNA | -0.720639681 | 0.001179529195586 |  |
| 4289 | 'MKLN1' | mRNA | -0.373381855 | 0.0400836827132725 |  |
| 4343 | 'MOV10' | mRNA | 0.417130259940029 | 0.0111717458819998 |  |
| 444 | 'ASPH' | mRNA | -0.226168577 | 0.0414868820070901 |  |
| 444882 | 'IGFL4' | mRNA | -1.297365193 | 5.67132749201927E-5 |  |
| 4586 | 'MUC5AC' | mRNA | -0.822033678 | 1.2703321681525801E-10 |  |
| 4627 | 'MYH9' | mRNA | 0.395725339510301 | 2.38548733232E-4 |  |
| 4650 | 'MYO9B' | mRNA | 0.338317398366879 | 0.0465109514247257 |  |
| 4666 | 'NACA' | mRNA | -0.304188088 | 0.021176158988652 |  |
| 4680 | 'CEACAM6' | mRNA | -0.315929723 | 0.0034090428144693 |  |
| 4683 | 'NBN' | mRNA | -0.712987387 | 1.660206286258E-4 |  |
| 4734 | 'NEDD4' | mRNA | -0.396541153 | 0.0185614203129305 |  |
| 4756 | 'NEO1' | mRNA | 0.3396809348217 | 0.0077800173668222 |  |
| 4758 | 'NEU1' | mRNA | 0.441091314295545 | 6.207717205337E-4 |  |
| 4774 | 'NFIA' | mRNA | -0.766262353 | 0.0079885522570443 |  |
| 4780 | 'NFE2L2' | mRNA | -0.521362651 | 7.703685378666E-4 |  |
| 4784 | 'NFIX' | mRNA | 0.490024839854156 | 0.019009377410143 |  |
| 481 | 'ATP1B1' | mRNA | -0.288915872 | 0.0241694814699707 |  |
| 4811 | 'NID1' | mRNA | 1.81472825578883 | 0.0041159487906105 |  |
| 4851 | 'NOTCH1' | mRNA | 0.615520896377996 | 2.096134206834E-4 |  |
| 486 | 'FXYD2' | mRNA | -0.322122281 | 0.0235148797641622 |  |
| 488 | 'ATP2A2' | mRNA | 0.251670891525571 | 0.018812898589796 |  |
| 4883 | 'NPR3' | mRNA | -1.075698578 | 0.0140185810794737 |  |
| 4897 | 'NRCAM' | mRNA | -0.304530642 | 0.0111717458819998 |  |
| 490 | 'ATP2B1' | mRNA | -0.42502007 | 2.609796168292E-4 |  |
| 4907 | 'NT5E' | mRNA | -0.403085765 | 0.0300710375813221 |  |
| 4916 | 'NTRK3' | mRNA | -0.833984016 | 4.33262863051117E-6 |  |
| 4922 | 'NTS' | mRNA | -0.449916579 | 0.0045659560190901 |  |
| 4924 | 'NUCB1' | mRNA | 0.277094147482758 | 0.0430331718428756 |  |
| 4925 | 'NUCB2' | mRNA | -0.684976476 | 0.0015245065095642 |  |
| 4926 | 'NUMA1' | mRNA | 0.370209303269858 | 0.0087426320055654 |  |
| 4953 | 'ODC1' | mRNA | 0.263983827691691 | 0.0476980262706315 |  |
| 4976 | 'OPA1' | mRNA | -0.583185804 | 1.622133145355E-4 |  |
| 5000 | 'ORC4' | mRNA | -0.584036671 | 0.0022863914942807 |  |
| 5021 | 'OXTR' | mRNA | -1.472067433 | 3.193027236924E-4 |  |
| 50626 | 'CYHR1' | mRNA | 0.425799494820815 | 0.014097327218718 |  |
| 50807 | 'ASAP1' | mRNA | -0.493461992 | 2.05343203994226E-5 |  |
| 5108 | 'PCM1' | mRNA | -0.764788791 | 3.63017517088456E-7 |  |
| 51090 | 'PLLP' | mRNA | -0.858141196 | 0.0012858999227864 |  |
| 51092 | 'SIDT2' | mRNA | 0.480138249822628 | 0.0299533376723855 |  |
| 51181 | 'DCXR' | mRNA | 0.482770518145873 | 0.0087644487982718 |  |
| 51196 | 'PLCE1' | mRNA | -0.651259183 | 2.831597298211E-4 |  |
| 5122 | 'PCSK1' | mRNA | -1.116121878 | 0.0048739893223923 |  |
| 51278 | 'IER5' | mRNA | 0.322778481219976 | 0.0116866896844511 |  |
| 51290 | 'ERGIC2' | mRNA | -0.613637191 | 0.0018676947805794 |  |
| 51307 | 'FAM53C' | mRNA | 0.422377334060769 | 0.0031342781549301 |  |
| 51313 | 'GASK1B' | mRNA | -0.409347701 | 0.0145680529522045 |  |
| 5137 | 'PDE1C' | mRNA | -1.48910067 | 2.6118267259E-4 |  |
| 5139 | 'PDE3A' | mRNA | -0.556271808 | 3.91557169468073E-7 |  |
| 5142 | 'PDE4B' | mRNA | -0.413424159 | 0.0221149291858299 |  |
| 5144 | 'PDE4D' | mRNA | -0.33393798 | 0.0200741380650661 |  |
| 51449 | 'PCYOX1' | mRNA | 0.265492083645431 | 0.0404481716508665 |  |
| 51474 | 'LIMA1' | mRNA | -0.458456231 | 0.0047298082903431 |  |
| 51575 | 'ESF1' | mRNA | -1.277274257 | 8.96486913489577E-8 |  |
| 51602 | 'NOP58' | mRNA | -0.802580633 | 4.44101945743051E-6 |  |
| 51684 | 'SUFU' | mRNA | 0.454069573436574 | 0.0162140646551072 |  |
| 51704 | 'GPRC5B' | mRNA | -0.570916836 | 5.760028895075E-4 |  |
| 5174 | 'PDZK1' | mRNA | -1.55188048 | 2.379178848604E-4 |  |
| 51755 | 'CDK12' | mRNA | 0.296211472059941 | 0.0399820538055924 |  |
| 51773 | 'RSF1' | mRNA | -0.562899582 | 0.0053761457418083 |  |
| 51809 | 'GALNT7' | mRNA | -0.340187334 | 0.0114731172663439 |  |
| 5209 | 'PFKFB3' | mRNA | 0.246526439012823 | 0.0369229011501982 |  |
| 5257 | 'PHKB' | mRNA | -0.381107183 | 0.0140945810867352 |  |
| 5286 | 'PIK3C2A' | mRNA | -0.789845384 | 4.08888098974272E-8 |  |
| 5295 | 'PIK3R1' | mRNA | -0.484786771 | 0.0229053166953737 |  |
| 5310 | 'PKD1' | mRNA | 0.379774172363318 | 0.002286705392427 |  |
| 5321 | 'PLA2G4A' | mRNA | -0.569481265 | 1.087965944978E-4 |  |
| 5332 | 'PLCB4' | mRNA | -0.768478522 | 0.0047971238302106 |  |
| 53349 | 'ZFYVE1' | mRNA | 0.452298879406478 | 0.0107470454162894 |  |
| 5335 | 'PLCG1' | mRNA | 0.332770029076887 | 0.018812898589796 |  |
| 5336 | 'PLCG2' | mRNA | 0.701671788779529 | 0.0213055398646055 |  |
| 5338 | 'PLD2' | mRNA | 0.515564932682121 | 0.0035174669257449 |  |
| 5339 | 'PLEC' | mRNA | 0.501142568115949 | 3.2699973601968E-6 |  |
| 5357 | 'PLS1' | mRNA | -0.586667993 | 8.10232368556428E-6 |  |
| 5361 | 'PLXNA1' | mRNA | 0.338147839462361 | 0.021176158988652 |  |
| 5362 | 'PLXNA2' | mRNA | -0.521553856 | 0.0075232977996108 |  |
| 537 | 'ATP6AP1' | mRNA | 0.380570083402284 | 0.0043788316054442 |  |
| 540 | 'ATP7B' | mRNA | 0.535719965578516 | 0.0148818206553439 |  |
| 54101 | 'RIPK4' | mRNA | 0.624595023563691 | 7.12379061169552E-6 |  |
| 5411 | 'PNN' | mRNA | -0.570472389 | 0.0040432684749684 |  |
| 5428 | 'POLG' | mRNA | 0.332128705903337 | 0.0192311073737422 |  |
| 5430 | 'POLR2A' | mRNA | 0.694470815988668 | 1.07631179597902E-12 |  |
| 54431 | 'DNAJC10' | mRNA | -0.553924777 | 5.93562435985689E-6 |  |
| 54443 | 'ANLN' | mRNA | -0.492704699 | 0.0209253033930458 |  |
| 5445 | 'PON2' | mRNA | -0.30084004 | 0.0273883287729532 |  |
| 54462 | 'CCSER2' | mRNA | -0.454850081 | 0.0461020594018806 |  |
| 54472 | 'TOLLIP' | mRNA | 0.317633555005596 | 0.0257280140311783 |  |
| 54474 | 'KRT20' | mRNA | -0.947803878 | 1.94158532689432E-5 |  |
| 54476 | 'RNF216' | mRNA | 0.290750812669611 | 0.0489640764419677 |  |
| 54477 | 'PLEKHA5' | mRNA | -0.498443298 | 0.0457687662572946 |  |
| 54532 | 'USP53' | mRNA | -0.861686478 | 4.44101945743051E-6 |  |
| 54537 | 'SHLD2' | mRNA | -0.653443332 | 7.356315828746E-4 |  |
| 54550 | 'NECAB2' | mRNA | 1.14282989311193 | 0.0336297023987917 |  |
| 54577 | 'UGT1A7' | mRNA | -0.713463757 | 8.03209713916402E-5 |  |
| 54583 | 'EGLN1' | mRNA | -0.427410611 | 1.31770230127E-4 |  |
| 546 | 'ATRX' | mRNA | -0.773740539 | 5.43191120544697E-7 |  |
| 54662 | 'TBC1D13' | mRNA | 0.461052013588045 | 0.0045729597291181 |  |
| 54664 | 'TMEM106B' | mRNA | -0.495092259 | 2.09461611019849E-5 |  |
| 54681 | 'P4HTM' | mRNA | 0.38712993856747 | 0.0420427532762383 |  |
| 54704 | 'PDP1' | mRNA | 0.557766774096019 | 0.0319050572584707 |  |
| 5476 | 'CTSA' | mRNA | 0.316933162852247 | 0.0165177383619913 |  |
| 54801 | 'HAUS6' | mRNA | -0.664631826 | 0.00123372417459 |  |
| 54806 | 'AHI1' | mRNA | -1.0295553 | 0.0116463662488314 |  |
| 54822 | 'TRPM7' | mRNA | -0.43219553 | 0.0140945810867352 |  |
| 54832 | 'VPS13C' | mRNA | -0.408164971 | 0.0047971238302106 |  |
| 54849 | 'DEF8' | mRNA | 0.303765464499224 | 0.0465330741617071 |  |
| 54852 | 'PAQR5' | mRNA | -0.577568162 | 0.0047971238302106 |  |
| 54862 | 'CC2D1A' | mRNA | 0.378187013639564 | 0.0393048947371375 |  |
| 54873 | 'PALMD' | mRNA | -1.129284704 | 0.0019939505027497 |  |
| 54880 | 'BCOR' | mRNA | 0.35152950531682 | 0.0257280140311783 |  |
| 54906 | 'TASOR2' | mRNA | -0.615416019 | 1.27314754895533E-6 |  |
| 54921 | 'CHTF8' | mRNA | 0.329447630218142 | 0.0223523718474546 |  |
| 54953 | 'ODR4' | mRNA | -0.626350896 | 0.0257280140311783 |  |
| 55023 | 'PHIP' | mRNA | -0.658582716 | 0.0010310428793177 |  |
| 55066 | 'PDPR' | mRNA | 0.369026617460551 | 0.0098727645323173 |  |
| 55075 | 'UACA' | mRNA | -0.768017149 | 0.001271711821479 |  |
| 55088 | 'CCDC186' | mRNA | -0.691542479 | 0.0280156272914277 |  |
| 55109 | 'AGGF1' | mRNA | -0.427364331 | 0.0435104356327556 |  |
| 55151 | 'TMEM38B' | mRNA | 0.600803564399494 | 0.0294121014258497 |  |
| 55183 | 'RIF1' | mRNA | -0.775292238 | 4.090902024198E-4 |  |
| 55195 | 'CCDC198' | mRNA | -1.223704999 | 0.0054883881839277 |  |
| 55196 | 'RESF1' | mRNA | -1.037660565 | 2.710683323864E-4 |  |
| 55205 | 'ZNF532' | mRNA | -0.455340522 | 0.0036978058210198 |  |
| 55218 | 'EXD2' | mRNA | 0.353869352187038 | 0.0487385832649764 |  |
| 55226 | 'NAT10' | mRNA | 0.282375476507699 | 0.0491002840758559 |  |
| 55244 | 'SLC47A1' | mRNA | 0.631746383199728 | 2.543462606518E-4 |  |
| 5528 | 'PPP2R5D' | mRNA | 0.38616933066285 | 0.0320273322414166 |  |
| 55313 | 'CPPED1' | mRNA | -0.386382219 | 0.0113270292571697 |  |
| 55320 | 'MIS18BP1' | mRNA | -0.811258371 | 0.0252319662969974 |  |
| 55351 | 'STK32B' | mRNA | 0.391767727284463 | 0.0060501330564927 |  |
| 554282 | 'FAM72C' | mRNA | -6.60677159 | 0.0048297000609222 |  |
| 55588 | 'MED29' | mRNA | 0.276705687772311 | 0.0325334086898958 |  |
| 55591 | 'VEZT' | mRNA | -0.467065842 | 0.0011936020412264 |  |
| 55593 | 'OTUD5' | mRNA | 0.32021988574012 | 0.0280156272914277 |  |
| 55605 | 'KIF21A' | mRNA | -0.561498531 | 0.0051745101488686 |  |
| 55612 | 'FERMT1' | mRNA | -0.350020089 | 0.0256846607646648 |  |
| 55616 | 'ASAP3' | mRNA | 0.512845121886892 | 0.0413050577978857 |  |
| 55660 | 'PRPF40A' | mRNA | -0.397412877 | 0.031325083187405 |  |
| 55662 | 'HIF1AN' | mRNA | 0.316315630153377 | 0.0133084851111497 |  |
| 55665 | 'URGCP' | mRNA | 0.414785461885434 | 0.0067217487724813 |  |
| 55667 | 'DENND4C' | mRNA | -0.424322982 | 0.0022890210575021 |  |
| 5567 | 'PRKACB' | mRNA | -0.514629955 | 0.0108748813296753 |  |
| 55681 | 'SCYL2' | mRNA | -0.466139521 | 0.0047971238302106 |  |
| 55714 | 'TENM3' | mRNA | 0.286198955618544 | 0.0192869264247137 |  |
| 55745 | 'AP5M1' | mRNA | -0.468314176 | 0.0175636460743385 |  |
| 55749 | 'CCAR1' | mRNA | -0.528380169 | 0.0012490854643822 |  |
| 55785 | 'FGD6' | mRNA | -0.777260405 | 4.22506426216116E-8 |  |
| 55790 | 'CSGALNACT1' | mRNA | -0.394767355 | 0.0075232977996108 |  |
| 55808 | 'ST6GALNAC1' | mRNA | -0.747731808 | 0.0237831308414658 |  |
| 55810 | 'FOXJ2' | mRNA | 0.452208755235804 | 0.0060513283307558 |  |
| 55814 | 'BDP1' | mRNA | -0.785604154 | 7.69116606027984E-6 |  |
| 5586 | 'PKN2' | mRNA | -0.496667844 | 0.0254900346655763 |  |
| 55890 | 'GPRC5C' | mRNA | 0.413762052685434 | 0.0334530511128644 |  |
| 55898 | 'UNC45A' | mRNA | 0.442096218919565 | 0.0027625732823531 |  |
| 55904 | 'KMT2E' | mRNA | -0.516796137 | 0.0045659560190901 |  |
| 55930 | 'MYO5C' | mRNA | -0.433278121 | 0.0208368182628777 |  |
| 55970 | 'GNG12' | mRNA | -0.306210335 | 0.0248488178564401 |  |
| 56108 | 'PCDHGA7' | mRNA | 1.09675294031233 | 0.0487385832649764 |  |
| 56204 | 'FAM214A' | mRNA | -0.509780301 | 0.0209253033930458 |  |
| 56257 | 'MEPCE' | mRNA | 0.460105596806864 | 0.0047971238302106 |  |
| 56261 | 'GPCPD1' | mRNA | -0.534218505 | 1.29268330754856E-5 |  |
| 56288 | 'PARD3' | mRNA | -0.482225211 | 1.58268945694017E-5 |  |
| 56521 | 'DNAJC12' | mRNA | -0.792299576 | 2.704409205571E-4 |  |
| 5654 | 'HTRA1' | mRNA | 0.359242122690673 | 0.0143310854969034 |  |
| 56667 | 'MUC13' | mRNA | -0.371857129 | 0.0093543661046839 |  |
| 56905 | 'C15orf39' | mRNA | 0.442048050763276 | 0.0091339512352858 |  |
| 56913 | 'C1GALT1' | mRNA | -0.395786233 | 0.0366896268917486 |  |
| 56937 | 'PMEPA1' | mRNA | -0.517222398 | 3.60244956627522E-6 |  |
| 56987 | 'BBX' | mRNA | -0.613047039 | 1.310652730131E-4 |  |
| 56994 | 'CHPT1' | mRNA | 0.320061189627569 | 0.0403898265059635 |  |
| 570 | 'BAAT' | mRNA | -0.791976765 | 0.0011462144041949 |  |
| 57104 | 'PNPLA2' | mRNA | 0.55029166816424 | 4.210416252052E-4 |  |
| 57118 | 'CAMK1D' | mRNA | -0.423555464 | 0.0063819482779443 |  |
| 57132 | 'CHMP1B' | mRNA | 0.471994866918541 | 6.997866814027E-4 |  |
| 57169 | 'ZNFX1' | mRNA | 0.323080584680253 | 0.0048370392252474 |  |
| 57181 | 'SLC39A10' | mRNA | -1.046455224 | 3.61563965133338E-10 |  |
| 57187 | 'THOC2' | mRNA | -0.719782952 | 3.65953924096294E-7 |  |
| 57190 | 'SELENON' | mRNA | 0.318184716483177 | 0.0122099876179845 |  |
| 57194 | 'ATP10A' | mRNA | -0.589448298 | 0.0229053166953737 |  |
| 5724 | 'PTAFR' | mRNA | -0.845292198 | 0.0281665003517183 |  |
| 57326 | 'PBXIP1' | mRNA | 0.550641056095738 | 1.811717843309E-4 |  |
| 57456 | 'KIAA1143' | mRNA | -0.657460417 | 0.0038277531459427 |  |
| 57476 | 'GRAMD1B' | mRNA | -0.269264888 | 0.0372120516267916 |  |
| 57479 | 'PRR12' | mRNA | 0.355573392656914 | 0.0196794835038587 |  |
| 57491 | 'AHRR' | mRNA | 1.253964749 | 5.64975625266012E-9 |  |
| 57494 | 'RIMKLB' | mRNA | -0.370774536 | 0.0283795441960642 |  |
| 57509 | 'MTUS1' | mRNA | -1.06796295 | 2.14862051655754E-28 |  |
| 57521 | 'RPTOR' | mRNA | 0.353134400233862 | 0.0249566960206944 |  |
| 57535 | 'ELAPOR1' | mRNA | -0.848761133 | 0.0230464237366705 |  |
| 57551 | 'TAOK1' | mRNA | -0.525613369 | 4.01156239329168E-5 |  |
| 57562 | 'CEP126' | mRNA | -1.060169345 | 0.0187336683999952 |  |
| 57584 | 'ARHGAP21' | mRNA | -0.542996832 | 2.309273008235E-4 |  |
| 57586 | 'SYT13' | mRNA | -0.788945451 | 3.40125536293932E-8 |  |
| 57634 | 'EP400' | mRNA | 0.332757861030938 | 0.0153058815954771 |  |
| 57658 | 'CALCOCO1' | mRNA | 0.545968038975975 | 0.0039598706983985 |  |
| 57674 | 'RNF213' | mRNA | 0.270650588252204 | 0.0162140646551072 |  |
| 5768 | 'QSOX1' | mRNA | 0.321986067978441 | 0.0223907690693749 |  |
| 57698 | 'SHTN1' | mRNA | -0.310378548 | 0.0371322050618396 |  |
| 57703 | 'CWC22' | mRNA | -0.93652344 | 0.015363764908844 |  |
| 57707 | 'MEAK7' | mRNA | 0.377839032699784 | 0.0051066889713767 |  |
| 57794 | 'SUGP1' | mRNA | 0.420558675354405 | 0.0433910217029745 |  |
| 5782 | 'PTPN12' | mRNA | -0.343722934 | 0.0465330741617071 |  |
| 5783 | 'PTPN13' | mRNA | -0.773452364 | 0.0012488957153502 |  |
| 58497 | 'PRUNE1' | mRNA | 0.435099938181027 | 0.0482526677045067 |  |
| 58506 | 'SCAF1' | mRNA | 0.345506168269782 | 0.0149104129761993 |  |
| 58517 | 'RBM25' | mRNA | -0.499845454 | 1.093381591774E-4 |  |
| 5865 | 'RAB3B' | mRNA | -0.931956573 | 1.26929411323382E-7 |  |
| 5874 | 'RAB27B' | mRNA | -1.176274895 | 5.1084348992861594E-18 |  |
| 5903 | 'RANBP2' | mRNA | -0.490833993 | 2.890207541491E-4 |  |
| 5915 | 'RARB' | mRNA | -0.787681186 | 0.004913207321876 |  |
| 59277 | 'NTN4' | mRNA | 0.473589282007769 | 4.50657076615628E-5 |  |
| 5930 | 'RBBP6' | mRNA | -0.53216066 | 0.0015443531032262 |  |
| 5932 | 'RBBP8' | mRNA | -0.724490367 | 0.0089311962044736 |  |
| 5937 | 'RBMS1' | mRNA | -0.378506391 | 0.0441647088072303 |  |
| 5962 | 'RDX' | mRNA | -0.327529277 | 0.0084705102036965 |  |
| 5966 | 'REL' | mRNA | -0.799899096 | 0.0050437457669036 |  |
| 5976 | 'UPF1' | mRNA | 0.337908228410554 | 0.0188599088223298 |  |
| 5980 | 'REV3L' | mRNA | -0.992946254 | 5.12246201480549E-5 |  |
| 5997 | 'RGS2' | mRNA | -0.753079688 | 0.0111511745125464 |  |
| 602 | 'BCL3' | mRNA | 0.580870683534402 | 1.087965944978E-4 |  |
| 60343 | 'FAM3A' | mRNA | 0.330435178080583 | 0.0403033334723673 |  |
| 6038 | 'RNASE4' | mRNA | -0.669726371 | 0.0027039719569572 |  |
| 6046 | 'BRD2' | mRNA | 0.431932056987083 | 5.1106467482593E-5 |  |
| 6051 | 'RNPEP' | mRNA | 0.285963676153197 | 0.0455430643173471 |  |
| 6059 | 'ABCE1' | mRNA | -0.539167589 | 5.13203928459464E-5 |  |
| 60678 | 'EEFSEC' | mRNA | 0.527364137035831 | 0.0230464237366705 |  |
| 6093 | 'ROCK1' | mRNA | -0.561027271 | 0.0010480047818338 |  |
| 6098 | 'ROS1' | mRNA | -0.94191875 | 5.954004395995E-4 |  |
| 6197 | 'RPS6KA3' | mRNA | -0.376245226 | 0.0040645578621511 |  |
| 6249 | 'CLIP1' | mRNA | -0.44319779 | 5.969006821453E-4 |  |
| 627 | 'BDNF' | mRNA | -1.040555567 | 2.77564153169614E-5 |  |
| 6275 | 'S100A4' | mRNA | -0.271817201 | 0.030041372332842 |  |
| 63035 | 'BCORL1' | mRNA | 0.466297976771427 | 0.0228293858049928 |  |
| 6305 | 'SBF1' | mRNA | 0.337374775279463 | 0.0258231037874763 |  |
| 6307 | 'MSMO1' | mRNA | -0.491944836 | 9.660703713099E-4 |  |
| 6319 | 'SCD' | mRNA | -0.351497315 | 4.013763482011E-4 |  |
| 6337 | 'SCNN1A' | mRNA | -0.469298969 | 0.0100990409274723 |  |
| 6374 | 'CXCL5' | mRNA | -0.258601552 | 0.0223020836039823 |  |
| 63891 | 'RNF123' | mRNA | 0.431483002228451 | 0.0366896268917486 |  |
| 63893 | 'UBE2O' | mRNA | 0.385427358615266 | 0.0196794835038587 |  |
| 64061 | 'TSPYL2' | mRNA | 0.540869996433778 | 0.004716143823848 |  |
| 64093 | 'SMOC1' | mRNA | -0.437343556 | 0.0040975842951292 |  |
| 64097 | 'EPB41L4A' | mRNA | -0.430088161 | 0.0022731933692255 |  |
| 64207 | 'IRF2BPL' | mRNA | 0.610788499960201 | 5.93562435985689E-6 |  |
| 64318 | 'NOC3L' | mRNA | -0.66271772 | 0.0049691662503308 |  |
| 64319 | 'FBRS' | mRNA | 0.306120081757935 | 0.0429100175898696 |  |
| 6446 | 'SGK1' | mRNA | -0.778019677 | 0.0405386984674847 |  |
| 6464 | 'SHC1' | mRNA | 0.279190049995091 | 0.0185614203129305 |  |
| 6468 | 'FBXW4' | mRNA | 0.37217151066737 | 0.0485768450511541 |  |
| 6480 | 'ST6GAL1' | mRNA | -0.576212521 | 0.0171222150781613 |  |
| 64864 | 'RFX7' | mRNA | -0.486555497 | 0.004880869563011 |  |
| 6497 | 'SKI' | mRNA | 0.345541909166219 | 0.0275781041561937 |  |
| 65009 | 'NDRG4' | mRNA | -0.510142576 | 2.09461611019849E-5 |  |
| 65056 | 'GPBP1' | mRNA | -0.442195146 | 0.0143310854969034 |  |
| 65059 | 'RAPH1' | mRNA | -0.551639332 | 0.0073415531544294 |  |
| 6509 | 'SLC1A4' | mRNA | 0.519202918465182 | 0.0413121692598365 |  |
| 6510 | 'SLC1A5' | mRNA | 0.290935063116967 | 0.031013242911788 |  |
| 65123 | 'INTS3' | mRNA | 0.368132176828332 | 0.0162140646551072 |  |
| 651746 | 'ANKRD33B' | mRNA | -0.416394961 | 0.0454390610425998 |  |
| 6520 | 'SLC3A2' | mRNA | 0.569855787559555 | 1.47711478913053E-8 |  |
| 652968 | 'CASTOR1' | mRNA | 0.774177009654052 | 0.0377106930033128 |  |
| 653145 | 'ANXA8' | mRNA | -0.771257409 | 1.59133486365668E-8 |  |
| 6533 | 'SLC6A6' | mRNA | 0.328295418894205 | 0.0035687095549555 |  |
| 6535 | 'SLC6A8' | mRNA | 0.543612655452783 | 5.238712021195E-4 |  |
| 653781 | 'POTEJ' | mRNA | 9.01015133449644 | 3.91557169468073E-7 |  |
| 6542 | 'SLC7A2' | mRNA | -0.380464394 | 0.0380172433849287 |  |
| 6548 | 'SLC9A1' | mRNA | 0.419457967463678 | 0.0294979295646401 |  |
| 6558 | 'SLC12A2' | mRNA | -0.519041135 | 1.35472316153845E-6 |  |
| 6560 | 'SLC12A4' | mRNA | 0.442631071752433 | 7.815350466878E-4 |  |
| 6601 | 'SMARCC2' | mRNA | 0.275502573727808 | 0.0452609092460402 |  |
| 6616 | 'SNAP25' | mRNA | -1.254917225 | 0.0211391555470911 |  |
| 6640 | 'SNTA1' | mRNA | 0.439450472554088 | 0.0440195525160995 |  |
| 6662 | 'SOX9' | mRNA | -0.413503134 | 0.0262571111834666 |  |
| 667 | 'DST' | mRNA | -1.022378381 | 1.47518982502041E-17 |  |
| 6696 | 'SPP1' | mRNA | -0.457632712 | 3.672870842594E-4 |  |
| 6709 | 'SPTAN1' | mRNA | 0.308887185177602 | 0.0061226010060261 |  |
| 6713 | 'SQLE' | mRNA | -0.335752525 | 0.0093513092326114 |  |
| 6731 | 'SRP72' | mRNA | -0.417443726 | 0.0039598706983985 |  |
| 6745 | 'SSR1' | mRNA | -0.538145019 | 1.35472316153845E-6 |  |
| 6747 | 'SSR3' | mRNA | -0.266660578 | 0.0468594233564795 |  |
| 6772 | 'STAT1' | mRNA | 0.29830286049984 | 0.0226357094259698 |  |
| 6781 | 'STC1' | mRNA | -0.815683179 | 8.96486913489577E-8 |  |
| 6785 | 'ELOVL4' | mRNA | 0.673285160947486 | 0.0140687808697782 |  |
| 6786 | 'STIM1' | mRNA | 0.270224586869017 | 0.0437669068160489 |  |
| 6812 | 'STXBP1' | mRNA | 0.409538951484069 | 0.0115381521949064 |  |
| 6818 | 'SULT1A3' | mRNA | -0.576316136 | 9.540756826004E-4 |  |
| 6820 | 'SULT2B1' | mRNA | -0.786152444 | 1.471765892E-4 |  |
| 6829 | 'SUPT5H' | mRNA | 0.291617375952377 | 0.0345130232607765 |  |
| 6830 | 'SUPT6H' | mRNA | 0.298028078589114 | 0.0233139943300451 |  |
| 6904 | 'TBCD' | mRNA | 0.439211602742656 | 0.0019715743261679 |  |
| 6926 | 'TBX3' | mRNA | 0.443471315812994 | 0.0143854412505232 |  |
| 6940 | 'ZNF354A' | mRNA | -0.758689821 | 0.0464727803406595 |  |
| 6947 | 'TCN1' | mRNA | -0.85945251 | 7.75308045959555E-7 |  |
| 7003 | 'TEAD1' | mRNA | -0.661004307 | 3.2647808563585802E-9 |  |
| 7011 | 'TEP1' | mRNA | 0.497242260145665 | 0.0015648378497747 |  |
| 7035 | 'TFPI' | mRNA | -0.446054909 | 0.0206615501317999 |  |
| 7045 | 'TGFBI' | mRNA | 0.275888590890235 | 0.0110580446806872 |  |
| 7048 | 'TGFBR2' | mRNA | -0.291572401 | 0.0135046592832339 |  |
| 7052 | 'TGM2' | mRNA | -0.66712363 | 3.08608806971009E-10 |  |
| 7090 | 'TLE3' | mRNA | 0.537874230012104 | 1.068807978588E-4 |  |
| 7094 | 'TLN1' | mRNA | 0.326175123443327 | 0.0150031465757704 |  |
| 7099 | 'TLR4' | mRNA | -0.756348141 | 0.0150031465757704 |  |
| 7103 | 'TSPAN8' | mRNA | -1.19297704 | 3.243722529495E-4 |  |
| 7104 | 'TM4SF4' | mRNA | -1.3121336 | 2.14862051655754E-28 |  |
| 7110 | 'TMF1' | mRNA | -0.571177291 | 0.019009377410143 |  |
| 7150 | 'TOP1' | mRNA | -0.313067149 | 0.029345384409121 |  |
| 7153 | 'TOP2A' | mRNA | -0.346681865 | 0.0223020836039823 |  |
| 7164 | 'TPD52L1' | mRNA | -0.451965583 | 0.0036978058210198 |  |
| 7168 | 'TPM1' | mRNA | -0.463844091 | 3.44363190488E-4 |  |
| 7171 | 'TPM4' | mRNA | -0.331705246 | 0.0015486488478541 |  |
| 7175 | 'TPR' | mRNA | -0.66797169 | 9.05026889756816E-7 |  |
| 7184 | 'HSP90B1' | mRNA | -0.548367667 | 1.7029165150756701E-9 |  |
| 722 | 'C4BPA' | mRNA | -0.725705079 | 4.19099674443753E-5 |  |
| 7249 | 'TSC2' | mRNA | 0.394587170593578 | 0.0280539708806987 |  |
| 7259 | 'TSPYL1' | mRNA | 0.347262623922366 | 0.0049691662503308 |  |
| 7263 | 'TST' | mRNA | 0.331547331794741 | 0.0331979866455625 |  |
| 7267 | 'TTC3' | mRNA | -0.821421414 | 4.405288790300609E-11 |  |
| 727851 | 'RGPD8' | mRNA | -1.120581603 | 0.0030467365181247 |  |
| 727897 | 'MUC5B' | mRNA | -0.62603263 | 5.2349855854293E-12 |  |
| 728113 | 'ANXA8L1' | mRNA | -0.77212031 | 8.03209713916402E-5 |  |
| 7286 | 'TUFT1' | mRNA | 0.431155967894789 | 0.0054883881839277 |  |
| 7358 | 'UGDH' | mRNA | -0.271087805 | 0.0235148797641622 |  |
| 7402 | 'UTRN' | mRNA | -0.627407751 | 8.4438699004918E-5 |  |
| 7433 | 'VIPR1' | mRNA | 0.75062252600019 | 0.0016053928356211 |  |
| 7461 | 'CLIP2' | mRNA | 0.416781688551922 | 0.0011462144041949 |  |
| 7477 | 'WNT7B' | mRNA | 0.408191356498667 | 9.097940067148E-4 |  |
| 7529 | 'YWHAB' | mRNA | -0.280386029 | 0.0251353281251274 |  |
| 7581 | 'ZNF33A' | mRNA | -0.802043525 | 0.0060663412160856 |  |
| 7617 | 'ZNF66' | mRNA | -1.048901101 | 3.88051917112155E-6 |  |
| 7705 | 'ZNF146' | mRNA | -0.499613791 | 1.0475187732E-4 |  |
| 7706 | 'TRIM25' | mRNA | 0.294705018174406 | 0.0181656847268603 |  |
| 7748 | 'ZNF195' | mRNA | -0.903001588 | 1.087965944978E-4 |  |
| 7750 | 'ZMYM2' | mRNA | -0.492174563 | 0.0181090488366784 |  |
| 7832 | 'BTG2' | mRNA | 0.324802937396218 | 0.0331249659959374 |  |
| 7846 | 'TUBA1A' | mRNA | 0.373576375557733 | 0.0014292782072514 |  |
| 790 | 'CAD' | mRNA | 0.310150311458939 | 0.0452085544769146 |  |
| 79071 | 'ELOVL6' | mRNA | -0.712571928 | 3.59713010250131E-5 |  |
| 7913 | 'DEK' | mRNA | -0.568992481 | 4.090902024198E-4 |  |
| 79137 | 'RETREG2' | mRNA | 0.394525410434146 | 0.0035687095549555 |  |
| 79144 | 'PPDPF' | mRNA | 0.323774956077506 | 0.0283795441960642 |  |
| 7916 | 'PRRC2A' | mRNA | 0.459337580240944 | 8.83668234273185E-5 |  |
| 79170 | 'PRR15L' | mRNA | -1.209681627 | 0.0087117308001336 |  |
| 79184 | 'BRCC3' | mRNA | -0.469628186 | 0.0184086439971063 |  |
| 79413 | 'ZBED2' | mRNA | -1.269894492 | 8.769301362154E-4 |  |
| 79586 | 'CHPF' | mRNA | 0.351334358660879 | 0.0241694814699707 |  |
| 79710 | 'MORC4' | mRNA | -0.368591683 | 0.0053789072930236 |  |
| 79811 | 'SLTM' | mRNA | -0.652841175 | 7.33285793257969E-7 |  |
| 79832 | 'QSER1' | mRNA | -0.593032748 | 2.890207541491E-4 |  |
| 79837 | 'PIP4K2C' | mRNA | 0.343466845091843 | 0.0119024493723628 |  |
| 79838 | 'TMC5' | mRNA | -0.549016711 | 0.0171220876662438 |  |
| 79853 | 'TM4SF20' | mRNA | -0.867460629 | 1.01531996427031E-5 |  |
| 79871 | 'RPAP2' | mRNA | -0.724125429 | 0.0023328888812339 |  |
| 79888 | 'LPCAT1' | mRNA | -0.369142123 | 2.379178848604E-4 |  |
| 79937 | 'CNTNAP3' | mRNA | -0.662361627 | 0.0016907623194356 |  |
| 79960 | 'JADE1' | mRNA | -0.585463647 | 0.00133564884935 |  |
| 800 | 'CALD1' | mRNA | -0.439858926 | 3.235142237036E-4 |  |
| 80018 | 'NAA25' | mRNA | -0.366533866 | 0.0441647088072303 |  |
| 80020 | 'FOXRED2' | mRNA | 0.339361191952992 | 0.0299310325208107 |  |
| 80139 | 'ZNF703' | mRNA | 0.620425826874271 | 0.0012736896924276 |  |
| 80144 | 'FRAS1' | mRNA | -0.396672521 | 0.0149948363050016 |  |
| 80205 | 'CHD9' | mRNA | -0.768307656 | 6.611127224187E-6 |  |
| 8021 | 'NUP214' | mRNA | 0.376686760522634 | 0.018810387692448 |  |
| 8030 | 'CCDC6' | mRNA | -0.343325423 | 0.0110580446806872 |  |
| 80310 | 'PDGFD' | mRNA | -0.671693418 | 3.51558534623867E-5 |  |
| 80323 | 'CCDC68' | mRNA | -0.832589091 | 0.0215501798205306 |  |
| 8073 | 'PTP4A2' | mRNA | -0.352023816 | 0.0080475744353161 |  |
| 80760 | 'ITIH5' | mRNA | -0.915271058 | 1.19239944174464E-7 |  |
| 8085 | 'KMT2D' | mRNA | 0.401999442850575 | 8.132269400651E-4 |  |
| 8140 | 'SLC7A5' | mRNA | 0.703567951014769 | 1.30940212616003E-16 |  |
| 81490 | 'PTDSS2' | mRNA | 0.347346121917382 | 0.0495470755215261 |  |
| 81537 | 'SGPP1' | mRNA | 0.363568556879652 | 0.0461020594018806 |  |
| 81539 | 'SLC38A1' | mRNA | -0.474275483 | 1.68116348543516E-5 |  |
| 81606 | 'LBH' | mRNA | -0.564564704 | 4.52230664468144E-7 |  |
| 81788 | 'NUAK2' | mRNA | -0.333992057 | 0.0413050577978857 |  |
| 8202 | 'NCOA3' | mRNA | -0.332996621 | 0.0125284428430955 |  |
| 8204 | 'NRIP1' | mRNA | -0.558982773 | 1.51023986282E-4 |  |
| 8237 | 'USP11' | mRNA | 0.495054678086321 | 3.19270197729E-4 |  |
| 8239 | 'USP9X' | mRNA | -0.265448042 | 0.0288508425558066 |  |
| 824 | 'CAPN2' | mRNA | -0.270769154 | 0.0372206014505225 |  |
| 8241 | 'RBM10' | mRNA | 0.453752464222678 | 0.0035687095549555 |  |
| 8273 | 'SLC10A3' | mRNA | 0.388017726390728 | 0.0277587226249784 |  |
| 829 | 'CAPZA1' | mRNA | -0.374238319 | 0.0064428213611239 |  |
| 8295 | 'TRRAP' | mRNA | 0.308609530025427 | 0.0266974560982377 |  |
| 831 | 'CAST' | mRNA | -0.822497143 | 5.08321334793372E-23 |  |
| 8313 | 'AXIN2' | mRNA | 0.378541563624065 | 0.0396321418869831 |  |
| 8322 | 'FZD4' | mRNA | -0.609567415 | 4.93660456483E-4 |  |
| 83448 | 'PUS7L' | mRNA | -0.580925736 | 0.0347820747715916 |  |
| 83604 | 'TMEM47' | mRNA | -0.423155298 | 0.0132259464518943 |  |
| 83660 | 'TLN2' | mRNA | 0.475557696858681 | 0.0398362914255003 |  |
| 83667 | 'SESN2' | mRNA | 0.358352859215234 | 0.0398870495270968 |  |
| 83696 | 'TRAPPC9' | mRNA | 0.399106216823942 | 0.0206961000944577 |  |
| 83874 | 'TBC1D10A' | mRNA | 0.492734252744077 | 0.0466336840595136 |  |
| 83939 | 'EIF2A' | mRNA | -0.477543719 | 0.002806111240836 |  |
| 83985 | 'SPNS1' | mRNA | 0.418472429373884 | 0.0074839717770876 |  |
| 83986 | 'FAM234A' | mRNA | 0.346495763773182 | 0.011545761712248 |  |
| 8408 | 'ULK1' | mRNA | 0.553370373319228 | 5.24425183501431E-5 |  |
| 8411 | 'EEA1' | mRNA | -0.770675047 | 0.002300689227099 |  |
| 84232 | 'MAF1' | mRNA | 0.324332170696638 | 0.025109013009175 |  |
| 84256 | 'FLYWCH1' | mRNA | 0.340942643136589 | 0.0261882944309094 |  |
| 8427 | 'ZNF282' | mRNA | 0.376897920657573 | 0.0338063453582558 |  |
| 84364 | 'ARFGAP2' | mRNA | 0.355742812123045 | 0.0345592637472811 |  |
| 84376 | 'HOOK3' | mRNA | -0.547614371 | 0.002225298787856 |  |
| 84464 | 'SLX4' | mRNA | 0.453423226973843 | 0.0374569512920364 |  |
| 84513 | 'PLPP5' | mRNA | -0.423471442 | 0.0395748023199041 |  |
| 84515 | 'MCM8' | mRNA | -0.598985657 | 0.0095875528815698 |  |
| 84629 | 'TNRC18' | mRNA | 0.33430574984983 | 0.0087644487982718 |  |
| 84726 | 'PRRC2B' | mRNA | 0.34408762785434 | 0.0045262941949938 |  |
| 8476 | 'CDC42BPA' | mRNA | -0.392879849 | 0.013256889170974 |  |
| 84858 | 'ZNF503' | mRNA | 0.4094988864598 | 0.0041807903171007 |  |
| 84913 | 'ATOH8' | mRNA | 0.910238604198887 | 9.118122637659651E-9 |  |
| 84926 | 'SPRYD3' | mRNA | 0.379601323730819 | 0.020752445405732 |  |
| 84951 | 'TNS4' | mRNA | -0.332758944 | 0.0116463662488314 |  |
| 84962 | 'AJUBA' | mRNA | 0.389262678074193 | 4.784782474897E-4 |  |
| 85004 | 'RERG' | mRNA | -0.731994966 | 0.0042671592455899 |  |
| 8503 | 'PIK3R3' | mRNA | 0.504696716265613 | 0.0338063453582558 |  |
| 8506 | 'CNTNAP1' | mRNA | 0.622501048958733 | 0.0409096650364154 |  |
| 8509 | 'NDST2' | mRNA | 0.514238894380941 | 0.0087644487982718 |  |
| 8535 | 'CBX4' | mRNA | 0.364601946902875 | 0.0140945810867352 |  |
| 85359 | 'DGCR6L' | mRNA | 0.412542503365983 | 0.0261317020690655 |  |
| 8537 | 'BCAS1' | mRNA | -0.579121718 | 7.60742607781385E-5 |  |
| 85415 | 'RHPN2' | mRNA | -0.29130269 | 0.0234798548129123 |  |
| 8543 | 'LMO4' | mRNA | -0.597427404 | 0.0016053928356211 |  |
| 85441 | 'HELZ2' | mRNA | 0.79626146648124 | 7.95583903810002E-9 |  |
| 857 | 'CAV1' | mRNA | -0.951878956 | 1.24741785967627E-23 |  |
| 8573 | 'CASK' | mRNA | -0.344149681 | 0.0197529964459831 |  |
| 8574 | 'AKR7A2' | mRNA | 0.41265654056397 | 0.0197529964459831 |  |
| 858 | 'CAV2' | mRNA | -0.311970494 | 0.0295592495821745 |  |
| 8614 | 'STC2' | mRNA | 1.27573627447028 | 4.17421589721915E-23 |  |
| 8615 | 'USO1' | mRNA | -0.316758926 | 0.0379949865187509 |  |
| 8667 | 'EIF3H' | mRNA | -0.30898516 | 0.0280539708806987 |  |
| 8671 | 'SLC4A4' | mRNA | -0.761284313 | 1.282992236591E-4 |  |
| 8722 | 'CTSF' | mRNA | 0.387219192591725 | 0.040581142451923 |  |
| 8729 | 'GBF1' | mRNA | 0.353641445453035 | 0.0066440636385224 |  |
| 8796 | 'SCEL' | mRNA | -1.390208458 | 1.19239944174464E-7 |  |
| 8821 | 'INPP4B' | mRNA | -0.735670788 | 3.170398434141E-4 |  |
| 8828 | 'NRP2' | mRNA | -0.378196461 | 0.0019966169352981 |  |
| 8857 | 'FCGBP' | mRNA | -0.713405555 | 6.78977865998353E-5 |  |
| 8870 | 'IER3' | mRNA | 0.29840717477928 | 0.0329386393469204 |  |
| 8878 | 'SQSTM1' | mRNA | 0.310053505276975 | 0.0046175340181869 |  |
| 8887 | 'TAX1BP1' | mRNA | -0.3342572 | 0.0181280036535081 |  |
| 8888 | 'MCM3AP' | mRNA | 0.284737401722649 | 0.0412407162785899 |  |
| 8899 | 'PRPF4B' | mRNA | -0.551355022 | 0.0021445152970085 |  |
| 8925 | 'HERC1' | mRNA | 0.349639360761909 | 0.0114436185393358 |  |
| 8930 | 'MBD4' | mRNA | -0.494035981 | 0.0454390610425998 |  |
| 8971 | 'H1-10' | mRNA | 0.397614662314689 | 0.038978438070652 |  |
| 8972 | 'MGAM' | mRNA | -1.129327589 | 1.25453373083039E-7 |  |
| 8974 | 'P4HA2' | mRNA | 0.329143618537589 | 0.0240876018300449 |  |
| 90007 | 'MIDN' | mRNA | 0.350657604270726 | 0.0279399992662577 |  |
| 90113 | 'VWA5B2' | mRNA | 0.972164534715824 | 0.0113270292571697 |  |
| 90313 | 'TP53I13' | mRNA | 0.499225869481983 | 0.0091079418538916 |  |
| 90324 | 'CCDC97' | mRNA | 0.444787198189083 | 0.0032476357965732 |  |
| 90427 | 'BMF' | mRNA | 1.52255210915917 | 0.0186547538167592 |  |
| 9056 | 'SLC7A7' | mRNA | -0.380989853 | 0.0140945810867352 |  |
| 9060 | 'PAPSS2' | mRNA | -0.591336475 | 2.27692469278027E-5 |  |
| 9075 | 'CLDN2' | mRNA | -0.595376977 | 1.88773437327242E-8 |  |
| 90933 | 'TRIM41' | mRNA | 0.427354342745326 | 0.0097771579468838 |  |
| 9126 | 'SMC3' | mRNA | -0.670353382 | 0.0028945910809603 |  |
| 91461 | 'PKDCC' | mRNA | -0.665980436 | 8.14218407694078E-5 |  |
| 9147 | 'NEMF' | mRNA | -0.66252339 | 0.0014838473035597 |  |
| 91523 | 'PCED1B' | mRNA | -0.742785149 | 6.3647054540331E-8 |  |
| 91683 | 'SYT12' | mRNA | -1.095780055 | 0.0448231973006968 |  |
| 91768 | 'CABLES1' | mRNA | 0.402399378755845 | 0.0034090428144693 |  |
| 9182 | 'RASSF9' | mRNA | -0.891808585 | 0.0388206602980555 |  |
| 9194 | 'SLC16A7' | mRNA | -0.960608665 | 8.03667180938403E-5 |  |
| 9202 | 'ZMYM4' | mRNA | -0.386310097 | 0.0223907690693749 |  |
| 9208 | 'LRRFIP1' | mRNA | -0.465423914 | 5.300499130615E-4 |  |
| 9231 | 'DLG5' | mRNA | 0.300841822116494 | 0.0127725854446831 |  |
| 9236 | 'CCPG1' | mRNA | -0.507397822 | 4.759153576731E-4 |  |
| 9246 | 'UBE2L6' | mRNA | 0.37844725661231 | 0.0104870676891214 |  |
| 9249 | 'DHRS3' | mRNA | 0.326956620618738 | 0.0115381521949064 |  |
| 9253 | 'NUMBL' | mRNA | 0.422210412676074 | 0.0270817435829103 |  |
| 9289 | 'ADGRG1' | mRNA | -0.282962428 | 0.0462618277622057 |  |
| 9317 | 'PTER' | mRNA | -0.499659299 | 0.0487385832649764 |  |
| 9321 | 'TRIP11' | mRNA | -0.668812561 | 0.004880869563011 |  |
| 9324 | 'HMGN3' | mRNA | -0.428564231 | 0.0373097970760172 |  |
| 9344 | 'TAOK2' | mRNA | 0.414957234837435 | 0.0027831853998473 |  |
| 9360 | 'PPIG' | mRNA | -0.762782025 | 0.0020150960472533 |  |
| 93663 | 'ARHGAP18' | mRNA | -0.525434009 | 0.0021519638908948 |  |
| 94025 | 'MUC16' | mRNA | -0.764480912 | 5.99474348687685E-8 |  |
| 9411 | 'ARHGAP29' | mRNA | -0.454657654 | 0.0489640764419677 |  |
| 94134 | 'ARHGAP12' | mRNA | -0.44560944 | 0.0029642904957465 |  |
| 9415 | 'FADS2' | mRNA | -0.400147458 | 2.082072664568E-4 |  |
| 94234 | 'FOXQ1' | mRNA | -0.507436056 | 0.009366354184135 |  |
| 94241 | 'TP53INP1' | mRNA | 0.540989148349515 | 1.21409251055163E-5 |  |
| 9469 | 'CHST3' | mRNA | -0.290617893 | 0.0127661472743625 |  |
| 9475 | 'ROCK2' | mRNA | -0.376759986 | 0.0207409645726323 |  |
| 9479 | 'MAPK8IP1' | mRNA | 0.636331395083271 | 9.755300412002E-4 |  |
| 949 | 'SCARB1' | mRNA | 0.266976009863494 | 0.0288508425558066 |  |
| 9513 | 'FXR2' | mRNA | 0.357368667080472 | 0.0074111354030506 |  |
| 9540 | 'TP53I3' | mRNA | 0.272149567233904 | 0.040587694465769 |  |
| 9563 | 'H6PD' | mRNA | 0.300943412911542 | 0.0466174170911604 |  |
| 9585 | 'KIF20B' | mRNA | -1.107557045 | 0.0079453634909844 |  |
| 9592 | 'IER2' | mRNA | 0.49553491245523 | 0.0018659957146609 |  |
| 960 | 'CD44' | mRNA | -0.435115285 | 3.61628272495255E-6 |  |
| 9615 | 'GDA' | mRNA | -0.727499469 | 2.27692469278027E-5 |  |
| 9619 | 'ABCG1' | mRNA | 0.747325751102807 | 0.0229766115282648 |  |
| 9648 | 'GCC2' | mRNA | -0.838283566 | 8.621646270839E-4 |  |
| 9655 | 'SOCS5' | mRNA | -0.634463997 | 0.0029891005162008 |  |
| 9669 | 'EIF5B' | mRNA | -0.52754731 | 1.403287934479E-4 |  |
| 9682 | 'KDM4A' | mRNA | 0.340106256455938 | 0.0208824922106598 |  |
| 9684 | 'LRRC14' | mRNA | 0.463276616881369 | 0.005719919345909 |  |
| 9689 | 'BZW1' | mRNA | -0.362878296 | 0.0112913917892571 |  |
| 970 | 'CD70' | mRNA | -0.524158294 | 0.0096738735460767 |  |
| 9726 | 'ZNF646' | mRNA | 0.41835424847085 | 0.0333219772671347 |  |
| 9727 | 'RAB11FIP3' | mRNA | 0.457185741245825 | 0.0011462144041949 |  |
| 9736 | 'USP34' | mRNA | -0.446424446 | 7.372678898076E-4 |  |
| 9748 | 'SLK' | mRNA | -0.563173513 | 8.84475628808031E-5 |  |
| 9751 | 'SNPH' | mRNA | 0.502077882699381 | 0.0037919760676909 |  |
| 9757 | 'KMT2B' | mRNA | 0.360453473478411 | 0.0215501798205306 |  |
| 9774 | 'BCLAF1' | mRNA | -0.591486816 | 5.77511459411413E-5 |  |
| 9794 | 'MAML1' | mRNA | 0.383412467141257 | 0.0069073424455328 |  |
| 9804 | 'TOMM20' | mRNA | -0.331799683 | 0.0211962067744015 |  |
| 9807 | 'IP6K1' | mRNA | 0.350260042546916 | 0.0444116190000999 |  |
| 9810 | 'RNF40' | mRNA | 0.390919806659716 | 0.0066903596951009 |  |
| 9821 | 'RB1CC1' | mRNA | -0.511022592 | 0.0281127661322183 |  |
| 9826 | 'ARHGEF11' | mRNA | 0.321679705603778 | 0.022736591209885 |  |
| 9848 | 'MFAP3L' | mRNA | -0.585654111 | 0.0237003800253977 |  |
| 9857 | 'CEP350' | mRNA | -0.868643494 | 7.14504417536459E-6 |  |
| 9859 | 'CEP170' | mRNA | -0.454193969 | 0.0170621907410914 |  |
| 9875 | 'URB1' | mRNA | 0.31152342729711 | 0.043800610007235 |  |
| 9877 | 'ZC3H11A' | mRNA | -0.418824444 | 0.0059833280044072 |  |
| 9879 | 'DDX46' | mRNA | -0.424179706 | 0.001235738799526 |  |
| 9886 | 'RHOBTB1' | mRNA | -0.472588982 | 0.0012077864684017 |  |
| 9898 | 'UBAP2L' | mRNA | 0.278736355618046 | 0.0448231973006968 |  |
| 9915 | 'ARNT2' | mRNA | 0.520523601664505 | 0.0046175340181869 |  |
| 9933 | 'PUM3' | mRNA | -0.58899317 | 0.0043876836980237 |  |
| 994 | 'CDC25B' | mRNA | 0.485801674157165 | 3.24148464452871E-5 |  |
| 9958 | 'USP15' | mRNA | -0.59362564 | 0.0032476357965732 |  |
| 9966 | 'TNFSF15' | mRNA | -0.829422235 | 1.654363809212E-5 |  |
| 9968 | 'MED12' | mRNA | 0.488228782099317 | 0.0095875528815698 |  |
| 9969 | 'MED13' | mRNA | -0.424833292 | 0.0047971238302106 |  |
| 9971 | 'NR1H4' | mRNA | -0.853370623 | 0.0015485794176422 |  |
| 9980 | 'DOP1B' | mRNA | 0.433271520280972 | 0.0413050577978857 |  |
| 9982 | 'FGFBP1' | mRNA | -0.606554311 | 0.0368939236240096 |  |
| 999 | 'CDH1' | mRNA | -0.425264809 | 1.087965944978E-4 |  |
| 9993 | 'DGCR2' | mRNA | 0.327889252155055 | 0.0303406383242738 |  |
| 9994 | 'CASP8AP2' | mRNA | -0.975599185 | 0.0489261006592623 |  |

Differentially expressed genes in A549

| Gene ID | Gene Symbol | Type | log2 (PD_3 / PD_0) | FDR (PD_3 / PD_0) |
| --- | --- | --- | --- | --- |
| 100128553 | 'CTAGE4' | mRNA | 1.116363142 | 3.13E-13 |
| 100288142 | 'NBPF20' | mRNA | 2.08146746 | 6.21E-44 |
| 100288332 | 'NPIPA5' | mRNA | 1.092964701 | 3.00E-66 |
| 100288695 | 'LIMS4' | mRNA | 11.70994538 | 1.54E-10 |
| 100507747 | 'C13orf46' | mRNA | 3.807354922 | 8.51E-04 |
| 100526737 | 'RBM14-RBM4' | mRNA | -1.437130225 | 8.07E-04 |
| 100526740 | 'ATP5MF-PTCD1' | mRNA | -1.78433229 | 1.29E-27 |
| 100526832 | 'PHOSPHO2-KLHL23' | mRNA | -11.85642553 | 5.03E-10 |
| 100528017 | 'SAA2-SAA4' | mRNA | -3.103950964 | 2.52E-13 |
| 100528064 | 'NEDD8-MDP1' | mRNA | 12.1792871 | 1.25E-14 |
| 100529063 | 'BCL2L2-PABPN1' | mRNA | 1.092320299 | 3.42E-04 |
| 100529241 | 'HSPE1-MOB4' | mRNA | 1.563478034 | 5.85E-25 |
| 100532726 | 'NDUFC2-KCTD14' | mRNA | 11.9465408 | 1.98E-12 |
| 10058 | 'ABCB6' | mRNA | -1.017901933 | 2.54E-29 |
| 10076 | 'PTPRU' | mRNA | -1.085807154 | 1.26E-23 |
| 100996717 | 'NOTCH2NLC' | mRNA | 1.051811435 | 1.54E-05 |
| 10100 | 'TSPAN2' | mRNA | -1.087760968 | 1.32E-15 |
| 101060226 | 'NBPF19' | mRNA | 1.102694758 | 3.43E-33 |
| 101060389 | 'TBC1D3D' | mRNA | 1.100964445 | 3.21E-05 |
| 1014 | 'CDH16' | mRNA | 2.04915003 | 9.34E-16 |
| 10158 | 'PDZK1IP1' | mRNA | -2.118394701 | 3.93E-16 |
| 10232 | 'MSLN' | mRNA | -1.028951025 | 6.47E-31 |
| 102723547 | 'CSAG2' | mRNA | -12.50828999 | 1.20E-15 |
| 102724428 | 'SIK1B' | mRNA | -1.301071326 | 2.39E-10 |
| 102724594 | 'U2AF1L5' | mRNA | -1.236419252 | 3.78E-22 |
| 10384 | 'BTN3A3' | mRNA | -1.508629376 | 2.08E-05 |
| 10398 | 'MYL9' | mRNA | -1.366582257 | 4.79E-10 |
| 10451 | 'VAV3' | mRNA | -1.497499659 | 5.22E-06 |
| 10501 | 'SEMA6B' | mRNA | -1.022815201 | 3.30E-14 |
| 10529 | 'NEBL' | mRNA | -1.076566612 | 2.16E-06 |
| 105375355 | 'UPK3B' | mRNA | -1.122751615 | 5.22E-10 |
| 10550 | 'ARL6IP5' | mRNA | -1.102343844 | 1.61E-26 |
| 10551 | 'AGR2' | mRNA | -2.974733241 | 1.61E-41 |
| 10763 | 'NES' | mRNA | -1.066847598 | 3.62E-23 |
| 107984590 | 'LOC107984590' | mRNA | -1.052088511 | 7.99E-05 |
| 107987464 | 'LOC107987464' | mRNA | 4.487840034 | 1.38E-05 |
| 107987477 | 'LOC107987477' | mRNA | -10.35424938 | 9.51E-04 |
| 10873 | 'ME3' | mRNA | -1.047449755 | 3.78E-29 |
| 10882 | 'C1QL1' | mRNA | -1.161463423 | 9.33E-04 |
| 11098 | 'PRSS23' | mRNA | -1.343660975 | 1.52E-152 |
| 11099 | 'PTPN21' | mRNA | 1.088866024 | 2.55E-07 |
| 11119 | 'BTN3A1' | mRNA | -1.117386702 | 2.64E-12 |
| 11151 | 'CORO1A' | mRNA | -1.468310518 | 6.81E-07 |
| 11174 | 'ADAMTS6' | mRNA | 2.222392421 | 6.56E-22 |
| 11182 | 'SLC2A6' | mRNA | -1.035855658 | 3.20E-15 |
| 11186 | 'RASSF1' | mRNA | 1.166134839 | 1.51E-54 |
| 11221 | 'DUSP10' | mRNA | 1.06826091 | 5.72E-16 |
| 112399 | 'EGLN3' | mRNA | -2.527737632 | 3.21E-21 |
| 1124 | 'CHN2' | mRNA | -1.061927749 | 2.86E-04 |
| 112476 | 'PRRT2' | mRNA | -1.960388144 | 1.46E-04 |
| 11259 | 'FILIP1L' | mRNA | 1.310903626 | 4.18E-52 |
| 11272 | 'PRR4' | mRNA | 1.129567264 | 1.74E-04 |
| 11277 | 'TREX1' | mRNA | -1.965958691 | 6.07E-07 |
| 11346 | 'SYNPO' | mRNA | -1.616588314 | 1.89E-07 |
| 113730 | 'KLHDC7B' | mRNA | -1.810074423 | 2.51E-14 |
| 114336 | 'CGB2' | mRNA | 2.214570686 | 1.46E-05 |
| 114780 | 'PKD1L2' | mRNA | -1.751320887 | 7.41E-08 |
| 114990 | 'VASN' | mRNA | -1.043068722 | 2.40E-08 |
| 115072896 | 'SLCO1B3-SLCO1B7' | mRNA | 1.704964641 | 3.32E-13 |
| 115273 | 'RAB42' | mRNA | -1.787549862 | 3.48E-09 |
| 116039 | 'OSR2' | mRNA | -1.327261539 | 1.22E-12 |
| 116372 | 'LYPD1' | mRNA | -1.133030396 | 2.80E-08 |
| 1183 | 'CLCN4' | mRNA | -1.415246601 | 4.80E-10 |
| 118738 | 'ZNF488' | mRNA | -2.009984089 | 1.72E-38 |
| 118788 | 'PIK3AP1' | mRNA | -1.592462603 | 6.61E-06 |
| 118932 | 'ANKRD22' | mRNA | -1.232325199 | 3.72E-23 |
| 120071 | 'LARGE2' | mRNA | -1.408805546 | 2.70E-08 |
| 120939 | 'TMEM52B' | mRNA | -1.441500613 | 1.11E-16 |
| 121129 | 'OR2AP1' | mRNA | 2.251570267 | 6.93E-04 |
| 121506 | 'ERP27' | mRNA | -2.013674937 | 1.25E-37 |
| 121551 | 'BTBD11' | mRNA | 1.021270687 | 2.59E-11 |
| 122945 | 'NOXRED1' | mRNA | 1.353785939 | 1.70E-06 |
| 124790 | 'HEXIM2' | mRNA | -1.040872281 | 1.16E-10 |
| 125965 | 'COX6B2' | mRNA | 2.430872703 | 2.74E-34 |
| 1265 | 'CNN2' | mRNA | -1.055285481 | 8.90E-168 |
| 126669 | 'SHE' | mRNA | 1.725302068 | 6.24E-06 |
| 1284 | 'COL4A2' | mRNA | -1.058270285 | 1.51E-56 |
| 128434 | 'VSTM2L' | mRNA | -1.101879614 | 5.97E-09 |
| 1289 | 'COL5A1' | mRNA | -2.171336641 | 1.22E-74 |
| 1291 | 'COL6A1' | mRNA | -1.021618576 | 3.64E-31 |
| 1295 | 'COL8A1' | mRNA | -1.192627731 | 3.42E-05 |
| 13 | 'AADAC' | mRNA | 1.326810316 | 1.63E-11 |
| 1303 | 'COL12A1' | mRNA | -1.609853676 | 1.06E-21 |
| 130576 | 'LYPD6B' | mRNA | -2.062840874 | 3.40E-28 |
| 132014 | 'IL17RE' | mRNA | -1.316587843 | 7.63E-18 |
| 1326 | 'MAP3K8' | mRNA | -1.04996799 | 1.88E-08 |
| 132864 | 'CPEB2' | mRNA | 1.025535092 | 8.93E-04 |
| 134 | 'ADORA1' | mRNA | -1.52144605 | 3.24E-29 |
| 135932 | 'TMEM139' | mRNA | -1.390269594 | 4.19E-30 |
| 138162 | 'C9orf116' | mRNA | -1.084043452 | 4.68E-10 |
| 1397 | 'CRIP2' | mRNA | -1.417017863 | 1.00E-12 |
| 140893 | 'RBBP8NL' | mRNA | -1.511577447 | 3.10E-12 |
| 144321 | 'GLIPR1L2' | mRNA | 1.04299395 | 1.63E-04 |
| 144501 | 'KRT80' | mRNA | -1.042383549 | 7.66E-92 |
| 147 | 'ADRA1B' | mRNA | -1.704334328 | 1.52E-24 |
| 1490 | 'CCN2' | mRNA | -1.182203331 | 5.21E-05 |
| 149483 | 'CCDC17' | mRNA | 2.021270687 | 2.99E-09 |
| 150696 | 'PROM2' | mRNA | -1.017633613 | 7.40E-69 |
| 1509 | 'CTSD' | mRNA | -1.024388676 | 0 |
| 151393 | 'RMDN2' | mRNA | 1.516557063 | 4.56E-251 |
| 152110 | 'NEK10' | mRNA | 1.655405965 | 1.92E-08 |
| 152559 | 'PAQR3' | mRNA | 1.025415984 | 3.78E-15 |
| 154091 | 'SLC2A12' | mRNA | -1.387444748 | 7.69E-04 |
| 1543 | 'CYP1A1' | mRNA | 5.499845887 | 2.01E-51 |
| 1545 | 'CYP1B1' | mRNA | 1.586091533 | 7.28E-68 |
| 154796 | 'AMOT' | mRNA | -2.121306296 | 5.86E-24 |
| 155054 | 'ZNF425' | mRNA | 1.043437798 | 1.07E-07 |
| 1565 | 'CYP2D6' | mRNA | 1.957343653 | 7.49E-09 |
| 1604 | 'CD55' | mRNA | 1.245949223 | 2.96E-53 |
| 160419 | 'C12orf50' | mRNA | 1.042529771 | 7.51E-21 |
| 160622 | 'TAMALIN' | mRNA | 1.678071905 | 5.76E-04 |
| 163782 | 'KANK4' | mRNA | -1.73892668 | 2.70E-06 |
| 168544 | 'ZNF467' | mRNA | -1.282933963 | 7.96E-07 |
| 169611 | 'OLFML2A' | mRNA | -1.389302098 | 5.29E-17 |
| 171177 | 'RHOV' | mRNA | -1.023083613 | 1.55E-07 |
| 1717 | 'DHCR7' | mRNA | -1.020611409 | 7.99E-131 |
| 18 | 'ABAT' | mRNA | -1.042580866 | 9.54E-13 |
| 1808 | 'DPYSL2' | mRNA | -1.014207646 | 3.53E-21 |
| 1815 | 'DRD4' | mRNA | 2.753818443 | 4.15E-05 |
| 1843 | 'DUSP1' | mRNA | 2.306498054 | 1.07E-67 |
| 1850 | 'DUSP8' | mRNA | 1.119006279 | 1.38E-12 |
| 19 | 'ABCA1' | mRNA | 1.706953025 | 6.52E-12 |
| 1942 | 'EFNA1' | mRNA | -1.122346245 | 5.40E-39 |
| 196996 | 'GRAMD2A' | mRNA | -1.487103566 | 8.83E-46 |
| 1999 | 'ELF3' | mRNA | -1.024475231 | 1.33E-48 |
| 203102 | 'ADAM32' | mRNA | 2.60865923 | 5.97E-20 |
| 2049 | 'EPHB3' | mRNA | -1.096177502 | 3.11E-11 |
| 2051 | 'EPHB6' | mRNA | -1.139274789 | 3.02E-22 |
| 2069 | 'EREG' | mRNA | 1.600962216 | 8.86E-14 |
| 2138 | 'EYA1' | mRNA | -2.03562391 | 2.13E-04 |
| 2171 | 'FABP5' | mRNA | 10.34540525 | 2.24E-04 |
| 2192 | 'FBLN1' | mRNA | -1.006744328 | 2.09E-113 |
| 2194 | 'FASN' | mRNA | -1.02483358 | 1.41E-290 |
| 2202 | 'EFEMP1' | mRNA | -2.055205502 | 2.24E-53 |
| 221421 | 'RSPH9' | mRNA | 1.443448902 | 8.04E-04 |
| 222 | 'ALDH3B2' | mRNA | -1.346098945 | 1.97E-05 |
| 222171 | 'PRR15' | mRNA | -1.206424161 | 1.75E-20 |
| 2264 | 'FGFR4' | mRNA | -1.488352983 | 3.45E-13 |
| 22979 | 'EFR3B' | mRNA | -1.0424218 | 7.15E-05 |
| 230 | 'ALDOC' | mRNA | -1.016676425 | 2.17E-08 |
| 23220 | 'DTX4' | mRNA | -1.347923303 | 1.83E-23 |
| 23240 | 'TMEM131L' | mRNA | -1.257717554 | 7.67E-06 |
| 23349 | 'PHF24' | mRNA | 10.10328781 | 9.01E-04 |
| 2335 | 'FN1' | mRNA | -1.50768111 | 0 |
| 23362 | 'PSD3' | mRNA | 1.029440803 | 2.85E-11 |
| 23376 | 'UFL1' | mRNA | 1.185692317 | 1.10E-13 |
| 2342 | 'FNTB' | mRNA | -1.368559854 | 1.46E-05 |
| 23551 | 'RASD2' | mRNA | -2.029747343 | 4.46E-05 |
| 23569 | 'PADI4' | mRNA | -1.854648614 | 6.93E-04 |
| 23596 | 'OPN3' | mRNA | -1.045149386 | 1.16E-43 |
| 23630 | 'KCNE5' | mRNA | 1.353636955 | 2.88E-04 |
| 23645 | 'PPP1R15A' | mRNA | 1.572288281 | 0 |
| 23657 | 'SLC7A11' | mRNA | 1.132908254 | 7.68E-85 |
| 23753 | 'SDF2L1' | mRNA | -1.178579467 | 8.29E-23 |
| 24146 | 'CLDN15' | mRNA | 1.183711953 | 3.10E-12 |
| 245711 | 'SPDYA' | mRNA | 1.426774823 | 1.12E-07 |
| 2487 | 'FRZB' | mRNA | 2.435386145 | 8.69E-19 |
| 250 | 'ALPP' | mRNA | -1.245085843 | 9.51E-100 |
| 255324 | 'EPGN' | mRNA | 1.086143516 | 2.09E-08 |
| 255738 | 'PCSK9' | mRNA | -2.26589406 | 4.43E-11 |
| 25805 | 'BAMBI' | mRNA | -1.127605537 | 3.45E-09 |
| 25806 | 'VAX2' | mRNA | 1.757316931 | 6.82E-04 |
| 25832 | 'NBPF14' | mRNA | 1.047835475 | 3.10E-72 |
| 25837 | 'RAB26' | mRNA | -1.388431259 | 5.33E-29 |
| 25854 | 'FAM149A' | mRNA | 10.12670447 | 9.01E-04 |
| 25976 | 'TIPARP' | mRNA | 1.472269576 | 1.34E-97 |
| 25981 | 'DNAH1' | mRNA | 1.592776282 | 1.67E-13 |
| 26287 | 'ANKRD2' | mRNA | -2.210396114 | 2.59E-40 |
| 27 | 'ABL2' | mRNA | 1.13957473 | 1.29E-62 |
| 2702 | 'GJA5' | mRNA | -3 | 3.61E-04 |
| 2706 | 'GJB2' | mRNA | -1.178337241 | 7.20E-04 |
| 27063 | 'ANKRD1' | mRNA | -1.842760258 | 2.85E-13 |
| 27113 | 'BBC3' | mRNA | -1.000584995 | 9.06E-05 |
| 27134 | 'TJP3' | mRNA | -1.706008782 | 4.13E-40 |
| 27151 | 'CPAMD8' | mRNA | -1.700955396 | 4.82E-12 |
| 27198 | 'HCAR1' | mRNA | -1.342855022 | 9.40E-04 |
| 27345 | 'KCNMB4' | mRNA | -1.332601722 | 5.03E-07 |
| 2744 | 'GLS' | mRNA | 1.268920133 | 6.59E-63 |
| 2810 | 'SFN' | mRNA | 1.628331703 | 0 |
| 2822 | 'GPLD1' | mRNA | -1.800398263 | 3.16E-04 |
| 282617 | 'IFNL3' | mRNA | -1.918838449 | 1.33E-06 |
| 283106 | 'CSNK2A3' | mRNA | -12.53113774 | 6.45E-16 |
| 283234 | 'CCDC88B' | mRNA | -1.330898248 | 3.64E-06 |
| 284611 | 'FAM102B' | mRNA | -1.047839315 | 4.37E-07 |
| 2852 | 'GPER1' | mRNA | -1.24961389 | 4.96E-05 |
| 285489 | 'DOK7' | mRNA | -1.40204274 | 3.85E-13 |
| 285672 | 'SREK1IP1' | mRNA | 1.137329385 | 5.71E-34 |
| 285704 | 'RGMB' | mRNA | 1.29243314 | 4.66E-60 |
| 285955 | 'SPDYE1' | mRNA | 1.184152053 | 3.77E-06 |
| 286006 | 'LSMEM1' | mRNA | 1.171252315 | 2.04E-04 |
| 286204 | 'CRB2' | mRNA | 3.760172985 | 4.48E-04 |
| 286336 | 'FAM78A' | mRNA | -1.151164073 | 3.09E-07 |
| 286343 | 'LURAP1L' | mRNA | 1.574159189 | 4.25E-12 |
| 28951 | 'TRIB2' | mRNA | -1.70056302 | 2.15E-16 |
| 28996 | 'HIPK2' | mRNA | 1.158903595 | 1.75E-20 |
| 290 | 'ANPEP' | mRNA | -1.157541277 | 9.95E-07 |
| 2919 | 'CXCL1' | mRNA | -1.209349146 | 2.32E-05 |
| 2953 | 'GSTT2' | mRNA | 10.17617315 | 9.00E-04 |
| 2979 | 'GUCA1B' | mRNA | 1.065834644 | 8.78E-04 |
| 29893 | 'PSMC3IP' | mRNA | 1.353265855 | 5.04E-14 |
| 29948 | 'OSGIN1' | mRNA | 1.466623806 | 7.64E-178 |
| 29993 | 'PACSIN1' | mRNA | -1.095277241 | 1.89E-09 |
| 3038 | 'HAS3' | mRNA | 1.392339232 | 5.51E-52 |
| 3059 | 'HCLS1' | mRNA | 2.678071905 | 4.49E-06 |
| 309 | 'ANXA6' | mRNA | -1.418312631 | 1.22E-51 |
| 3188 | 'HNRNPH2' | mRNA | 11.85681455 | 8.45E-12 |
| 3242 | 'HPD' | mRNA | 1.137503524 | 4.27E-04 |
| 3283 | 'HSD3B1' | mRNA | -1.475733431 | 1.63E-05 |
| 330 | 'BIRC3' | mRNA | -1.119146254 | 9.98E-07 |
| 3309 | 'HSPA5' | mRNA | -1.020012585 | 2.38E-241 |
| 3371 | 'TNC' | mRNA | -1.176995484 | 6.56E-62 |
| 3383 | 'ICAM1' | mRNA | -1.022487799 | 4.01E-64 |
| 338376 | 'IFNE' | mRNA | 2.898120386 | 1.69E-15 |
| 338442 | 'HCAR2' | mRNA | -2.024210554 | 3.04E-05 |
| 339122 | 'RAB43' | mRNA | -2.015509708 | 1.73E-50 |
| 339766 | 'MROH2A' | mRNA | 3.64385619 | 4.12E-06 |
| 3399 | 'ID3' | mRNA | -1.08435891 | 9.27E-48 |
| 3417 | 'IDH1' | mRNA | -1.074118833 | 2.43E-27 |
| 342897 | 'NCCRP1' | mRNA | -1.258165366 | 2.69E-04 |
| 342979 | 'PALM3' | mRNA | -1.501194143 | 8.96E-11 |
| 347148 | 'QRFP' | mRNA | 2.263034406 | 1.80E-07 |
| 3481 | 'IGF2' | mRNA | -1.168284868 | 6.67E-08 |
| 3485 | 'IGFBP2' | mRNA | -1.097550423 | 1.22E-22 |
| 3486 | 'IGFBP3' | mRNA | -2.46712601 | 6.75E-66 |
| 3570 | 'IL6R' | mRNA | 1.361113253 | 5.21E-15 |
| 3589 | 'IL11' | mRNA | 3.105732211 | 0 |
| 360 | 'AQP3' | mRNA | -1.504472583 | 1.87E-07 |
| 3623 | 'INHA' | mRNA | -1.370837695 | 3.23E-11 |
| 3627 | 'CXCL10' | mRNA | -1.847996907 | 6.52E-05 |
| 3689 | 'ITGB2' | mRNA | -1.35310975 | 1.52E-14 |
| 3694 | 'ITGB6' | mRNA | -1.349584438 | 6.26E-08 |
| 3725 | 'JUN' | mRNA | 1.210643069 | 1.54E-169 |
| 3732 | 'CD82' | mRNA | -1.678701798 | 7.41E-06 |
| 3743 | 'KCNA7' | mRNA | 2.475298869 | 4.15E-05 |
| 375593 | 'TRIM73' | mRNA | 10.78381676 | 1.38E-05 |
| 375790 | 'AGRN' | mRNA | -1.109441915 | 1.85E-161 |
| 3783 | 'KCNN4' | mRNA | -1.001651566 | 7.27E-36 |
| 388 | 'RHOB' | mRNA | 1.422787046 | 5.93E-59 |
| 3885 | 'KRT34' | mRNA | 1.634593268 | 9.08E-04 |
| 390595 | 'UBAP1L' | mRNA | 1.02324625 | 1.30E-08 |
| 3931 | 'LCAT' | mRNA | 1.012044404 | 6.24E-08 |
| 3955 | 'LFNG' | mRNA | -1.033423002 | 2.04E-04 |
| 397 | 'ARHGDIB' | mRNA | -1.237490766 | 1.59E-80 |
| 3981 | 'LIG4' | mRNA | 1.054447784 | 1.44E-06 |
| 400954 | 'EML6' | mRNA | 1.40546242 | 2.88E-04 |
| 4017 | 'LOXL2' | mRNA | -1.300917747 | 2.94E-51 |
| 401934 | 'RNF223' | mRNA | 1.387772266 | 6.48E-34 |
| 4047 | 'LSS' | mRNA | -1.204736784 | 1.64E-32 |
| 4050 | 'LTB' | mRNA | -2.157541277 | 2.58E-06 |
| 4059 | 'BCAM' | mRNA | -1.024803213 | 4.08E-51 |
| 408 | 'ARRB1' | mRNA | -1.062350513 | 7.76E-50 |
| 408050 | 'NOMO3' | mRNA | 1.049004933 | 1.26E-08 |
| 4091 | 'SMAD6' | mRNA | -2.007992791 | 2.27E-16 |
| 4099 | 'MAG' | mRNA | 2.074000581 | 6.73E-11 |
| 41 | 'ASIC1' | mRNA | -1.024494562 | 9.67E-09 |
| 4128 | 'MAOA' | mRNA | 1.16384219 | 5.34E-11 |
| 414061 | 'DNAJB3' | mRNA | 1.502500341 | 9.72E-04 |
| 414325 | 'DEFB103A' | mRNA | 3.931683057 | 1.62E-07 |
| 4157 | 'MC1R' | mRNA | 1.526688248 | 2.46E-22 |
| 4242 | 'MFNG' | mRNA | 1.584962501 | 3.21E-17 |
| 4256 | 'MGP' | mRNA | -2.307392002 | 1.89E-09 |
| 4318 | 'MMP9' | mRNA | -2.078002512 | 3.12E-04 |
| 4320 | 'MMP11' | mRNA | -1.584962501 | 1.42E-04 |
| 4352 | 'MPL' | mRNA | 1.628276239 | 5.76E-04 |
| 440 | 'ASNS' | mRNA | 1.093069778 | 1.63E-82 |
| 440567 | 'UQCRHL' | mRNA | -7.200762405 | 9.51E-04 |
| 445 | 'ASS1' | mRNA | -1.261756507 | 3.74E-238 |
| 4582 | 'MUC1' | mRNA | -1.477098467 | 4.83E-41 |
| 467 | 'ATF3' | mRNA | 2.399794766 | 1.85E-45 |
| 4688 | 'NCF2' | mRNA | 1.377836043 | 2.06E-307 |
| 474381 | 'H2AB2' | mRNA | 10.10328781 | 9.00E-04 |
| 4774 | 'NFIA' | mRNA | -1.045642666 | 7.54E-09 |
| 487 | 'ATP2A1' | mRNA | 1.244255041 | 5.18E-04 |
| 489 | 'ATP2A3' | mRNA | -1.185214872 | 1.03E-06 |
| 4891 | 'SLC11A2' | mRNA | -1.145925585 | 1.41E-19 |
| 493861 | 'EID3' | mRNA | 2.004501392 | 4.93E-40 |
| 497190 | 'CLEC18B' | mRNA | 1.450277318 | 2.08E-07 |
| 4973 | 'OLR1' | mRNA | -1.485599642 | 6.09E-68 |
| 49854 | 'ZBTB21' | mRNA | 1.1091552 | 1.84E-13 |
| 5029 | 'P2RY2' | mRNA | -1.044520799 | 1.52E-26 |
| 5031 | 'P2RY6' | mRNA | -1.657317149 | 3.61E-09 |
| 5042 | 'PABPC3' | mRNA | 1.559821548 | 4.22E-06 |
| 50617 | 'ATP6V0A4' | mRNA | -1.249631701 | 5.76E-06 |
| 50619 | 'DEF6' | mRNA | -1.23878686 | 2.20E-05 |
| 5080 | 'PAX6' | mRNA | 1.008570261 | 6.37E-04 |
| 5087 | 'PBX1' | mRNA | -1.291462814 | 1.22E-07 |
| 5099 | 'PCDH7' | mRNA | -1.6112124 | 4.80E-45 |
| 51090 | 'PLLP' | mRNA | -1.856264523 | 3.62E-41 |
| 51276 | 'ZNF571' | mRNA | 1.337034987 | 2.33E-04 |
| 5129 | 'CDK18' | mRNA | -1.130415694 | 6.55E-18 |
| 51332 | 'SPTBN5' | mRNA | 1.46712601 | 1.01E-04 |
| 5138 | 'PDE2A' | mRNA | 1.077076223 | 2.94E-10 |
| 51435 | 'SCARA3' | mRNA | -1.179565251 | 1.24E-08 |
| 51442 | 'VGLL1' | mRNA | -1.218411211 | 1.15E-95 |
| 51700 | 'CYB5R2' | mRNA | -1.73039294 | 8.34E-16 |
| 51704 | 'GPRC5B' | mRNA | -1.922081146 | 8.64E-81 |
| 5342 | 'PLGLB2' | mRNA | 1.505941433 | 9.72E-04 |
| 5345 | 'SERPINF2' | mRNA | -1.508837545 | 2.20E-10 |
| 5362 | 'PLXNA2' | mRNA | -1.061086323 | 5.31E-23 |
| 53630 | 'BCO1' | mRNA | -2.807354922 | 1.78E-04 |
| 5366 | 'PMAIP1' | mRNA | 1.057947349 | 1.34E-07 |
| 53833 | 'IL20RB' | mRNA | 1.057615271 | 1.74E-08 |
| 54453 | 'RIN2' | mRNA | -1.041820176 | 2.36E-13 |
| 54504 | 'CPVL' | mRNA | -1.108165405 | 1.60E-34 |
| 54550 | 'NECAB2' | mRNA | 3.285402219 | 1.59E-08 |
| 54626 | 'HES2' | mRNA | -1.196058571 | 6.89E-04 |
| 5493 | 'PPL' | mRNA | -1.182310732 | 9.81E-56 |
| 55040 | 'EPN3' | mRNA | -1.348856422 | 1.70E-32 |
| 55076 | 'TMEM45A' | mRNA | -1.5360529 | 2.36E-07 |
| 55214 | 'P3H2' | mRNA | -1.395136926 | 5.02E-38 |
| 55222 | 'LRRC20' | mRNA | -1.189776238 | 3.51E-34 |
| 55287 | 'TMEM40' | mRNA | 1.071170177 | 1.87E-94 |
| 55454 | 'CSGALNACT2' | mRNA | 1.286051059 | 1.87E-10 |
| 55503 | 'TRPV6' | mRNA | -1.688055994 | 2.41E-10 |
| 5554 | 'PRH1' | mRNA | 1.060356469 | 8.78E-04 |
| 55640 | 'FLVCR2' | mRNA | -1.462343214 | 3.02E-07 |
| 55647 | 'RAB20' | mRNA | -1.323760494 | 1.01E-20 |
| 55924 | 'INKA2' | mRNA | 1.490527699 | 1.90E-05 |
| 55959 | 'SULF2' | mRNA | -1.272059407 | 9.09E-99 |
| 56006 | 'SMG9' | mRNA | 1.020845589 | 6.43E-185 |
| 5608 | 'MAP2K6' | mRNA | -2.198389992 | 1.77E-05 |
| 56169 | 'GSDMC' | mRNA | -1.934422702 | 1.40E-05 |
| 56246 | 'MRAP' | mRNA | 2.617689278 | 6.52E-05 |
| 5649 | 'RELN' | mRNA | 2.36923381 | 2.55E-05 |
| 56892 | 'TCIM' | mRNA | -1.898120386 | 2.76E-07 |
| 56895 | 'AGPAT4' | mRNA | -1.345135486 | 4.80E-05 |
| 56901 | 'NDUFA4L2' | mRNA | 1.784271309 | 4.23E-04 |
| 56971 | 'CEACAM19' | mRNA | -1.049143595 | 1.26E-14 |
| 57118 | 'CAMK1D' | mRNA | -1.322826838 | 3.05E-14 |
| 57158 | 'JPH2' | mRNA | -2.123123735 | 3.39E-54 |
| 57326 | 'PBXIP1' | mRNA | -1.030411397 | 2.45E-12 |
| 57348 | 'TTYH1' | mRNA | -1.102170324 | 1.26E-06 |
| 57460 | 'PPM1H' | mRNA | -1.360631624 | 3.00E-07 |
| 57491 | 'AHRR' | mRNA | 1.067249218 | 2.55E-07 |
| 57509 | 'MTUS1' | mRNA | -1.27376625 | 1.52E-13 |
| 57523 | 'NYNRIN' | mRNA | -1.157541277 | 8.77E-05 |
| 57530 | 'CGN' | mRNA | -1.135251917 | 1.08E-27 |
| 57688 | 'ZSWIM6' | mRNA | 1.224560258 | 3.15E-15 |
| 57710 | 'KIAA1614' | mRNA | 1.096811224 | 1.23E-06 |
| 5837 | 'PYGM' | mRNA | 4 | 1.29E-15 |
| 586 | 'BCAT1' | mRNA | -1.200584881 | 9.82E-74 |
| 5913 | 'RAPSN' | mRNA | 1.571906348 | 1.00E-10 |
| 5918 | 'RARRES1' | mRNA | -2.032421478 | 9.68E-05 |
| 5920 | 'PLAAT4' | mRNA | -1.442943496 | 3.08E-05 |
| 59344 | 'ALOXE3' | mRNA | 1.453840217 | 3.93E-57 |
| 613037 | 'NPIPB13' | mRNA | 1.281452106 | 2.40E-39 |
| 6273 | 'S100A2' | mRNA | 1.12151782 | 1.55E-21 |
| 6275 | 'S100A4' | mRNA | -1.108524457 | 3.77E-05 |
| 6279 | 'S100A8' | mRNA | -1.298844482 | 2.46E-07 |
| 6288 | 'SAA1' | mRNA | -1.281666877 | 2.26E-23 |
| 6289 | 'SAA2' | mRNA | -1.260495638 | 2.40E-07 |
| 629 | 'CFB' | mRNA | -1.040045552 | 1.87E-94 |
| 6291 | 'SAA4' | mRNA | -10.35204343 | 9.52E-04 |
| 6319 | 'SCD' | mRNA | -1.716293776 | 2.58E-219 |
| 6337 | 'SCNN1A' | mRNA | -1.251633322 | 5.74E-105 |
| 634 | 'CEACAM1' | mRNA | -2.040193019 | 7.25E-10 |
| 6348 | 'CCL3' | mRNA | 3.089754653 | 4.48E-04 |
| 6404 | 'SELPLG' | mRNA | 1.584962501 | 6.37E-11 |
| 64081 | 'PBLD' | mRNA | 1.225168943 | 2.42E-09 |
| 64115 | 'VSIR' | mRNA | -1.179911364 | 1.35E-28 |
| 643161 | 'FAM25A' | mRNA | 1.373549986 | 5.37E-09 |
| 6446 | 'SGK1' | mRNA | 1.158545939 | 1.70E-27 |
| 64577 | 'ALDH8A1' | mRNA | 1.406606825 | 8.04E-04 |
| 64757 | 'MTARC1' | mRNA | -1.368761725 | 5.02E-17 |
| 64759 | 'TNS3' | mRNA | -1.428553812 | 1.60E-85 |
| 648 | 'BMI1' | mRNA | 10.96794671 | 1.66E-06 |
| 6507 | 'SLC1A3' | mRNA | -1.905535316 | 5.09E-22 |
| 6515 | 'SLC2A3' | mRNA | -3.044527807 | 1.01E-08 |
| 65266 | 'WNK4' | mRNA | 1.38323701 | 2.71E-14 |
| 653269 | 'POTEI' | mRNA | 1.684319058 | 6.50E-05 |
| 653404 | 'FOXD4L6' | mRNA | 10.27029533 | 4.51E-04 |
| 653567 | 'TMEM236' | mRNA | 2.244690593 | 2.36E-30 |
| 653781 | 'POTEJ' | mRNA | -1.84884331 | 1.96E-06 |
| 654346 | 'LGALS9C' | mRNA | -10.56319627 | 2.93E-04 |
| 654483 | 'BOLA2B' | mRNA | 1.067342025 | 1.23E-14 |
| 6578 | 'SLCO2A1' | mRNA | -1.735120144 | 6.34E-23 |
| 6591 | 'SNAI2' | mRNA | 1.170826968 | 1.77E-05 |
| 65983 | 'GRAMD2B' | mRNA | -1.022838308 | 8.00E-06 |
| 6653 | 'SORL1' | mRNA | -1.517058436 | 2.08E-05 |
| 6698 | 'SPRR1A' | mRNA | 2.195852856 | 2.97E-59 |
| 6699 | 'SPRR1B' | mRNA | 1.807217623 | 0 |
| 6710 | 'SPTB' | mRNA | -1.240008965 | 6.53E-05 |
| 6781 | 'STC1' | mRNA | -2 | 2.87E-06 |
| 6861 | 'SYT5' | mRNA | 1.682107418 | 1.02E-17 |
| 6932 | 'TCF7' | mRNA | 1.626689626 | 1.15E-12 |
| 6948 | 'TCN2' | mRNA | -1.359137366 | 3.03E-18 |
| 7052 | 'TGM2' | mRNA | -1.937392539 | 0 |
| 7087 | 'ICAM5' | mRNA | -1.091237499 | 2.43E-11 |
| 7089 | 'TLE2' | mRNA | -1.316027493 | 2.67E-11 |
| 7124 | 'TNF' | mRNA | -1.191695624 | 1.38E-08 |
| 7127 | 'TNFAIP2' | mRNA | -1.042362924 | 1.38E-170 |
| 715 | 'C1R' | mRNA | -1.342392197 | 8.31E-20 |
| 716 | 'C1S' | mRNA | -1.347504342 | 5.86E-29 |
| 718 | 'C3' | mRNA | -1.881712306 | 0 |
| 721 | 'C4B' | mRNA | 11.19044202 | 1.97E-07 |
| 723790 | 'H2AC19' | mRNA | 14.70498432 | 1.00E-85 |
| 727851 | 'RGPD8' | mRNA | 2.934460997 | 1.30E-12 |
| 727897 | 'MUC5B' | mRNA | -2.432959407 | 8.10E-07 |
| 728441 | 'GGT2' | mRNA | 1.025036906 | 2.16E-05 |
| 729597 | 'SPDYE6' | mRNA | 2.028162354 | 2.55E-06 |
| 729877 | 'TBC1D3H' | mRNA | 1.948626997 | 8.06E-10 |
| 7378 | 'UPP1' | mRNA | 1.461135684 | 0 |
| 7429 | 'VIL1' | mRNA | -11.84862294 | 9.30E-10 |
| 7474 | 'WNT5A' | mRNA | 1.023458973 | 3.77E-07 |
| 7475 | 'WNT6' | mRNA | -1.549034354 | 8.00E-12 |
| 7483 | 'WNT9A' | mRNA | -1.198864444 | 2.91E-28 |
| 752 | 'FMNL1' | mRNA | -1.005951772 | 1.22E-21 |
| 768 | 'CA9' | mRNA | -3.557995453 | 6.86E-19 |
| 780776 | 'TVP23A' | mRNA | 1.578508791 | 2.12E-05 |
| 79008 | 'SLX1B' | mRNA | -12.4314976 | 7.67E-15 |
| 79025 | 'FNDC11' | mRNA | -1.5360529 | 2.36E-07 |
| 79026 | 'AHNAK' | mRNA | -1.055233382 | 6.77E-123 |
| 79140 | 'CCDC28B' | mRNA | -1.371234208 | 2.62E-10 |
| 79170 | 'PRR15L' | mRNA | -2.688055994 | 2.00E-30 |
| 79365 | 'BHLHE41' | mRNA | -1.08917051 | 2.36E-10 |
| 79412 | 'KREMEN2' | mRNA | -1.012791553 | 1.74E-21 |
| 79413 | 'ZBED2' | mRNA | -2.38466385 | 6.67E-06 |
| 7942 | 'TFEB' | mRNA | -1.020464103 | 1.28E-04 |
| 79541 | 'OR2A4' | mRNA | 11.50481899 | 2.73E-09 |
| 79603 | 'CERS4' | mRNA | -1.574874403 | 2.21E-17 |
| 79674 | 'VEPH1' | mRNA | -1.351472371 | 3.48E-04 |
| 79689 | 'STEAP4' | mRNA | -1.516501545 | 2.69E-33 |
| 79774 | 'GRTP1' | mRNA | -1.05250772 | 3.17E-14 |
| 79817 | 'MOB3B' | mRNA | -1.069421401 | 3.53E-05 |
| 79827 | 'CLMP' | mRNA | 1.142356216 | 1.50E-08 |
| 79888 | 'LPCAT1' | mRNA | -1.076050554 | 2.27E-137 |
| 79901 | 'CYBRD1' | mRNA | -1.110499541 | 2.25E-14 |
| 79919 | 'MAB21L4' | mRNA | -2.124676738 | 2.73E-60 |
| 79956 | 'ERMP1' | mRNA | -1.096878776 | 2.53E-12 |
| 79987 | 'SVEP1' | mRNA | -1.187202993 | 1.52E-07 |
| 80117 | 'ARL14' | mRNA | 1.189477799 | 6.07E-09 |
| 80221 | 'ACSF2' | mRNA | -1.180445077 | 1.91E-51 |
| 80303 | 'EFHD1' | mRNA | -1 | 3.20E-05 |
| 80326 | 'WNT10A' | mRNA | -1.841218374 | 1.28E-13 |
| 8061 | 'FOSL1' | mRNA | 1.026745114 | 6.12286235395E-313 |
| 80704 | 'SLC19A3' | mRNA | -1.059920451 | 4.59E-04 |
| 80723 | 'SLC35G2' | mRNA | 1.06700977 | 1.90E-08 |
| 80726 | 'IQCN' | mRNA | 2.925999419 | 1.30E-07 |
| 8076 | 'MFAP5' | mRNA | -5.558816532 | 9.52E-04 |
| 80781 | 'COL18A1' | mRNA | -1.459128576 | 1.52E-160 |
| 80824 | 'DUSP16' | mRNA | 1.100321658 | 8.77E-30 |
| 8110 | 'DPF3' | mRNA | 2.387023123 | 8.10E-11 |
| 81543 | 'LRRC3' | mRNA | -1.131084313 | 1.78E-04 |
| 81606 | 'LBH' | mRNA | -3 | 3.61E-04 |
| 81792 | 'ADAMTS12' | mRNA | -1.389946518 | 1.90E-04 |
| 828 | 'CAPS' | mRNA | -1.451728568 | 1.86E-11 |
| 8322 | 'FZD4' | mRNA | -1.223675481 | 4.30E-16 |
| 8337 | 'H2AC18' | mRNA | -13.83861304 | 1.34E-39 |
| 83401 | 'ELOVL3' | mRNA | -1.903369726 | 4.62E-12 |
| 83439 | 'TCF7L1' | mRNA | -1.53963851 | 9.28E-17 |
| 83445 | 'GSG1' | mRNA | 1.088608292 | 8.78E-04 |
| 83452 | 'RAB33B' | mRNA | 1.388859385 | 2.55E-06 |
| 83667 | 'SESN2' | mRNA | 1.339936044 | 2.40E-39 |
| 83706 | 'FERMT3' | mRNA | 1.511387242 | 4.90E-10 |
| 83740 | 'H2AB3' | mRNA | 10.10328781 | 9.01E-04 |
| 8416 | 'ANXA9' | mRNA | -1.395439363 | 1.90E-14 |
| 84189 | 'SLITRK6' | mRNA | -2.28253865 | 4.18E-08 |
| 84220 | 'RGPD5' | mRNA | 1.03825492 | 1.39E-05 |
| 84230 | 'LRRC8C' | mRNA | 1.404517153 | 1.06E-10 |
| 8437 | 'RASAL1' | mRNA | -1.261265313 | 2.58E-07 |
| 84419 | 'C15orf48' | mRNA | -1.388219774 | 1.83E-40 |
| 84441 | 'MAML2' | mRNA | -1.19354623 | 8.99E-04 |
| 84465 | 'MEGF11' | mRNA | 1.513910626 | 9.08E-04 |
| 84532 | 'ACSS1' | mRNA | -1.552940039 | 2.55E-26 |
| 846 | 'CASR' | mRNA | 1.181692729 | 3.17E-04 |
| 84693 | 'MCEE' | mRNA | -1.278535499 | 3.51E-06 |
| 84795 | 'PYROXD2' | mRNA | -1.232102546 | 1.71E-13 |
| 84803 | 'GPAT3' | mRNA | 1.091499988 | 7.98E-54 |
| 84842 | 'HPDL' | mRNA | -1.222392421 | 5.19E-10 |
| 84941 | 'HSH2D' | mRNA | -1.220198872 | 3.74E-18 |
| 8495 | 'PPFIBP2' | mRNA | -1.832890014 | 4.39E-05 |
| 84958 | 'SYTL1' | mRNA | -1.154022934 | 3.38E-09 |
| 85360 | 'SYDE1' | mRNA | -1.056955885 | 1.30E-09 |
| 85366 | 'MYLK2' | mRNA | 2.192532363 | 8.14E-18 |
| 85376 | 'RIMBP3' | mRNA | 1.06771945 | 3.67E-05 |
| 85462 | 'FHDC1' | mRNA | -1.116855233 | 1.37E-06 |
| 8564 | 'KMO' | mRNA | -1.702821622 | 5.46E-19 |
| 8581 | 'LY6D' | mRNA | -2.355050298 | 4.22E-130 |
| 8614 | 'STC2' | mRNA | 1.129727719 | 2.34E-237 |
| 867 | 'CBL' | mRNA | 1.050192473 | 7.32E-07 |
| 8681 | 'JMJD7-PLA2G4B' | mRNA | -11.02583167 | 1.51E-05 |
| 8701 | 'DNAH11' | mRNA | -1.57305362 | 3.92E-05 |
| 8796 | 'SCEL' | mRNA | -1.132028394 | 2.38E-09 |
| 8817 | 'FGF18' | mRNA | 4.247927513 | 3.34E-05 |
| 8848 | 'TSC22D1' | mRNA | 1.315585207 | 0 |
| 8851 | 'CDK5R1' | mRNA | 1.0054758 | 5.97E-05 |
| 8876 | 'VNN1' | mRNA | -1.747058823 | 2.70E-06 |
| 8908 | 'GYG2' | mRNA | -1.750021747 | 5.65E-06 |
| 8938 | 'BAIAP3' | mRNA | -1.051203341 | 5.80E-04 |
| 89780 | 'WNT3A' | mRNA | -2.415037499 | 2.53E-04 |
| 89795 | 'NAV3' | mRNA | 1.150474815 | 2.65E-23 |
| 89846 | 'FGD3' | mRNA | -2.777122721 | 1.60E-57 |
| 8991 | 'SELENBP1' | mRNA | -1.189033824 | 6.50E-05 |
| 90019 | 'SYT8' | mRNA | -4.95419631 | 2.73E-07 |
| 9022 | 'CLIC3' | mRNA | -1.988318195 | 1.04E-21 |
| 90693 | 'CCDC126' | mRNA | 1.423211431 | 1.62E-05 |
| 9076 | 'CLDN1' | mRNA | -1.040128113 | 7.82E-12 |
| 91156 | 'IGFN1' | mRNA | 1.675886285 | 3.37E-133 |
| 9127 | 'P2RX6' | mRNA | -1.148786234 | 4.73E-04 |
| 91584 | 'PLXNA4' | mRNA | -3.624490865 | 4.39E-07 |
| 91624 | 'NEXN' | mRNA | 1.086179875 | 4.86E-06 |
| 91683 | 'SYT12' | mRNA | -1.416136696 | 1.43E-65 |
| 9173 | 'IL1RL1' | mRNA | 1.527499347 | 2.79E-04 |
| 9194 | 'SLC16A7' | mRNA | 1.029121203 | 6.35E-04 |
| 92126 | 'DSEL' | mRNA | 1.350468987 | 1.26E-04 |
| 9235 | 'IL32' | mRNA | -1.094517599 | 4.57E-07 |
| 9241 | 'NOG' | mRNA | -1.440555901 | 6.76E-26 |
| 93659 | 'CGB5' | mRNA | 2.368387406 | 4.80E-06 |
| 9389 | 'SLC22A14' | mRNA | 4 | 2.35E-04 |
| 93953 | 'GCNA' | mRNA | 2.125499573 | 2.71E-06 |
| 94027 | 'CGB7' | mRNA | 1.129691077 | 6.33E-06 |
| 94031 | 'HTRA3' | mRNA | -1.389946518 | 3.24E-15 |
| 94115 | 'CGB8' | mRNA | 1.951708683 | 1.01E-25 |
| 9462 | 'RASAL2' | mRNA | 1.156323853 | 1.20E-62 |
| 954 | 'ENTPD2' | mRNA | -1.012600037 | 7.37E-04 |
| 9586 | 'CREB5' | mRNA | 1.695689319 | 2.92E-25 |
| 9590 | 'AKAP12' | mRNA | 1.221031908 | 1.87E-56 |
| 9601 | 'PDIA4' | mRNA | -1.065102595 | 3.44E-288 |
| 9641 | 'IKBKE' | mRNA | -1.471840129 | 1.09E-48 |
| 96626 | 'LIMS3' | mRNA | -11.88760142 | 5.03E-10 |
| 9720 | 'CCDC144A' | mRNA | 1.190818303 | 1.77E-05 |
| 9721 | 'GPRIN2' | mRNA | -1.169159443 | 2.57E-05 |
| 9734 | 'HDAC9' | mRNA | 1.360720849 | 1.54E-17 |
| 9783 | 'RIMS3' | mRNA | -1.192634653 | 6.36E-08 |
| 9966 | 'TNFSF15' | mRNA | -1.170998489 | 3.74E-05 |

Differentially expressed genes in PC-9
